# Identification and validation of a previously missed mutational signature in colorectal cancer

**DOI:** 10.1038/s41467-026-76472-w

**Published:** 2026-08-12

**Authors:** Mariya Kazachkova, Burçak Otlu, Marcos Díaz-Gay, Ammal Abbasi, Sarah Moody, Zichen Jiang, Sandra Perdomo, David. C. Wedge, Paul Brennan, Michael. R. Stratton, Ludmil. B. Alexandrov

**Affiliations:** 1 Biomedical Sciences Graduate Program, University of California San Diego, La Jolla, CA USA; 2 Department of Cellular and Molecular Medicine, University of California San Diego, La Jolla, CA USA; 3 Moores Cancer Center, University of California San Diego, La Jolla, CA USA; 4 Department of Health Informatics, Graduate School of Informatics, Middle East Technical University, Ankara, Turkey; 5 Digital Genomics Group, Cancer Genomics Program, Spanish National Cancer Research Center (CNIO), Madrid, Spain; 6 Department of Bioengineering, University of California San Diego, La Jolla, CA USA; 7 Somatic Genomics Programme, Wellcome Sanger Institute, Cambridge, UK; 8 Genomic Epidemiology Branch, International Agency for Research on Cancer (IARC/WHO), Lyon, France; 9 Manchester Cancer Research Centre, University of Manchester, Manchester, UK; 10 Sanford Stem Cell Institute, University of California San Diego, La Jolla, CA USA

**Keywords:** Cancer genomics, Cancer genetics, Colorectal cancer, Mutation, DNA damage and repair

## Abstract

Mutational signature analysis has enhanced our understanding of mutagenic processes. In a recent study, we analyzed 802 microsatellite-stable colorectal cancers (CRC) and identified a de novo signature, SBS_D, which was decomposed into SBS18. Here, we re-evaluate this decomposition and provide evidence that SBS_D represents a distinct mutational process from SBS18. Through an analysis of 2,616 CRCs across three independent cohorts, we demonstrate that SBS_D is consistently present, suggesting this signature may have been previously overlooked. We illustrate that the pattern of SBS_D better aligns with signatures associated with deficiencies in DNA repair, despite evidence that SBS_D is not driven by canonical defects in these DNA repair pathways. Overall, this study identifies a previously unrecognized mutational signature in DNA repair-proficient CRC and proposes that its etiology may be linked to DNA repair infidelity emerging late in tumor development. SBS_D has been submitted to the COSMIC database and provisionally designated as SBS111.

## Abstract (web summary)

Mutational signature SBS_D had been previously identified in a large-scale genomics analysis of microsatellite-stable colorectal cancers (CRC), and had been decomposed into known mutational signatures. Here, the authors demonstrate in data from three CRC cohorts that SBS_D is an independent mutational signature that may be linked to DNA repair infidelity, and is now designated as SBS111.

## Introduction

Over the past decade, mutational signature analysis has become a standard component of genomics research^1,2^, enhancing our understanding of the extrinsic and intrinsic mutagenic factors that influence the genomes of both cancer^3^ and normal somatic cells^4^. This has resulted in the development of global reference sets of mutational signatures, including those maintained by the Catalog of Somatic Mutations in Cancer (COSMIC) database^5,6^.

In principle, mutational signature analysis can be performed in at least two ways: by assigning known reference signatures from databases like COSMIC to individual samples^7^ or by conducting de novo extraction of mutational signatures^8^. This latter approach is commonly employed in the analysis of large datasets containing at least several hundred samples, enabling the identification of novel mutational signatures by detecting de novo signatures that do not correspond to known references^8^. Specifically, all de novo extracted signatures are either directly matched to known reference signatures or, more frequently, decomposed into a combination of multiple reference signatures. Given the extensive catalog of known reference signatures, the decomposition process can be optimized to prioritize biologically relevant signatures, minimizing the risk of overfitting^9–11^. For example, a mutational signature associated with ultraviolet (UV) light exposure would typically be excluded from the decomposition of de novo signatures detected in colorectal or brain tumors as UV-induced mutations are not expected in these contexts^5^. This process of removing specific mutational signatures during decomposition based on prior biological knowledge is referred to as an optimized decomposition approach. This is in contrast with a naïve decomposition approach, where no COSMIC signatures are excluded while performing decomposition. Following decomposition, any signature that cannot be accurately reconstructed using a combination of known signatures with a cosine similarity of at least 0.90 is generally considered novel^9^.

In most cases, it is straightforward to determine whether a mutational signature is known or novel. Many de novo extracted signatures are easily decomposed into known reference signatures with high similarity. Conversely, some signatures exhibit patterns that cannot be fully reconstructed with substantial similarity, indicating the presence of a novel signature absent from the current reference set. Nevertheless, borderline cases exist where it remains uncertain whether a signature represents a truly distinct mutational process or merely a complex combination of previously known signatures. Scientifically, there is always a temptation to report a novel signature, as it suggests the discovery of a potentially novel mutagenic process. However, validating such a discovery requires robust evidence in independent cohorts, particularly for signatures with unknown etiology, to ensure their validity. As a result, studies may adopt a conservative approach, potentially leading to the underreporting of genuinely novel mutational signatures.

Here, we revisit a recent de novo mutational signature analysis of 802 whole-genome sequenced microsatellite-stable colorectal cancers (CRC) from the Mutographs project^9^. In the original analysis, we identified a de novo single-base substitution (SBS) signature, designated SBS_D, which we did not classify as a novel signature and instead decomposed into known COSMIC signatures^9^, including the reactive oxygen species-associated signature SBS18^12^. Here, we demonstrate that SBS_D represents a distinct mutational process from SBS18. Furthermore, we successfully extract SBS_D from three independent, previously published CRC cohorts^13–15^, none of which had identified or reported it. Finally, based on associations with known molecular phenotypes in CRC, we propose that SBS_D may result from POLD1 proofreading and mismatch repair infidelity occurring late in the evolution of tumors without detectable canonical defects in either pathway. Collectively, our findings reveal the existence of a previously unrecognized mutational signature in microsatellite-stable CRC samples and underscore the challenges associated with identifying novel mutational signatures.

## Results

### Original mutographs de novo signature analysis

**Fig. 1:**
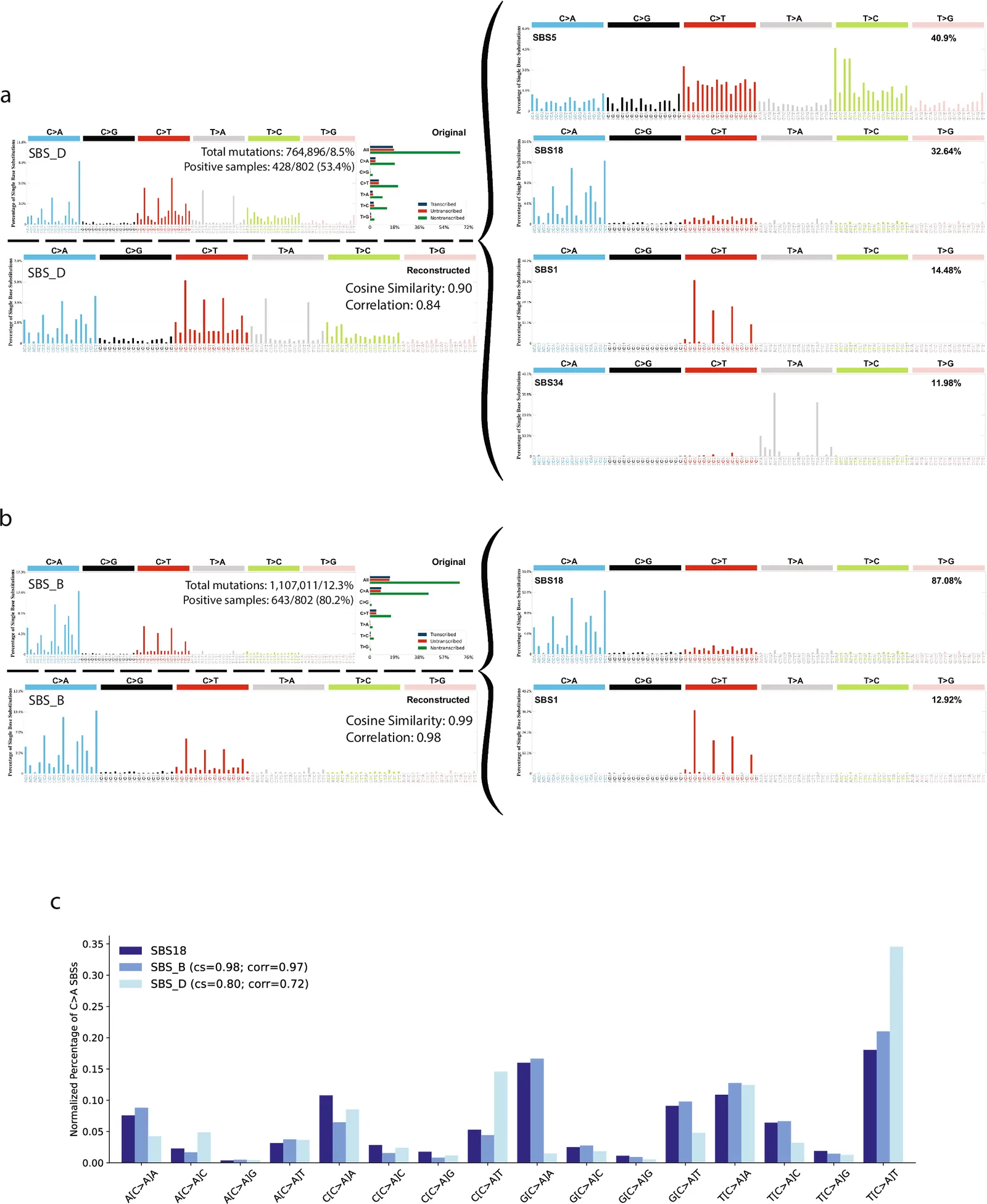
SBS_D and SBS_B from the mutographs colorectal cancer cohort. **a** Optimized decomposition of SBS_D showing the total number of mutations attributed to the signature and the proportion of samples in which it is detected. Cosine similarity and Spearman correlation are calculated between the original de novo signature and the reconstructed signature using the SBS-96 context. **b** Optimized decomposition of SBS_B showing the total number of mutations attributed to the signature and the proportion of samples in which it is detected. Cosine similarity and Spearman correlation are calculated between the original de novo signature and the reconstructed signature using the SBS-96 context. **c** Comparison of the C > A contexts among COSMIC signature SBS18, de novo signature SBS_B, and de novo signature SBS_D. SBS_B and SBS_D were collapsed to the SBS-96 mutation classification schema, and normalization was performed within the C > A contexts. Cosine similarity (cs) and Spearman correlation (corr) are shown relative to the C > A contexts for SBS18. Source data are provided as a Source Data file.

All analyses of mutational signatures intrinsically rely on a predefined schema for classifying somatic mutations^12^ (Methods). In the original analysis of 802 treatment-naïve, microsatellite-stable, DNA repair-proficient CRC samples from the Mutographs CRC study, de novo extraction was performed using the SBS-288 classification schema^9^. This resulted in the identification of 16 de novo mutational signatures, labeled SBS_A through SBS_P (note that the use of letters distinguishes these from COSMIC reference signatures, which typically use numbers, e.g., SBS18). Of these, 12 signatures were decomposed into COSMIC reference signatures, and the remaining four signatures were classified as novel since they represented mutational patterns not previously cataloged in COSMICv3.4. One of the 12 de novo signatures that was decomposed into COSMIC signatures, SBS_D, achieved a borderline reconstruction cosine similarity of 0.90. As no other large CRC mutational signature study had previously reported an SBS_D-like signature, we adopted a conservative approach and reported SBS_D as a mixture of previously known signatures while acknowledging the possibility that it may represent a novel signature^9^. Importantly, SBS_D was found to have non-zero activity in 428/802 microsatellite-stable CRC samples, contributing 8.5% of all mutations across the 802 samples (Fig. 1a). Among the 16 de novo mutational signatures identified in the Mutographs CRC cohort, SBS_D ranked fourth in total mutational contribution. Additionally, in the 428 samples with non-zero SBS_D activity, SBS_D contributed 16% of all mutations. Thus, although it contributes fewer mutations than the most ubiquitous CRC processes, SBS_D still represents a major contributor to mutagenesis in DNA repair-proficient CRC samples.

### Reevaluating the pattern of SBS_D

The pattern of SBS_D was decomposed into COSMICv3.4 signatures SBS1 (14.5%), SBS5 (40.9%), SBS18 (32.6%), and SBS34 (12.0%) (Fig. 1a). SBS1 and SBS5 are ubiquitous clock-like mutational signatures present in all types of cancer^12^ and normal tissues^16^. Due to their pervasive nature, some degree of SBS1 and SBS5 contamination is expected in the decomposition of any de novo signature^8^. As such, if the decomposition was accurate, SBS_D would represent a combination of the reactive oxygen species-associated signature SBS18, the unknown etiology signature SBS34, and contributions from ubiquitous clock-like signatures (SBS1 and SBS5), with SBS18 being the predominant non-clock-like contributor. However, another de novo signature extracted from this cohort, SBS_B, exhibited a near perfect match to SBS18 (Fig. 1b). Moreover, the C > A component, which constitutes the majority of SBS18 mutations, showed substantial differences between SBS18 and SBS_D (cosine similarity [CS] = 0.80; Spearman correlation [CORR] = 0.72) but was nearly identical between SBS18 and SBS_B (CS = 0.98; CORR = 0.97; Fig. 1c). Considering the high similarity between SBS18 and SBS_B, we will henceforth refer to SBS_B as SBS18^. Additionally, as part of the Mutographs study, additional extractions were performed using the SBS-96 and SBS-1536 classification schemas. Both approaches yielded two signatures closely resembling SBS18^ and SBS_D (Supplementary Fig. 1). Furthermore, analyses with two independent signature extraction tools—MuSiCal^17^ and SignatureToolsLib^18^—also identified signatures highly similar to SBS18^ and SBS_D (Supplementary Fig. 2). This consistency across different mutational schemas and different extraction tools highlights the robustness of the patterns for these signatures, irrespective of the underlying classification framework. Furthermore, we observed that SBS_D was dissimilar to any known COSMIC^6^ or Signal^19^ mutational signatures (cosine similarities <0.81 across all signatures). Overall, these findings raised doubts about the assignment of SBS18 as a contributor to SBS_D and suggested that SBS_D could represent a distinct mutational process.

### SBS_D represents a mutational process distinct from SBS18

**Fig. 2:**
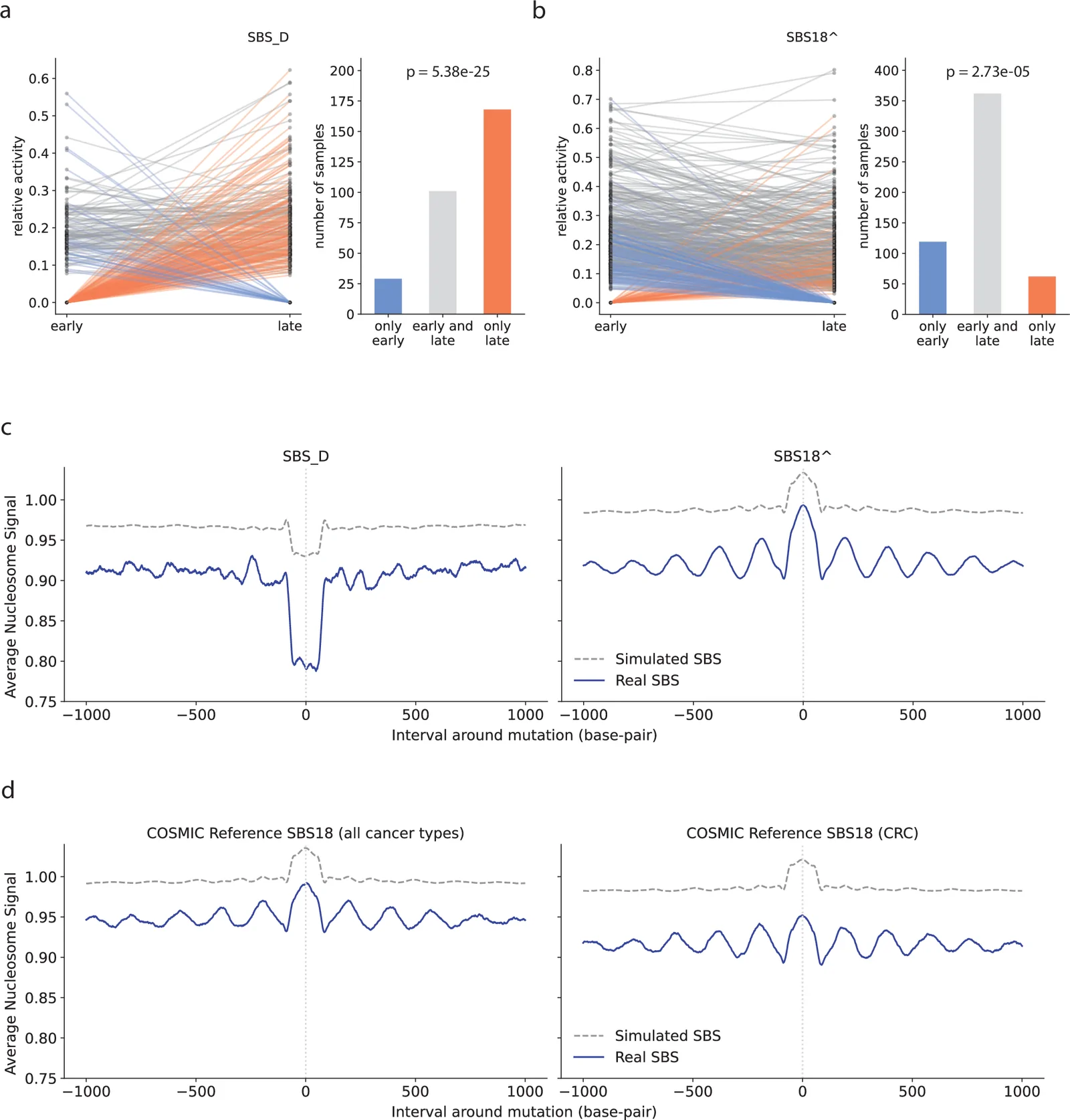
SBS_D and SBS18^ reflect distinct mutational processes. **a** (Left) Relative SBS_D activity in early and late clonal mutations in the Mutographs colorectal cancer cohort identified using Mutational Signature Attribution (MSA). Each line represents one sample: gray lines indicate SBS_D activity in both early and late clonal mutations, blue lines indicate activity only in early clonal mutations, and red lines indicate activity only in late clonal mutations. (Right) Number of samples with SBS_D activity in each category. Only samples with non-zero SBS_D activity in either early or late clonal mutations are included in the plots (298/684). *P* value is based on a two-sided McNemar test. **b** Same format as (**a**), but for SBS18^, showing a different pattern of activity across early and late clonal mutations. Only samples with non-zero SBS_B activity in either early or late clonal mutations are included in the plots (543/684). **c** Nucleosome occupancy pattern for SBS_D (left) and SBS18^ (right), as identified by SigProfilerTopography. Each plot is centered on the mutation site, with 1000 base pairs upstream (5’) and downstream (3’). Solid lines represent the average nucleosome signal for observed single-base substitution (SBS) mutations; dashed lines show the signal for simulated mutations. **d** Nucleosome occupancy patterns for COSMIC signature SBS18 across all cancer types (left) and restricted to colorectal cancer (CRC) samples (right) from the COSMIC website. Plot features and layout match those in (**c**). Source data are provided as a Source Data file.

To enhance our understanding of SBS_D and its relationship with SBS18^, we performed molecular timing on the Mutographs CRC samples where copy number information was available (774 of 802 samples; 96.5%) by categorizing SBS mutations based on their timing relative to copy number gains during cancer development into two groups: early clonal and late clonal (Methods). This stratification allowed us to analyze the contributions of SBS_D and SBS18^ across distinct stages of tumor evolution. Our analysis revealed that SBS_D was preferentially active in late clonal mutations compared to early clonal mutations (*p* value < 0.0001; Fig. 2a). In contrast, SBS18^ was typically active exclusively early or both early and late but was rarely observed exclusively in late clonal mutations (*p* value < 0.0001, Fig. 2b). Notably, prior studies have similarly demonstrated that SBS18 is either preferentially early or constant in CRC development^20,21^. These findings indicate that SBS_D is an evolutionarily late signature, likely representing a mutational process distinct from that of SBS18^.

Additionally, we performed topography analysis on the Mutographs CRC data to elucidate the topographical characteristics of SBS_D and SBS18^ (Methods). Although the two mutational signatures were similar in replication timing and strand asymmetry analyses (Supplementary Fig. 3), they displayed a notable difference in nucleosome occupancy (Fig. 2c). Specifically, SBS_D mutations were depleted near nucleosomes and did not exhibit any periodicity. In contrast, SBS18^ mutations were enriched near nucleosomes and demonstrated clear periodicity, as expected and previously reported^22^ for COSMIC signature SBS18 in CRC and across all cancer types (Fig. 2d). Of note, strong depletion at nucleosome-bound regions is a relatively rare behavior for SBS mutational signatures, with only 11/48 previously examined SBS signatures displaying this pattern^22^. These observations further highlight the clear similarity between SBS18^ and SBS18, and they suggest that SBS_D represents a mutational process dissimilar to the one captured by SBS18.

### Signatures SBS_D and SBS18^ are validated in independent cohorts

Although several large mutational signature extraction studies in CRC have been recently conducted^14,15^, none have reported a de novo signature resembling SBS_D. These prior studies analyzed DNA repair-proficient and repair-deficient samples together, which may have masked the presence of an SBS_D-like signature. Consistent with this, when we performed signature extraction on all Mutographs CRC samples without stratifying by DNA repair status (*n* = 981), no signature bearing resemblance to SBS_D was observed (CS < 0.85 across all 27 de novo signatures; Supplementary Fig. 4).

**Fig. 3:**
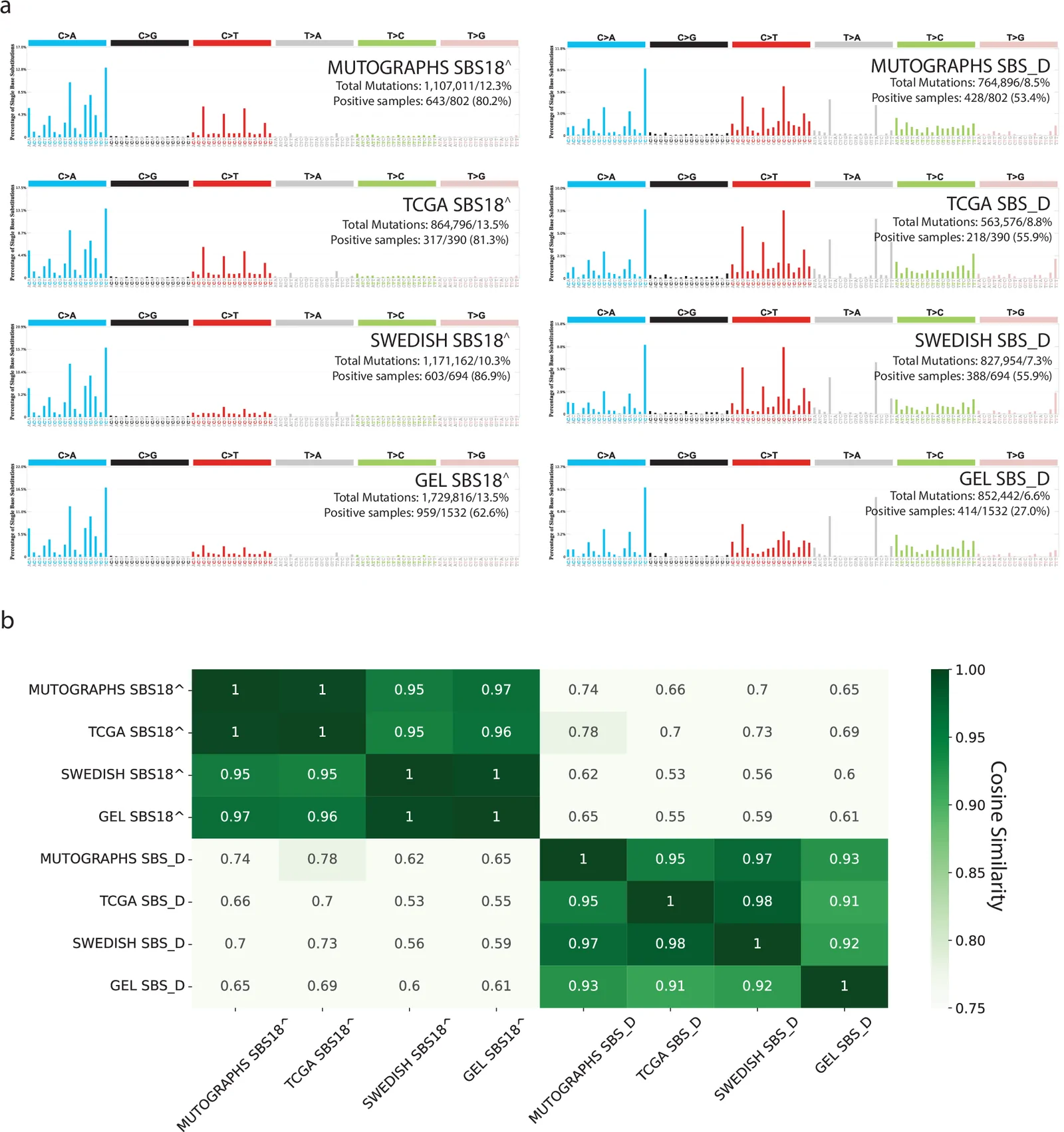
Independent colorectal cancer cohorts support reproducibility of SBS_D and SBS18^. **a** SBS18^ (left) and SBS_D (right) signatures extracted from the Mutographs, The Cancer Genome Atlas (TCGA), Swedish, and Genomics England (GEL) colorectal cancer (CRC) cohorts using SigProfilerExtractor. Signature extraction for the Mutographs, TCGA, and Swedish cohorts was performed using the SBS-288 context and then collapsed to SBS-96 for visualization; the GEL cohort was directly analyzed in the SBS-96 context. The number of mutations and positive samples reflects results from Mutational Signature Attribution (MSA) using the de novo signatures identified by SigProfilerExtractor in each respective cohort. **b** Heatmap showing cosine similarities among SBS18^-like and SBS_D-like signatures extracted from the Mutographs, TCGA, Swedish, and GEL colorectal cancer cohorts. Source data are provided as a Source Data file.

To validate SBS_D and confirm its distinction from SBS18^, we performed de novo signature extraction on treatment-naïve, microsatellite-stable, DNA repair-proficient samples from three independent whole-genome sequenced CRC cohorts, including: (i) 390 recently whole-genome re-sequenced samples from The Cancer Genome Atlas (TCGA) project, representing patients predominantly from the United States^13^, (ii) 694 samples from a recently published study^14^, representing patients from Sweden; and (iii) 1532 samples from the Genomics England (GEL) project^15^, representing patients from the United Kingdom (Methods). We note that no overlapping samples were present among any of these cohorts. De novo extractions for the Swedish and TCGA cohorts were conducted using the SBS-288 classification schema, consistent with the approach used in the Mutographs CRC project^9^. In contrast, the GEL cohort was analyzed using the SBS-96 schema, as it is the only publicly available mutational context for these cancers^18^. Despite differences in classification schemas, SBS_D and SBS18^ were identified across all three cohorts with high cosine similarity to their original Mutographs CRC signatures, reinforcing their distinction and confirming the reproducibility of SBS_D extraction in independent DNA repair-proficient cohorts (Fig. 3a, b). Moreover, aside from the GEL cohort—which showed lower signature prevalence—the total number of mutations attributed to each signature, the proportion of samples with non-zero activity of each signature, and the proportion of mutations attributed to each signature within samples were very similar across the cohorts, further supporting their robustness and consistency (Fig. 3a; Supplementary Fig. 5; Supplementary Data 1–8).

### Investigating features associated with SBS_D+ samples

Of note, we investigated associations between SBS_D activity and standard clinical and pathological features, such as age of diagnosis, tumor subsite, or tumor stage (Methods). Across both binary and continuous modeling approaches, a modest positive association between SBS_D activity and age at diagnosis was the only relationship observed in more than one cohort (Supplementary Figs. 6 and 7). No other clinicopathological features demonstrated reproducible associations with SBS_D activity across cohorts. Similarly, mutations in previously identified driver genes^9^ were not associated with SBS_D (Supplementary Figs. 6d and  7d).

To further explore the etiology of SBS_D, we performed differential gene expression analysis comparing SBS_D+ and SBS_D- samples in the Swedish and TCGA cohorts with available RNA-seq data (Methods; Supplementary Fig. 8). Gene set enrichment analysis identified eight pathways consistently enriched among overexpressed genes in SBS_D+ samples across both cohorts, primarily related to cell-cycle regulation and biosynthetic processes (Supplementary Fig. 9; Supplementary Data 9–15). In contrast, no pathways were consistently enriched among underexpressed genes across cohorts (Supplementary Fig. 10; Supplementary Data 16–22).

Together, these results indicate reproducible but modest transcriptional differences associated with SBS_D. The limited magnitude of pathway-level perturbations suggests that the observed enrichments should be interpreted cautiously, particularly in the context of the relatively small effect sizes detected.

### Naive decomposition of SBS_D

**Fig. 4:**
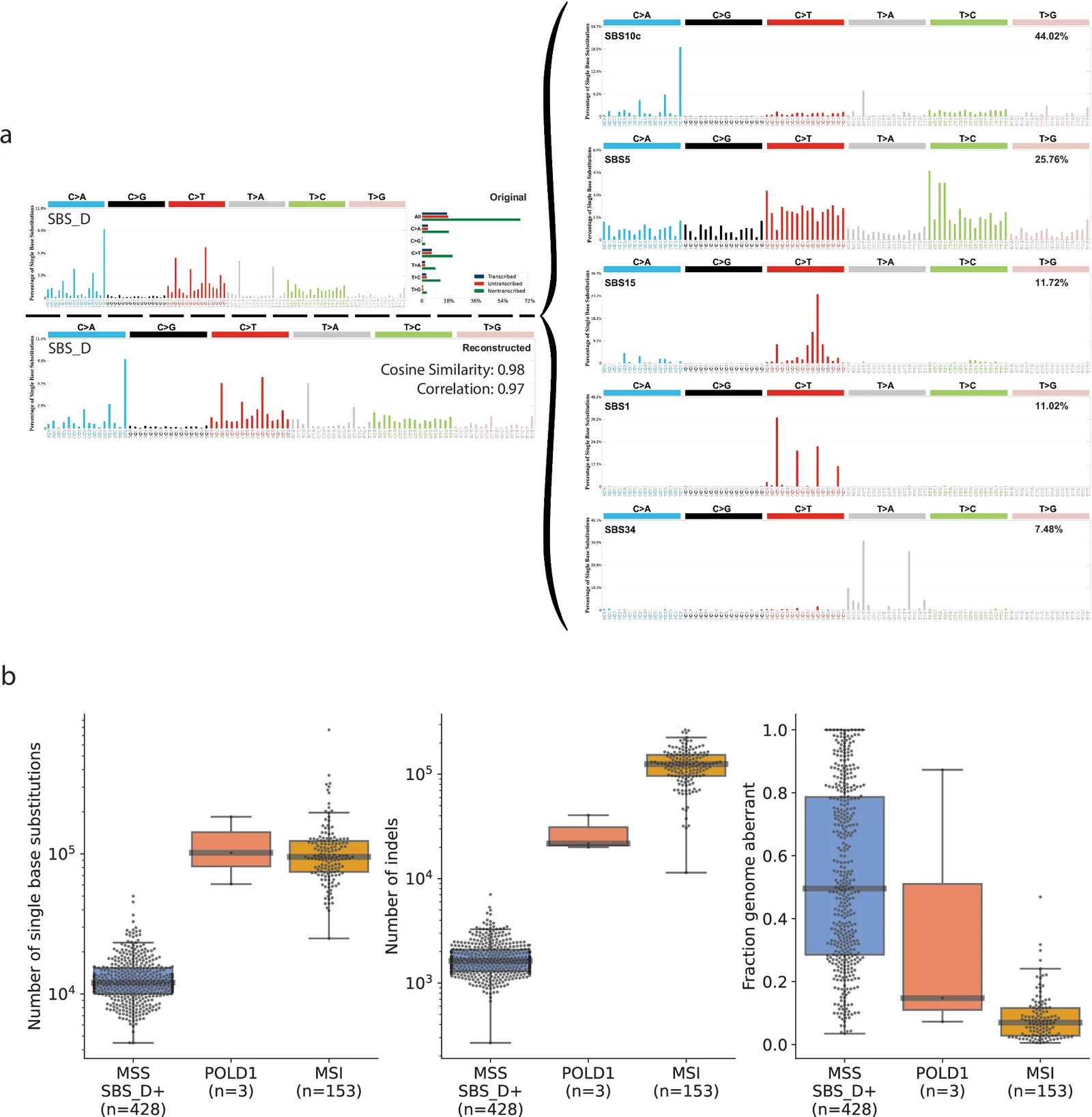
Naive decomposition of SBS_D and its distinction from *POLD1*-mutant and MSI tumors. **a** Naive decomposition, which allows the use of the complete COSMIC reference signature set, of SBS_D. Cosine similarity and Spearman correlation are calculated between the original de novo signature and the reconstructed signature using the SBS-96 context. **b** Comparison of microsatellite-stable samples with SBS_D activity (MSS SBS_D + ), *POLD1*-mutant samples, and samples harboring microsatellite instability (MSI) in the Mutographs colorectal cancer cohort in terms of total number of single base substitutions (SBSs; left), small insertions and deletions (indels; middle), and fraction genome aberrant (right). Box plots show the median (center line) and the 25th−75th percentiles (box bounds); whiskers extend to the most extreme data points within 1.5 times the interquartile range. All data points are shown as dots, including outliers. Source data are provided as a Source Data file.

The Mutographs CRC study utilized an optimized decomposition method when decomposing de novo signatures to COSMICv3.4 signatures^9^. Specifically, since the analyzed samples were treatment-naïve, microsatellite-stable, and DNA repair-proficient, the optimized decomposition process was designed to exclude mutational signatures associated with chemotherapy exposure or DNA repair deficiencies. In the naïve decomposition approach, which does not exclude any signatures and allows all COSMIC signatures to be utilized in the decomposition, SBS_D decomposed to a mixture of SBS10c (44%), SBS15 (12%), SBS34 (7%), and the clock-like signatures (37%) with cosine similarity of 0.98 (Fig. 4a). SBS10c has been shown to strictly come from samples harboring germline or somatic *POLD1* exonuclease domain mutations^23^, and SBS15 has been attributed to defective DNA mismatch repair in microsatellite-unstable samples^12^.

At first glance, these results suggest that SBS_D is associated with POLD1-deficiency or microsatellite instability. However, there are at least three lines of evidence that argue against this result. First, all *POLD1*-mutant and microsatellite-unstable CRC samples—confirmed by droplet digital PCR—were excluded from the 802-sample cohort prior to signature extraction. We also thoroughly reviewed all samples for any previously missed pathogenic or potentially pathogenic mutations in *POLD1* or the mismatch repair-associated genes. Given that SBS10c has only been observed in tumors with pathogenic germline or somatic mutations in *POLD1*’s exonuclease domain^23^, the absence of such mutations in the 802 samples argues against POLD1 deficiency as the cause of SBS_D (Supplementary Data 23 and 24). Although 12 samples carried non-pathogenic *POLD1* mutations, none showed elevated SBS_D activity (Supplementary Fig. 11a). Similarly, among the 38 samples with mutations in known mismatch repair genes, SBS_D activity was not elevated (Supplementary Fig. 11b; Supplementary Data 23 and 24). Only one CRC sample was found to carry a pathogenic *MSH6* nonsense mutation, but it lacked both SBS_D activity and a mutational profile consistent with microsatellite instability (Supplementary Fig. 12). Similarly, we observed no difference in SBS_D activity between samples with and without loss of heterozygosity in *POLD1* or DNA mismatch repair genes (*POLD1*: 103/774; DNA mismatch repair: 420/774; Supplementary Fig. 11c, d). These results indicate that the samples used for the extraction of SBS_D do not harbor known or detectable deficiencies in DNA repair pathways. Second, while samples with known *POLD1* mutations or microsatellite instability showed 10- to 100-fold higher substitution and indel rates, this elevated mutational burden was not observed in microsatellite-stable samples positive for SBS_D (Fig. 4b). Third, tumors with pathogenic *POLD1* mutations or microsatellite instability typically have stable, diploid genomes with aberrations affecting less than 20% of the genome, which is in stark contrast to the substantial genomic instability observed in most tumors with SBS_D activity (Fig. 4b). In addition to our observations in the Mutographs CRC cohort, previous studies^24,25^ have also noted that microsatellite unstable samples and samples with proofreading deficiencies have higher single base substitution and indel burdens but lower genome aberration compared to DNA repair-proficient tumors. While microsatellite instability—but not POLD1 deficiency—can also arise from epigenetic events, mainly the hypermethylation of the *MLH1* promoter^26^, such data were not available for the Mutographs cohort. However, epigenetically driven cases also exhibit high levels of substitutions and indels^27^, which were not observed in SBS_D-positive samples. Together, this supports the conclusion that SBS_D is not driven by canonical defects in POLD1 or mismatch repair.

### Exploring the potential etiology of SBS_D

**Fig. 5:**
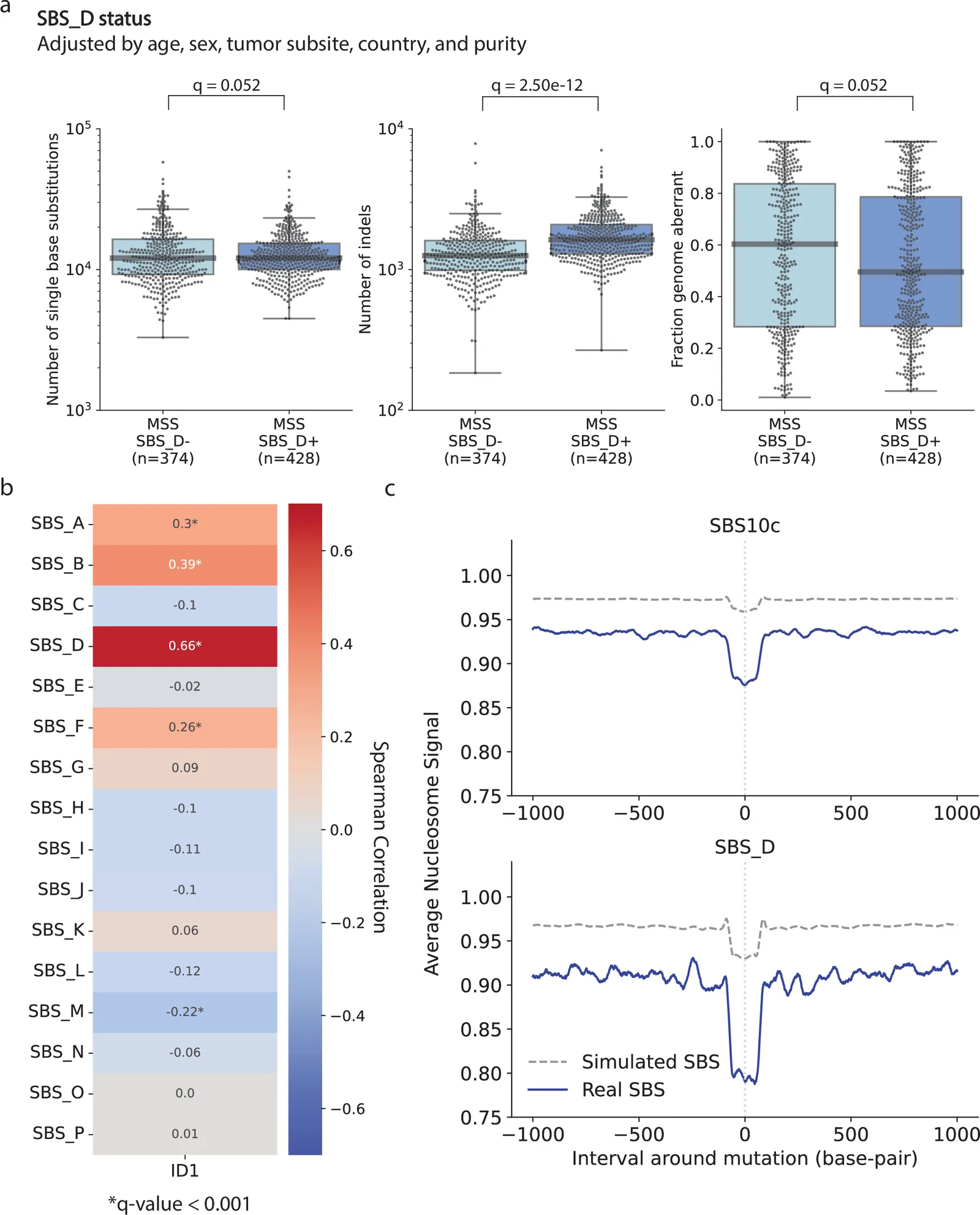
SBS_D exhibits mutational features suggestive of DNA repair infidelity. **a** Comparison of SBS_D-positive (SBS_D + ) and SBS_D-negative (SBS_D−) microsatellite stable (MSS) samples across total number of single base substitution mutations (left), total number of indel mutations (middle), and fraction of the genome with copy number aberrations (right) in the Mutographs colorectal cancer cohort. SBS_D+ samples were defined as those with non-zero SBS_D activity. *P* values were calculated using one linear model per comparison, adjusted for age, sex, tumor subsite, country, and tumor purity, followed by Benjamini-Hochberg multiple testing correction across the three comparisons to yield q-values. Box plots show the median (center line) and the 25th−75th percentiles (box bounds); whiskers extend to the most extreme data points within 1.5 times the interquartile range. All data points are shown as dots, including outliers. **b** Spearman correlation between the activity of each de novo SBS signature and ID1 activity. Asterisks indicate significant correlations with q-value < 0.001 after Benjamini–Hochberg multiple testing correction. **c** Nucleosome occupancy pattern as identified by SigProfilerTopography for SBS10c in the three *POLD1*-mutant samples from the Mutographs colorectal cancer cohort (*top*); nucleosome occupancy for SBS_D in the microsatellite-stable Mutographs colorectal cancers (bottom). Each plot is centered on the mutation site, with 1000 base pairs upstream (5’) and downstream (3’). Solid lines represent the average nucleosome signal for observed single-base substitution (SBS) mutations; dashed lines show the signal for simulated mutations. Source data are provided as a Source Data file.

Although SBS_D does not appear to result from canonical defects in *POLD1* or mismatch repair genes, the similarity in mutational patterns suggests that POLD1—and possibly mismatch repair—may still contribute to its underlying mutational processes. This hypothesis is further supported by four key observations in the Mutographs CRC cohort. First, although SBS_D-positive samples, defined as those with non-zero SBS_D activity, lacked the characteristic features of POLD1 or DNA repair deficiency—such as hypermutation and low genome aberration (Fig. 4b)—they exhibited significantly higher indel burden compared to SBS_D-negative samples (1.3-fold increase; *q* value of 2.50 × 10^−12^ after adjusting for known covariates), whereas negligible differences were observed for SBS burden and genome aberration (Fig. 5a). A markedly higher indel burden was also observed in SBS_D-positive samples across the TCGA, Swedish, and GEL cohorts (*q* values < 10^−10^; 1.3-fold increase in TCGA; 1.3-fold increase in the Swedish cohort; 1.4-fold increase in GEL; Supplementary Fig. 13). Second, a prior study^23^ showed that POLD1-deficient tumors are hypermutated specifically for indel signature ID1, which arises from replication slippage of the synthesized strand. Notably, SBS_D was the SBS signature most strongly correlated with ID1 activity (CORR = 0.66; *q* value < 0.0001; Fig. 5b). Third, SBS10c in POLD1-defective tumors exhibits a distinct nucleosome occupancy pattern, marked by strong depletion at nucleosome-bound regions, and a similar depletion pattern was observed for SBS_D in the DNA repair-proficient samples (Fig. 5c). Fourth, using the recently published alternative framework for indel signatures^28^, which identified indel signatures associated with DNA repair deficiency, we observed that InD9b (no similar COSMIC signature and no proposed etiology) was negatively associated with SBS_D activity (Supplementary Fig. 14a), whereas InD1 and InD2a (similar to COSMIC ID1 and ID2, respectively, which are elevated in SBS_D-positive samples in the Mutographs CRC cohort; Supplementary Fig. 14b), and InD11 (no matching COSMIC signature) were positively associated with SBS_D (Supplementary Fig. 14a). InD11 was previously found to be enriched in samples with high indel burdens, such as those with DNA repair deficiencies^28^, further suggesting a relationship between SBS_D and DNA repair. Of note, DNA repair capacity is known to decline with age^29,30^, and our prior observation of a modest positive association between SBS_D activity and age at diagnosis may further reflect a connection between SBS_D and DNA repair (Supplementary Figs. 6 and 7). While we can be confident that SBS_D is not driven by established genomic aberrations in *POLD1* and mismatch repair genes, our results suggest that there may be a link between SBS_D and infidelity in these DNA repair processes.

### COSMIC classification and etiology of SBS_D

The consistent identification of SBS_D across multiple independent cohorts, together with its distinct mutational profile and orthogonal biological features, supports its classification as a novel mutational signature within the COSMIC reference framework. While naïve decomposition suggests similarity to signatures associated with POLD1 proofreading deficiency and mismatch repair deficiency, the absence of genomic or phenotypic evidence of these defects indicates that SBS_D does not arise from canonical DNA repair deficiency. Accordingly, SBS_D is classified as a mutational signature of unknown etiology under COSMIC conventions. SBS_D has been submitted to the COSMIC database and is provisionally designated SBS111. In the COSMIC annotation, it is noted that, although SBS_D does not reflect canonical DNA repair deficiency, its genomic features are consistent with a hypothesis of infidelity in these DNA repair processes.

## Discussion

In this study, we revisited the Mutographs CRC mutational signature analysis^9^, where mutational signature SBS_D was previously decomposed into the reactive oxygen species-associated signature SBS18. Using molecular timing and topography analysis, which has previously been used to distinguish between two mutational processes that present as similar mutational signatures^22^, we demonstrated that SBS_D was a late clonal signature enriched in linker-DNA, which is in direct contrast to the molecularly early/consistent and enriched at nucleosomes behavior observed for SBS18. SBS_D (provisionally designated SBS111 in COSMIC) therefore represents a reproducible mutational signature distinct from currently cataloged signatures. Although our results do not alter the main finding from the Mutographs CRC project, they do indicate that the conservative approach taken by Mutographs has missed that SBS_D represents a distinct mutational process from SBS18. Furthermore, although SBS_D is consistently present at similar prevalence across multiple independent microsatellite-stable CRC cohorts, it was not previously reported in these studies.

As multiple large-scale colorectal cancer cohorts^14,15^ have previously undergone mutational signature analysis, it is surprising that SBS_D has not been reported. A likely explanation is that these studies did not separate DNA repair-deficient and DNA repair-proficient cancers prior to performing de novo signature extraction. DNA repair-deficient tumors typically harbor an order of magnitude more SBSs—~10^5^ compared to about 10^4^ in microsatellite-stable cases—and are dominated by canonical deficiency signatures such as SBS10c and SBS15. Because SBS_D shares certain features with these repair-deficiency signatures, its signal is masked when hypermutated tumors are analyzed together with repair-proficient cases. This challenge is compounded by the fact that SBS_D usually contributes between 5 and 20% of mutations in SBS_D-positive microsatellite-stable tumors, making it a relatively weak process in mixed cohorts. Thus, when extraction algorithms attempt to resolve the much stronger signals of SBS10c and SBS15, SBS_D is not recovered as a distinct signature.

To address this, the original Mutographs CRC study^9^ implemented a key strategy by analyzing microsatellite-stable and unstable samples separately. However, the study also adopted a conservative stance and did not classify SBS_D as a novel signature. At the time, its decomposition into known reference signatures was borderline, and without the additional evidence presented in this study, SBS_D was interpreted as a composite rather than a distinct entity. The results in this study underscore the need to further investigate signatures with borderline decompositions, including analyses of additional cohorts and evaluation of naive signature decomposition approaches that incorporate all known reference signatures.

Overall, despite extensive studies on CRC mutational landscapes, our findings reveal a previously unrecognized mutational signature in DNA repair-proficient CRC. Although RNA expression analyses and associations with clinicopathological features did not yield conclusive insights into SBS_D etiology, our genomic-level analyses suggest that SBS_D may reflect DNA repair infidelity during the late stages of CRC evolution. While SBS_D can be mathematically decomposed into signatures associated with DNA repair deficiency, such as SBS10c and SBS15, our SBS_D-positive samples show no genomic or phenotypic evidence of DNA repair deficiency. Although these tumors display features suggestive of mild repair infidelity, such as elevated indel burdens compared to SBS_D-negative samples, they lack the characteristic phenotypes of repair-deficient tumors and pathogenic mutations in known DNA repair genes. The consistency of SBS_D across multiple CRC cohorts and the lack of evidence of DNA repair-deficiency in SBS_D-positive samples support its classification as a distinct signature, potentially associated with DNA repair infidelity, rather than a composite of known repair-deficiency signatures. However, as the field of mutational signatures is ever-evolving with the generation of larger whole-genome sequenced cohorts, we acknowledge that we cannot exclude the possibility that SBS_D will be separated out into smaller components or otherwise modified as more data become available. Despite this, our evidence suggests that the signal observed through SBS_D likely represents a mutational process previously thought to be absent from DNA repair-proficient CRC samples. To conclusively determine the etiology of SBS_D, further in vitro and in vivo experiments will be required. Notably, recent advances in polymerase error rate sequencing (PER-seq) have provided further insights into polymerase epsilon fidelity^31^, and similar approaches will be needed to experimentally validate the potential role of POLD1 infidelity in SBS_D.

## Methods

### Mutational contexts

For somatic SBSs, the SBS-96 context is the most widely used classification schema and serves as the foundation for the current set of COSMIC reference SBS signatures^5^. Briefly, the SBS-96 mutational context organizes somatic mutations into six base substitution types: C > A, C > G, C > T, T > A, T > C, and T > G (according to the pyrimidine base of the Watson-Crick base-pair). Each type is divided into 16 subtypes based on the trinucleotide context, which accounts for the bases immediately preceding and following the mutation (4 possible bases at the 5′ position × 4 at the 3′ position × 6 substitution types = 96 categories)^32^.

While SBS-96 is the most used schema for SBSs, higher-resolution schemas like SBS-288 and SBS-1536 have been applied in recent large-scale analyses, including in previous Mutographs studies^9–11,33^, due to their abilities to enhance resolution by incorporating additional features. The SBS-1536 classification extends the SBS-96 schema by incorporating the pentanucleotide context, resulting in 1536 mutational channels (256 per each of the six base substitution types). The SBS-288 classification builds upon SBS-96 by introducing three additional subcategories for each mutation: (i) non-transcribed, (ii) transcribed, and (iii) un-transcribed. This schema differentiates mutations occurring in non-transcribed (i.e., non-genic) regions of the genome from those in transcribed regions of the genome. Mutations within transcribed regions are further divided based on the transcriptional strand orientation relative to the pyrimidine mutated base^32^, resulting in the transcribed and un-transcribed classifications. Both the SBS-288 and SBS-1536 schemas are fully collapsible to SBS-96, allowing extracted signatures to be converted back to the SBS-96 schema for direct comparison with COSMIC reference signatures. The mutational context enables the transformation of mutational catalogs from cancer genomes into a mutational matrix, where each row represents a specific mutation type, and each column corresponds to a tumor sample.

### Mutational signature extraction and decomposition using SigProfiler

Mutational signature extraction and decomposition results from the Mutographs CRC Project^9^ were utilized in this study. Briefly, we previously used SigProfilerExtractor^8^, which involves performing nonnegative matrix factorization on a mutational matrix, to identify the underlying mutational signatures in the cohort. Extractions were performed using the SBS-96, SBS-288, and SBS-1536 contexts, and the signatures from the SBS-288 extraction were decomposed and used for the Mutographs CRC study. Decomposition of the de novo signatures, which means identifying which known reference signatures best explain each of the de novo signatures, was performed using an optimized approach where the following signature subsets were removed from the decomposition: artifact signatures, UV signatures, lymphoid signatures, mismatch repair deficiency signatures, polymerase deficiency signatures, base excision repair deficiency signatures, and treatment signatures. This was done using SigProfilerAssignment^7^ and utilizing the exclude_signature_subgroups parameter.

### Mutational signatures extraction using MuSiCal and SignatureToolsLib

Mutational signature extraction using MuSiCal^17^ (v1.0.0) and SignatureToolsLib^18^ (v2.4.6) was conducted on the Mutographs CRC cohort. Extraction with MuSiCal was performed using the SBS-288 matrix, run for 1–20 components, and the selected solution (*k* = 16) was utilized. Extraction with SignatureToolsLib was performed using the SBS-96 matrix, run for 2–20 components, and utilized the previously described parameters^18^. Because SignatureToolsLib does not select an optimal solution, we selected the largest *k* with average silhouette width ≥0.80.

### Identification of mutational signature activity in each sample

As in the original Mutographs CRC project^9^, mutational signature attribution^34^ (MSA) was used to attribute each of the de novo mutational signatures to each sample. MSA was run using the same parameters used for the original Mutographs CRC study, including setting the no_CI_for_penalties parameter to false for penalty selection. Analyses were performed using the pruned attributions produced by the tool. Somatic mutations attributed to each mutational signature after mapping to COSMICv3.4—along with SBS_D, which is now classified as a novel signature—are provided in Supplementary Data 25.

### Molecular timing of SBS18^ and SBS_D

Copy number profiles, generated using Battenberg^35^, and somatic variant calls, generated using a consensus between the CaVEMan^36^ and Strelka2^37^ callers, were generated as part of the Mutographs CRC project. These data were used to run DPClust3p^35^ and DPClust^35^ to identify the cancer cell fraction (CCF) of each somatic mutation and the estimated number of chromosomes carrying each somatic mutation. The following logic was applied: in areas with copy number amplifications, if a mutation appeared on more than one chromosome copy, it was labeled as early clonal, and if a mutation appeared on one or less chromosome copies when a gain had occurred on the major allele in that location, it was labeled as likely late clonal; this logic was only applied to clonal mutations (those with CCF > 0.95).

Samples with at least 256 early clonal and 256 late clonal mutations were retained for analysis (684/774), as has been done in previous studies^10,11^. MSA was then used to identify the activity of each de novo mutational signature in each early clonal and late clonal set of mutations. A McNemar test was used to assess if the frequencies of samples with SBS_D activity and SBS18^ activity were different between late clonal and early clonal mutations.

### Topography analysis

SigProfilerTopography^38^ was used to analyze the topographical features of SBS_D, SBS18^, and SBS10c. Probability files and activity files were generated using MSA as part of the Mutographs CRC project. For the SBS_D and SBS18^ analysis, MSA was run with the SBS-288 de novo signatures and the 802 DNA repair-proficient samples. For the SBS10c analysis, MSA was run using the COSMICv3.4 reference signatures and the three *POLD1*-mutant samples from the full Mutographs CRC cohort. Both runs of SigProfilerTopography^38^ used 0.75 for the average_probability parameter. For replication timing and replication strand asymmetry analyses, the HCT116 colon carcinoma cell line was used as the reference. For nucleosome occupancy, the K562 cell line was used as the reference.

### Somatic variant calling in three independent cohorts

For the Swedish Cohort, somatic mutation calls were publicly available and had been identified using Mutect^39^, Mutect2^40^, and TNscope^41^. For GEL, only the SBS-96 mutational matrix was available; somatic mutations for GEL were identified using Strelka^37^. Because mutation calls are not yet available for the TCGA whole-genome sequenced CRC samples, we identified mutations using Mutect2^40^ with the pcr-indel-model parameter set to HOSTILE and all other parameters set to default values; the standard PASS filter was applied.

### Sample filtering in three independent cohorts

Samples in each independent cohort were filtered to mimic the DNA repair-proficient sample set used in the Mutographs CRC project. For all three cohorts, samples with over 100,000 SBS mutations or over 7000 indel mutations were removed. In addition, any samples with <1000 SBS mutations were removed. To account for mutations in *MUTYH*, *NTLH1*, and *OGG1*, samples with cosine similarity >0.80 to any of the respective signatures (SBS36 from COSMIC^42^, SBS30 from COSMIC^42^, and SBS108 from SIGNAL^19^) were removed. Like in the Mutographs CRC project, two samples from the Swedish cohort were removed for suspected microsatellite instable activity (high levels of SBS15) despite not being hypermutators.

### Mutational signature extraction in three independent cohorts

After filtering, SBS matrices were generated for the TCGA and Swedish cohort using SigProfilerMatrixGenerator^32^. For the GEL cohort, the publicly available SBS-96 mutational matrix was filtered and used for downstream analysis, as the variant calling files are not publicly available. SigProfilerExtractor^8^ with default parameters was then used to extract mutational signatures from each cohort, and the suggested solution was used for each extraction.

### Characterizing pathogenicity of Mutations in *POLD1* and DNA mismatch repair genes

Ensembl variant effect predictor^43^ was previously used to annotate mutations in the Mutographs CRC cohort. All mutations with the MODIFIER annotation (lowest impact) were filtered out. All protein-altering SBS and indel mutations from the 802 DNA repair-proficient samples in *POLD1* and DNA mismatch repair genes (*MLH1*, *MLH3*, *MSH2*, *MSH3*, *MSH6*, *PMS1*, and *PMS2*) were examined using CLINVAR^44^. Results were reported in Supplementary Data 23 (single base substitutions) and Supplementary Data 24 (indels).

### Linear models for comparing genomic features

Generalized linear models (Python version 3.9.7; statsmodels version 0.13.2) were used to identify the association between SBS_D status and total number of single base substitutions, total number of indel mutations, fraction genome aberrant, number of ID1 mutations, and number of ID2 mutations. Fraction genome aberrant values came from the original Mutographs CRC study^9^. A single model was used for each feature, and sex, age, tumor subsite, country, and purity were all included as covariates. This same approach was used to identify associations between SBS_D status and total number of indel mutations in the Swedish and TCGA cohorts^13,14^. No linear modeling was performed for the GEL cohort^15^ due to the lack of publicly available metadata.

### Linear models for clinical and pathological associations

A generalized linear model (Python version 3.9.7; statsmodels version 0.13.2) was used to identify associations between SBS_D status and clinical and pathological variables available for the Mutographs^9^, TCGA^13^, and Swedish^14^ cohorts. For the Mutographs cohort, a logistic regression model with SBS_D status as the dependent variable and age, sex, tumor subsite, purity, tumor stage, tumor grade, and country as the independent variables was utilized. For the TCGA cohort, a logistic regression model with SBS_D status as the dependent variable and age, sex, tumor subsite, purity, and tumor stage as the independent variables was utilized. For the Swedish cohort, a logistic regression model with SBS_D status as the dependent variable and age, sex, tumor subsite, purity, tumor stage, and tumor grade as the independent variables was utilized. For all cohorts, tumor stage was treated as a numeric variable (1–4). These analyses were then repeated using a linear regression model on the SBS_D+ samples, with SBS_D activity as the dependent variable and all aforementioned variables as predictors in their respective cohorts.

### Associations between driver genes and SBS_D

Generalized linear models (Python version 3.9.7; statsmodels version 0.13.2) were used to identify associations between SBS_D status and mutations in driver genes identified in the original Mutographs CRC study^9^. Only driver genes mutated in at least 20/802 samples were examined. Logistic regression models, one per driver gene, were used as follows: SBS_D as the dependent variable and age, sex, tumor subsite, country, purity, and the mutation status (mutated versus not mutated) of the driver gene as independent variables. P-values corresponding to each driver gene were then corrected across all examined genes using Benjamini-Hochberg correction. This analysis was repeated using a linear regression model on the SBS_D+ samples, with SBS_D activity as the dependent variable and age, sex, tumor subsite, country, purity, and the mutation status (mutated versus not mutated) of the driver gene as independent variables.

### Differential gene expression analysis

Differential gene expression analysis between SBS_D+ and SBS_D- samples was performed using Limma^45^ and edgeR^46^, with age, sex, purity, and tumor subsite included as covariates. This analysis was performed four different ways: all the SBS_D- samples against all the SBS_D+ samples, and all of the SBS_D- samples against the top 50%, 25%, and 10% of SBS_D+ samples ranked by SBS_D activity. Prior to performing differential expression analysis, genes with low expression were filtered out (only genes with at least 10 read counts in at least 30% of samples were retained), and data were normalized using the functions *calcNormFactors* with the “TMM” method and *voom*. Genes were considered differentially expressed using a false discovery rate of *q* < 0.05 and an absolute log2 fold-change >0.25, with the exception of the Swedish cohort using the top 50% of SBS_D+ samples, where a log2 fold-change threshold of −0.3 was used to limit the number of significantly underexpressed genes from going above 500.

### Gene set enrichment analysis

Gene set enrichment analysis was performed using the GSEA software^47,48^ with the Hallmark^49^ and Gene Ontology (GO) Biological Processes^50^ gene sets on the significantly overexpressed and underexpressed genes from the TCGA and Swedish cohorts. The top 20 gene sets were retained from each analysis.

### Indel analysis using alternative indel classification framework

SigProfilerExtractor^8^ was used to extract Indel mutational signatures using the indel classification framework^28^ developed by Koh et al., from the Mutographs CRC data. De novo signatures were decomposed into the set of reference signatures developed as part of the Koh et al. study. SigProfilerAssignment^7^ was then used to attribute the decomposed signatures to each sample in the Mutographs CRC cohort. Finally, logistic regression models, one per signature, were used to identify associations between the activity of each indel signature and SBS_D; sex, age, tumor subsite, country, and purity were included as covariates in each model.

### Reporting summary

Further information on research design is available in the Nature Portfolio Reporting Summary linked to this article.

## Supplementary information

Supplementary Information

Description of Additional Supplementary Files

Supplementary Data 1–25

Reporting Summary

Transparent Peer Review file

## Source data

Source Data

## Data Availability

Somatic mutation data and metadata for the Mutographs CRC cohort were previously published and are available at the European Genome-phenome Archive (EGA) under accession code EGAS00001003774 under restricted access, which can be acquired via the EGA website (https://ega-archive.org/access/request-data/how-to-request-data/). The mutational signature SBS_D is available as part of Supplementary Data 2. Somatic mutation data for the Swedish CRC cohort were previously published and are publicly available at the European Variation Archive under accession number PRJEB61514; metadata for the Swedish cohort are available as Supplementary Data 1 from the original publication^14^. Gene expression data for the Swedish CRC cohort were previously published and are publicly available at ArrayExpress under accession number E-MTAB-12862. Somatic mutation data for the GEL CRC cohort were previously published and are available as Supplementary Data 7 from the original publication^18^. Raw sequencing data for the TCGA CRC cohort are available at the GDC Data Portal (https://portal.gdc.cancer.gov/) under restricted access, which can be obtained via dbGaP (https://dbgap.ncbi.nlm.nih.gov/aa/wga.cgi?page=login). Metadata for the TCGA cohort are publicly available at https://portal.gdc.cancer.gov/. Gene expression data for the TCGA CRC cohort are publicly available on the GDC data portal (https://portal.gdc.cancer.gov/). The COSMIC reference mutational signatures (v.3.4) were downloaded from COSMIC (https://cancer.sanger.ac.uk/signatures/downloads/), and the Signal reference mutational signatures were obtained from Supplementary Data 21 from the original publication^18^. The SBS_D signature (provisionally designated SBS111) has been submitted to the COSMIC database. Source data are provided with this paper.

## Code availability

All computational tools used for data analysis in this study are publicly available and detailed in the Methods section. The code used to generate the figures is available at https://github.com/AlexandrovLabPublications/SBS_D_manuscript_code under a BSD-2-Clause license.
