# Supplementary material for: Identification and validation of a previously missed mutational signature in colorectal cancer
Source: Nat Commun. 2026 Aug 12;17:9671. doi: 10.1038/s41467-026-76472-w (PMC13558705; doi:10.1038/s41467-026-76472-w)
Supplement: Supplementary file 1 [file 41467_2026_76472_MOESM1_ESM.pdf]

SUPPLEMENTARY INFORMATION

Identification and Validation of a Previously Missed Mutational Signature in Colorectal Cancer

Mariya Kazachkova[1-3], Burçak Otlu[4], Marcos Díaz-Gay[2,3,5], Ammal Abbasi[2,3,6], Sarah Moody[7], Zichen Jiang[2,3,6], Sandra Perdomo[8], David C. Wedge[9], Paul Brennan[8], Michael R. Stratton[7], Ludmil B. Alexandrov[1-3,6,10*]

[1]Biomedical Sciences Graduate Program, University of California San Diego, La Jolla, CA, USA

[2]Department of Cellular and Molecular Medicine, University of California San Diego, La Jolla, CA, USA

[3]Moores Cancer Center, University of California San Diego, La Jolla, CA, USA

[4]Department of Health Informatics, Graduate School of Informatics, Middle East Technical University, Ankara, Turkey

[5]Digital Genomics Group, Cancer Genomics Program, Spanish National Cancer Research Center (CNIO), Madrid, Spain

[6]Department of Bioengineering, University of California San Diego, La Jolla, CA, USA

[7]Somatic Genomics Programme, Wellcome Sanger Institute, Cambridge, UK

[8]Genomic Epidemiology Branch, International Agency for Research on Cancer (IARC/WHO), Lyon, France

[9]Manchester Cancer Research Centre, University of Manchester, Manchester, UK

[10]Sanford Stem Cell Institute, University of California San Diego, La Jolla, CA, USA

[*]Correspondence should be addressed to L2alexandrov@health.ucsd.edu.

TABLE OF CONTENTS

SUPPLEMENTARY FIGURES

Supplementary Figure 1: SBS18^ and SBS_D are recapitulated across signature extractions from multiple contexts in the Mutographs colorectal cancer cohort. 4

Supplementary Figure 2: SBS18^ and SBS_D are extracted from the Mutographs CRC cohort using independent extraction tools 6

Supplementary Figure 3: SBS_D and SBS18^ exhibit similar patterns for some topographical features. 7

Supplementary Figure 4: SBS_D is not extracted when DNA repair proficient and deficient samples are combined. 9

Supplementary Figure 5: Proportion of SBS18^ and SBS_D Mutations in Samples Across Four CRC Cohorts. 11

Supplementary Figure 6: Associations between SBS_D activity and clinical and pathological features. 13

Supplementary Figure 7: Associations between SBS_D activity and clinicopathological features using SBS_D as a continuous measure. 15

Supplementary Figure 8: Differential gene expression analysis between SBS_D+ and SBS_D- samples. 17

Supplementary Figure 9: Pathways enriched in SBS_D+ samples based on overexpressed genes in the Swedish and TCGA cohorts. 19

Supplementary Figure 10: Pathways enriched among underexpressed genes in SBS_D+ samples in the TCGA and Swedish cohorts. 21

Supplementary Figure 11: SBS_D activity is not associated with mutations in *POLD1* or mismatch repair genes. 23

Supplementary Figure 12: Mutational signature activities in a sample with a likely pathogenic *MSH6* mutation. 25

Supplementary Figure 13: SBS_D positive samples have elevated numbers of indels in independent colorectal cancer cohorts. 27

Supplementary Figure 14: Association between SBS_D and the activity of indel mutational signatures using the alternative indel framework from Koh *et al*. **28**

Supplementary Figure 1

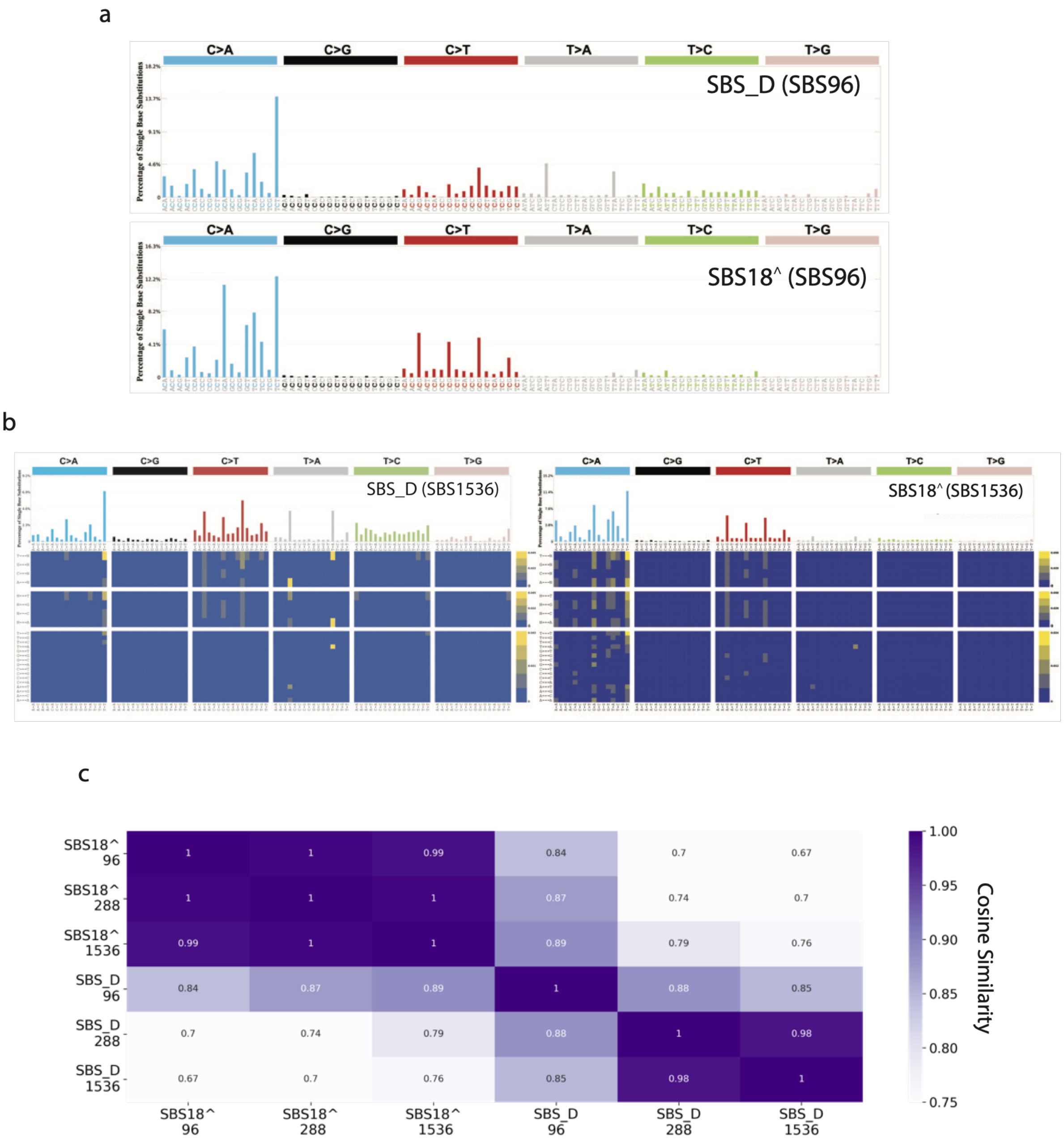

Supplementary Figure 1: SBS18^ and SBS_D are recapitulated across signature extractions from multiple contexts in the Mutographs colorectal cancer cohort. ***(a)*** SBS_D-like (*top*) and SBS18^-like (*bottom*) signatures extracted from the Mutographs colorectal cancer cohort using the SBS-

96 context. ***(b)*** SBS_D-like (*left*) and SBS18^-like (*right*) signatures extracted from the Mutographs colorectal cancer cohort using the SBS-1536 context. Heatmap represents the frequency of each context. ***(c)*** Cosine similarity between the SBS18^-like signatures and SBS_D-like signatures extracted from each of the SBS-96, SBS-288, and SBS-1536 contexts. All signatures were collapsed to the SBS-96 context for comparison. Source data are provided as a Source Data file.

Supplementary Figure 2

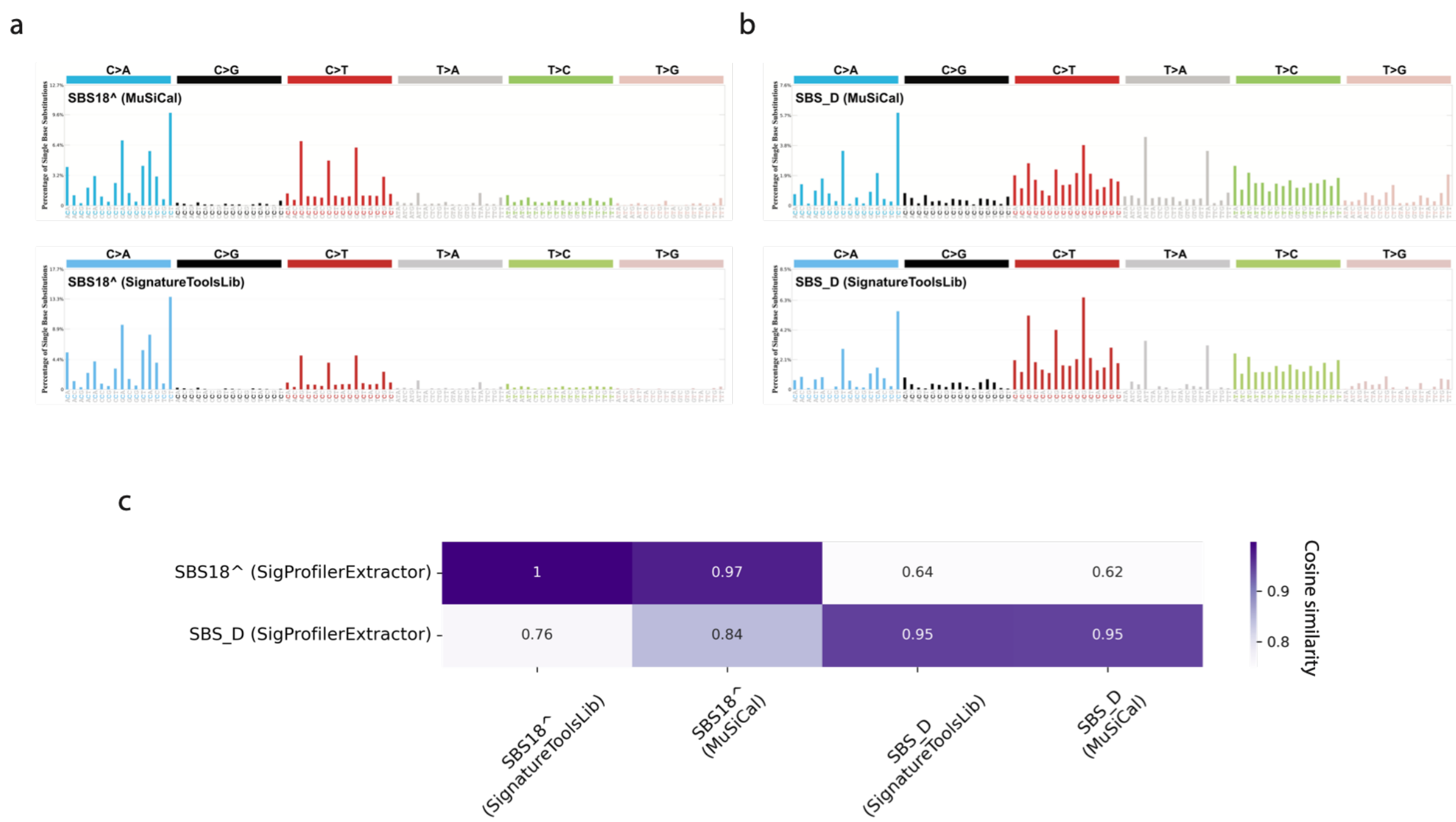

Supplementary Figure 2: SBS18^ and SBS_D are extracted from the Mutographs CRC cohort using independent extraction tools. ***(a)*** SBS18^-like signature extracted from the Mutographs CRC cohort using MuSiCal (top) and SignatureToolsLib (bottom). ***(b)*** SBS_D-like signature extracted from the Mutographs CRC cohort using MuSiCal (top) and SignatureToolsLib (bottom). ***(c)*** Cosine similarities between SBS18^ and SBS_D extracted from the Mutographs CRC cohort using SigProfilerExtractor and the SBS18^-like and SBS_D-like signatures extracted from the Mutographs CRC cohort using MuSiCal and SignatureToolsLib. Cosine similarities were calculated using the SBS-96 context for all signatures. Source data are provided as a Source Data file.

Supplementary Figure 3

a

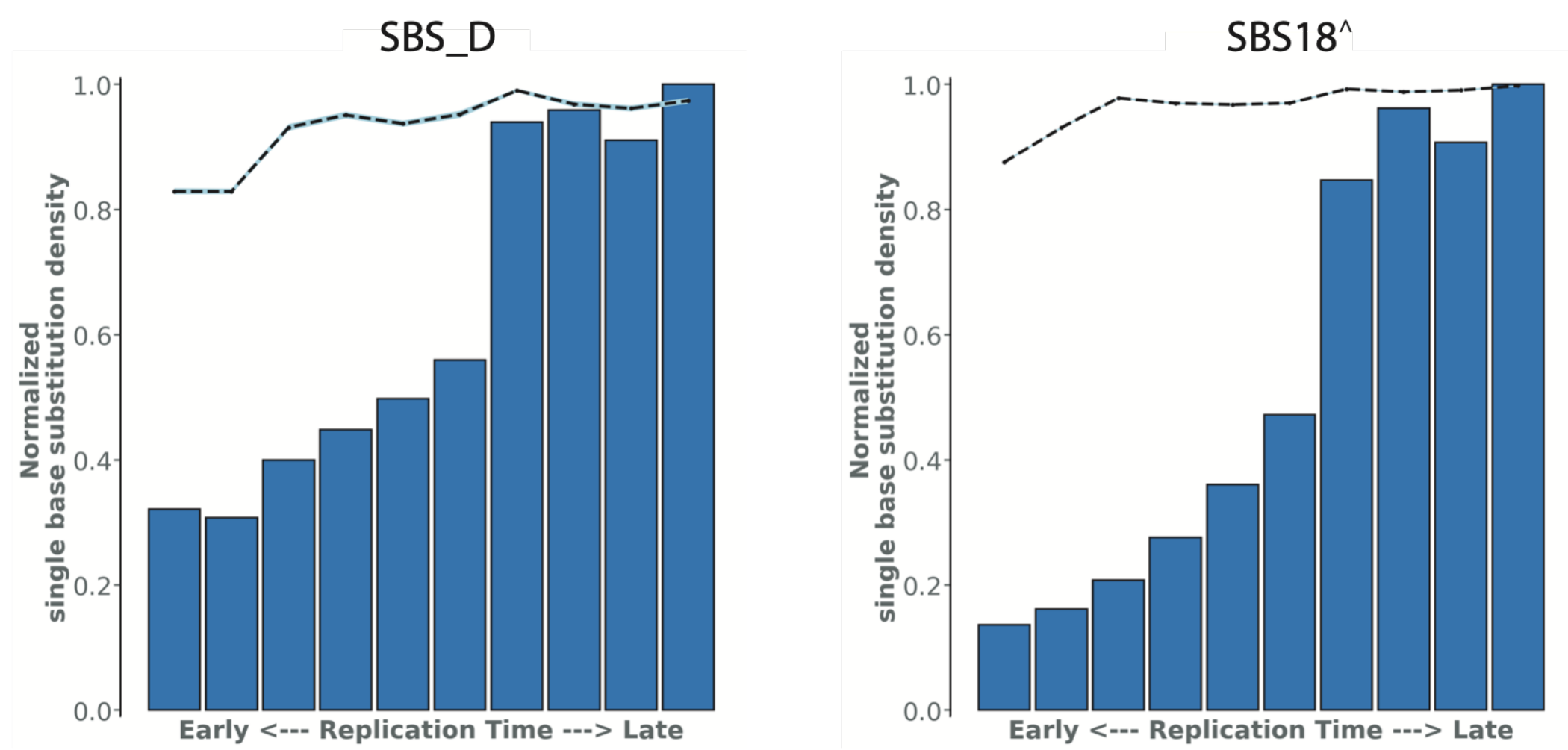

b

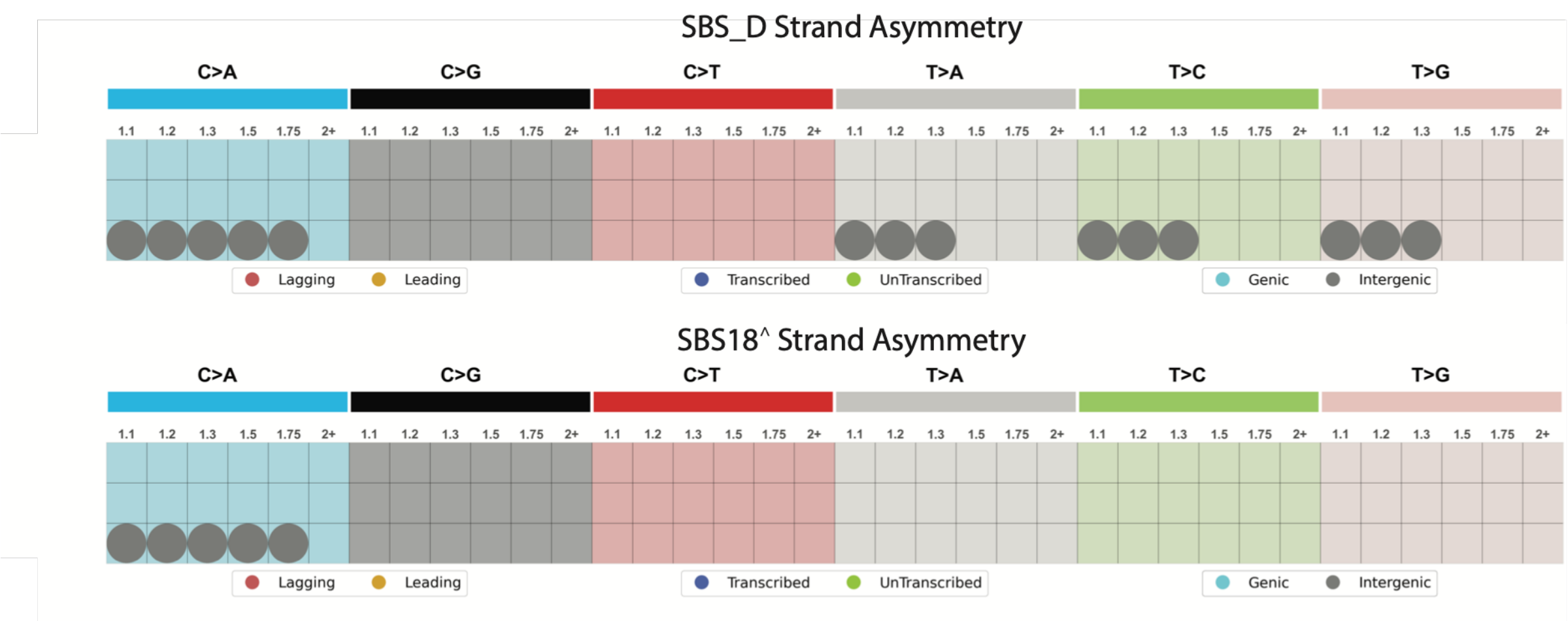

Supplementary Figure 3: SBS_D and SBS18^ exhibit similar patterns for some topographical features. ***(a)*** Replication timing patterns for SBS_D (*left*) and SBS18^ (*right*) in the Mutographs colorectal cancer cohort as identified by SigProfilerTopography. Blue bars represent deciles (10% bins) of the replication timing signal; the dashed line indicates the distribution of simulated

somatic mutations. ***(b)*** Strand asymmetry profiles for SBS_D (*top*) and SBS18^ (*bottom*) as identified by SigProfilerTopography. Circles indicate statistically significant biases (Fisher's exact test with Benjamini-Hochberg multiple testing correction), with circle color representing the genomic context (lagging versus leading, transcribed versus unTranscribed, and genic versus intergenic). Both signatures show enrichment in intergenic regions (gray circles), while no significant differences were observed between leading versus lagging or between transcribed versus un-transcribed strands. Numbers indicate odds ratios for each comparison.

Supplementary Figure 4

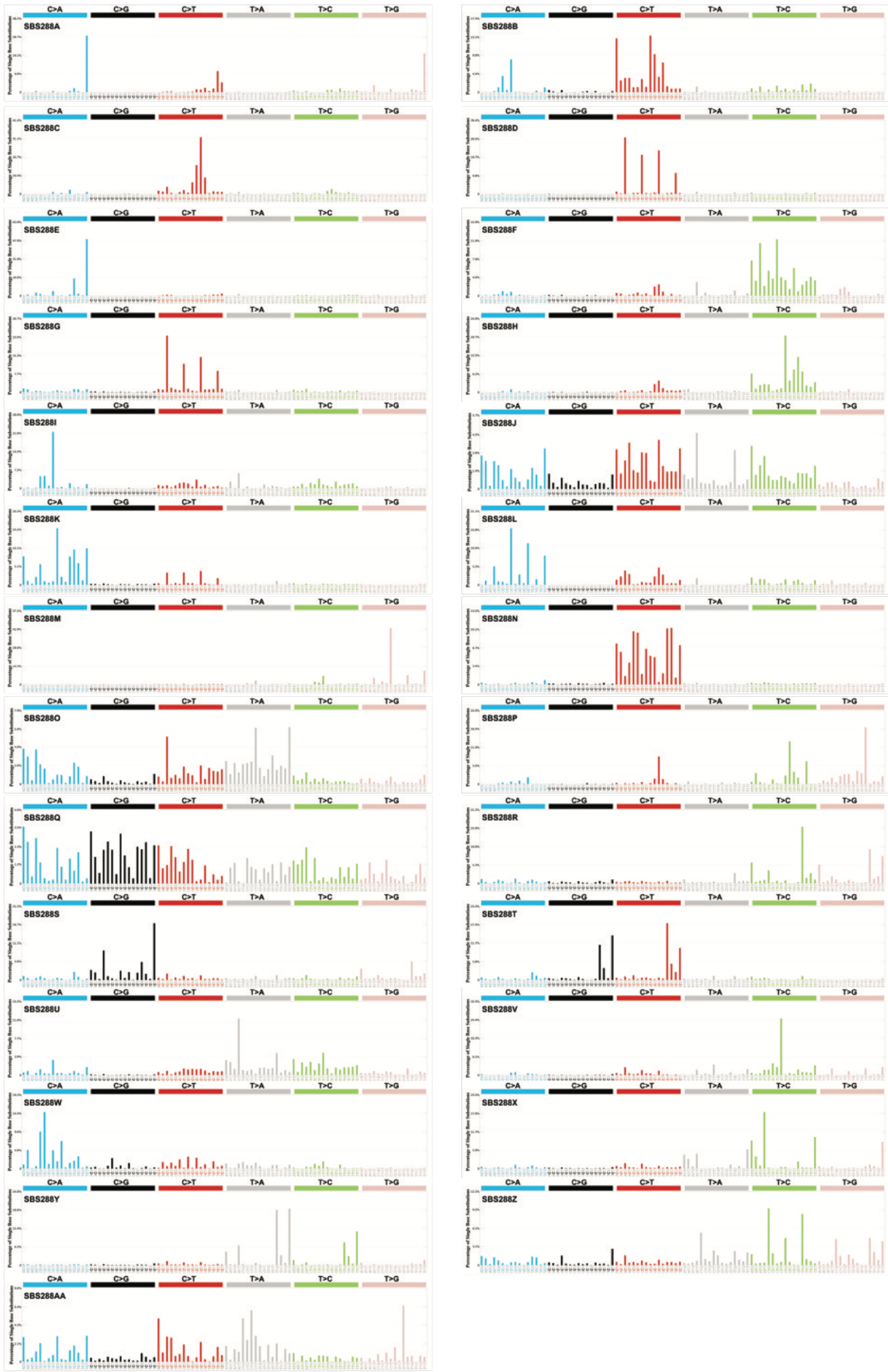

Supplementary Figure 4: SBS_D is not extracted when DNA repair proficient and deficient samples are combined. *De novo* mutational signatures extracted from all the Mutographs colorectal cancer samples (*n*=981), without stratifying by DNA repair status. Extraction was performed using SigProfilerExtractor and the SBS-288 context; for simplicity, signatures are shown here in the SBS-96 context.

Supplementary Figure 5

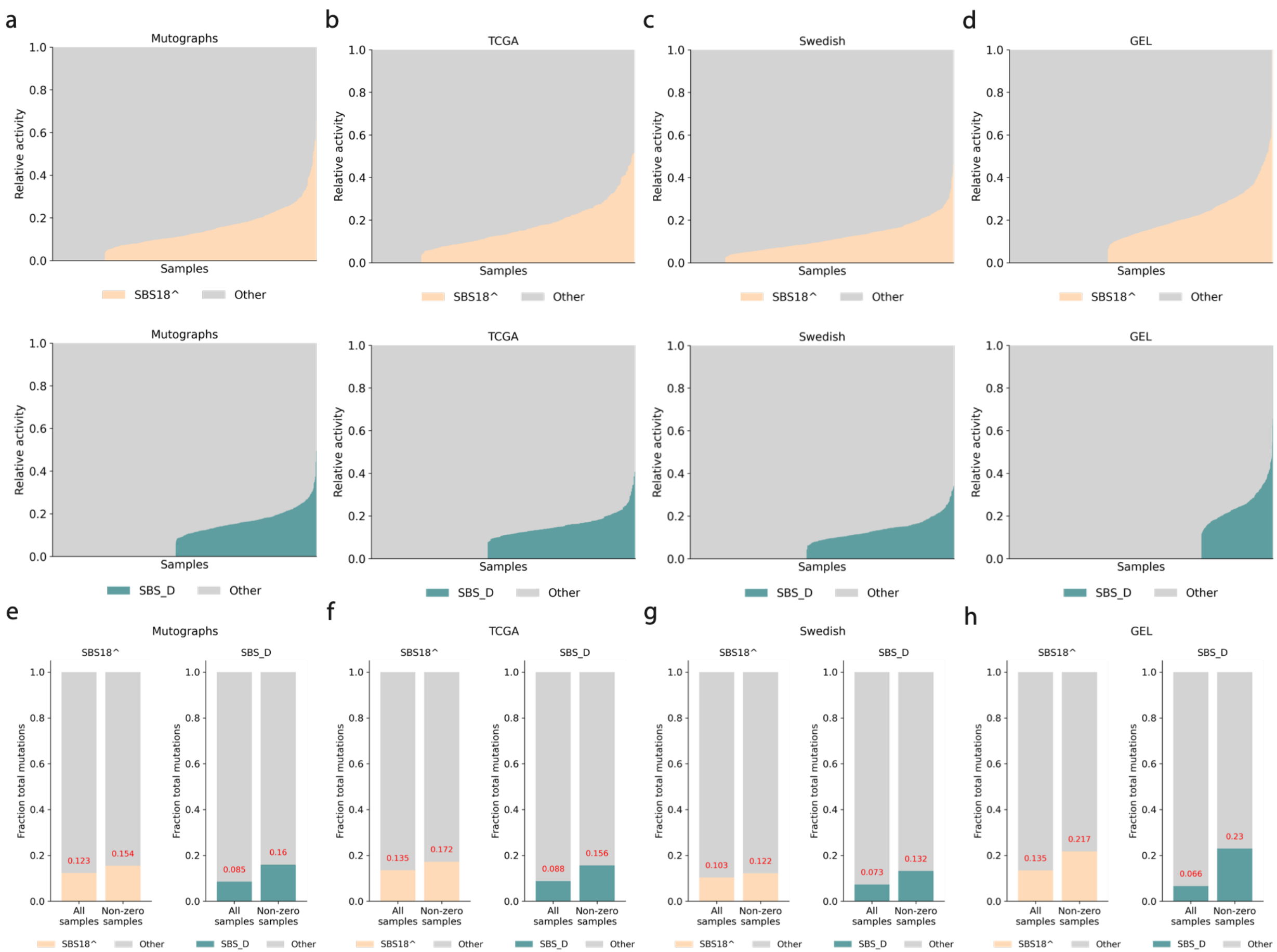

Supplementary Figure 5: Proportion of SBS18^ and SBS_D Mutations in Samples Across Four CRC Cohorts. ***(a)*** Relative activity of SBS18^ (top) and SBS_D (bottom) across samples in the Mutographs cohort (*n*=802) as identified by Mutational Signature Attribution (MSA). Samples are sorted in increasing order by the relative activity of the respective signature. ***(b)*** As in *(a)*, but for the TCGA cohort (*n*=390). ***(c)*** As in *(a)*, but for the Swedish cohort (*n*=694). ***(d)*** As in *(a)*, but for the GEL cohort (*n*=1532). ***(e)*** (left) Fraction of SBS18^ mutations out of the total mutations across all samples in the Mutographs cohort as well across only samples with non-zero SBS18^ activity in the Mutographs cohort. Red number denotes the fraction of SBS18^ mutations out of total

mutations in the respective group of samples. (right) Fraction of SBS_D mutations out of the total mutations across all samples in the Mutographs cohort as well across only samples with non-zero SBS_D activity in the Mutographs cohort. Red number denotes the fraction of SBS_D mutations out of total mutations in the respective group of samples. ***(f)*** As in *(e)*, but for the TCGA cohort. ***(g)*** As in *(e)*, but for the Swedish cohort. ***(h)*** As in *(e)*, but for the GEL cohort. Source data are provided as a Source Data file.

Supplementary Figure 6

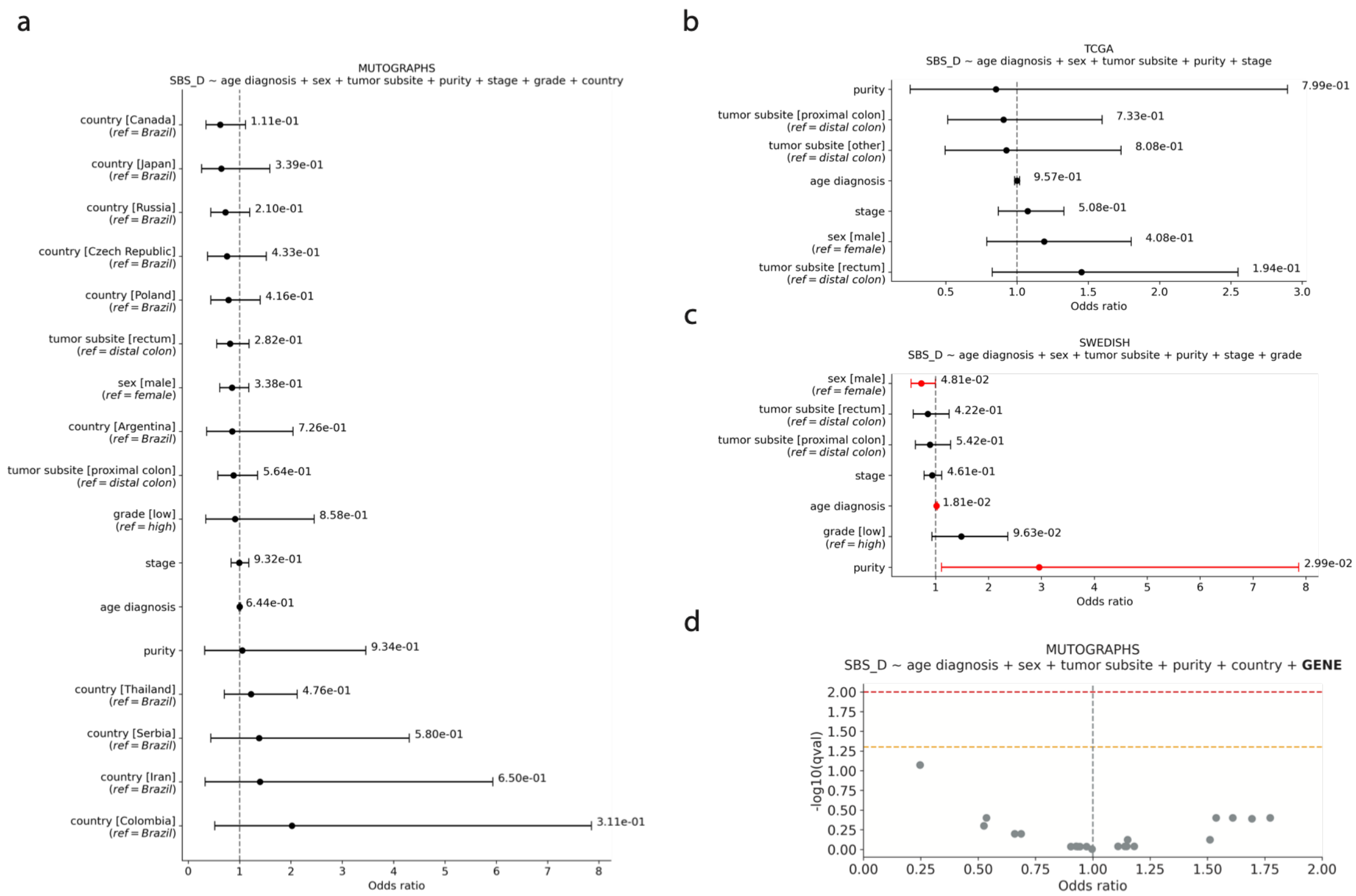

Supplementary Figure 6: Associations between SBS_D activity and clinical and pathological features. ***(a)*** Results from a logistic regression model for the Mutographs colorectal cancer (CRC) cohort with SBS_D status as the outcome variable and age of diagnosis, sex, tumor subsite, purity, stage, grade, and country included as covariates. Numbers correspond to p-values for each covariate. Whiskers for each covariate represent the 95% confidence interval. ***(b)*** As in *(a)*, but for the TCGA CRC cohort with age of diagnosis, sex, tumor subsite, purity, and stage as covariates. ***(c)*** as in *(a)*, but for the Swedish CRC cohort with age of diagnosis, sex, tumor subsite, purity, stage, and grade as covariates. Covariates with p-values < 0.05 are indicated in red. ***(d)*** Volcano plot for the Mutographs CRC cohort showing the association between each driver gene mutated in at least 20/802 samples and SBS_D. One logistic regression model was run per gene, with SBS_D as

the outcome variable and age of diagnosis, sex, tumor subsite, country, purity, and the status of the respective gene (mutated versus not mutated) as covariates. P-values for each gene were then corrected using the Benjamini-Hochberg Procedure. The orange line represents a q-value of 0.05, and the red line represents a q-value of 0.01.

Supplementary Figure 7

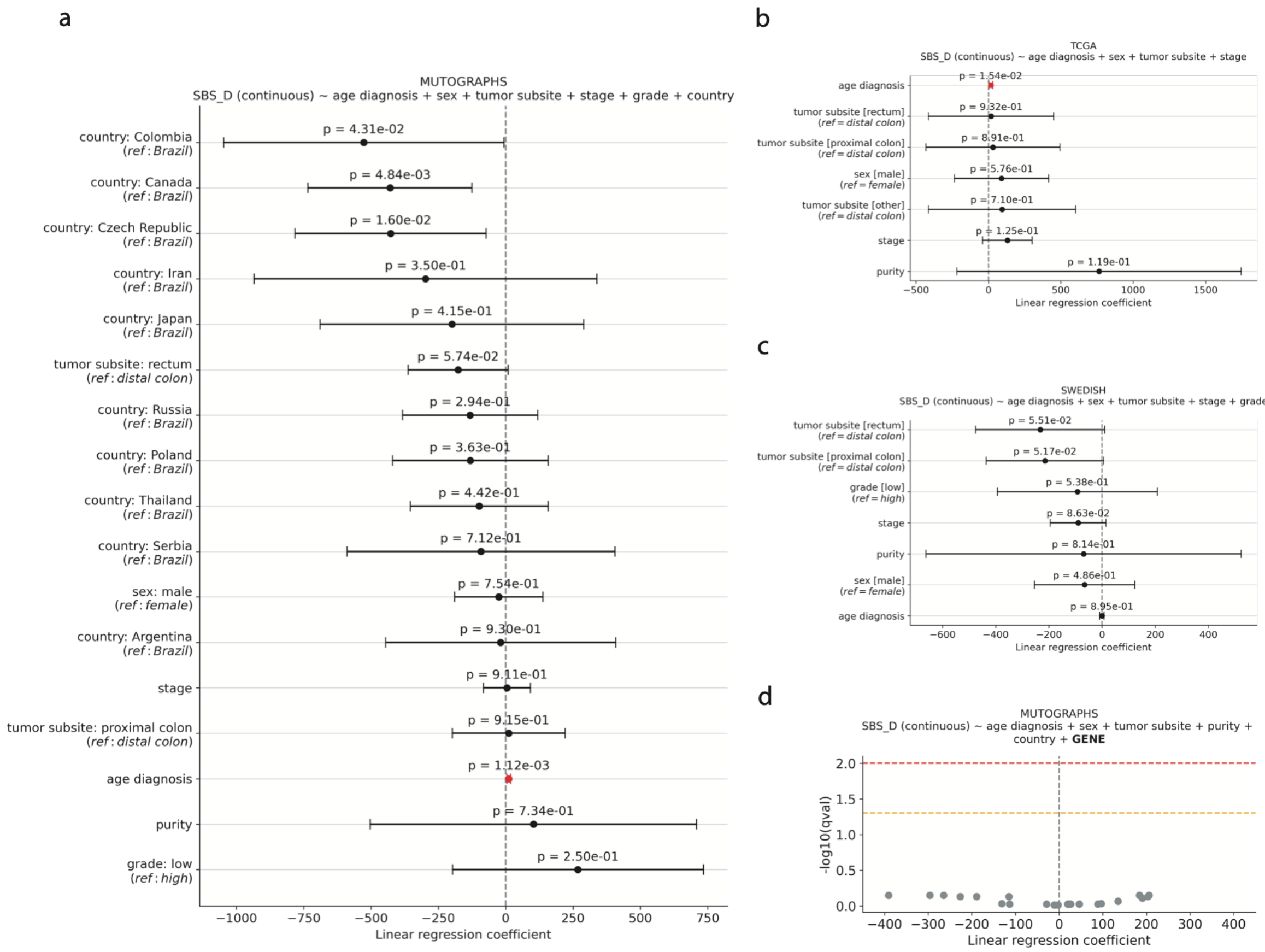

Supplementary Figure 7: Associations between SBS_D activity and clinicopathological features using SBS_D as a continuous measure. ***(a)*** Results from a linear regression model applied to SBS_D+ samples in the Mutographs colorectal cancer (CRC) cohort, with SBS_D activity as the outcome variable and age of diagnosis, sex, tumor subsite, purity, stage, grade, and country included as covariates. Points indicate effect estimates for each covariate, with whiskers representing 95% confidence intervals; corresponding p-values are shown. Whiskers for clinicopathological features (age, sex, tumor site, stage, and grade) with p-values < 0.05 are highlighted in red. ***(b)*** As in *(a)*, but for TCGA CRC cohort with age of diagnosis, sex, tumor subsite,

purity, and stage as covariates. ***(c)*** As in *(a)*, but for the Swedish CRC cohort with age of diagnosis, sex, tumor subsite, purity, stage, and grade as covariates. ***(d)*** Volcano plot for the Mutographs CRC cohort showing associations between SBS_D activity and driver gene mutations present in at least 20 of 802 samples. For each gene, a separate linear regression model was fit with SBS_D activity as the outcome variable and age at diagnosis, sex, tumor subsite, tumor purity, and mutation status of the respective gene (mutated versus not mutated) included as covariates. P-values were adjusted using the Benjamini-Hochberg procedure. The orange and red lines denote q-value thresholds of 0.05 and 0.01, respectively.

Supplementary Figure 8

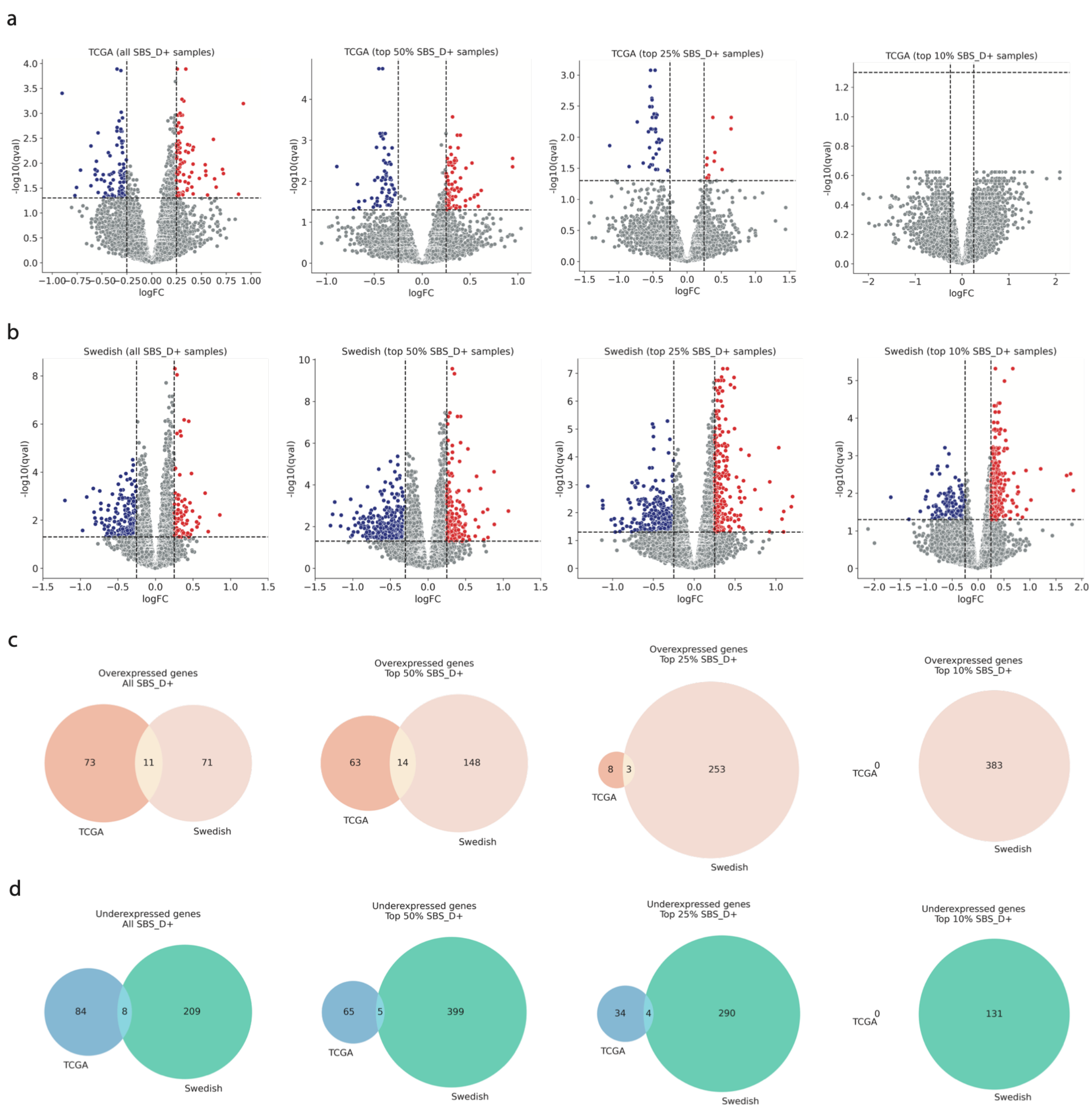

Supplementary Figure 8: Differential gene expression analysis between SBS_D+ and SBS_D- samples. ***(a)*** Volcano plots illustrating results from differential gene expression in the TCGA cohort (*n*=385 samples; 218 SBS_D+ and 167 SBS_D-). Significance was assessed using Limma; p-values

were corrected using the Benjamini-Hochberg approach. Analyses were performed using SBS_D+ samples against all SBS_D- samples, the top 50% of SBS_D+ in terms of SBS_D activity against all SBS_D- samples, the top 25% of SBS_D+ samples against all SBS_D- samples, and the top 10% of SBS_D+ samples against all SBS_D- samples; results are shown respectively, starting from the far left. Genes in blue are significantly underexpressed in SBS_D+ samples, while genes in red are significantly overexpressed in SBS_D+ samples. Dashed lines represent the thresholds used for identifying significant differentially expressed genes: a q-value of 0.05 and log2 fold-change (logFC) values of less than -0.25 and greater than 0.25 for underexpressed and overexpressed genes, respectively. ***(b)*** As in *(a)*, but for the Swedish cohort (*n*=694 samples; 388 SBS_D+ and 306 SBS_D-). ***(c)*** Venn-diagram illustrating the overlap of all significantly overexpressed genes identified across all four analyses in *(a-b)* between the TCGA and Swedish cohorts. ***(d)*** As in *(c)*, but for significantly underexpressed genes. Source data are provided as a Source Data file.

Supplementary Figure 9

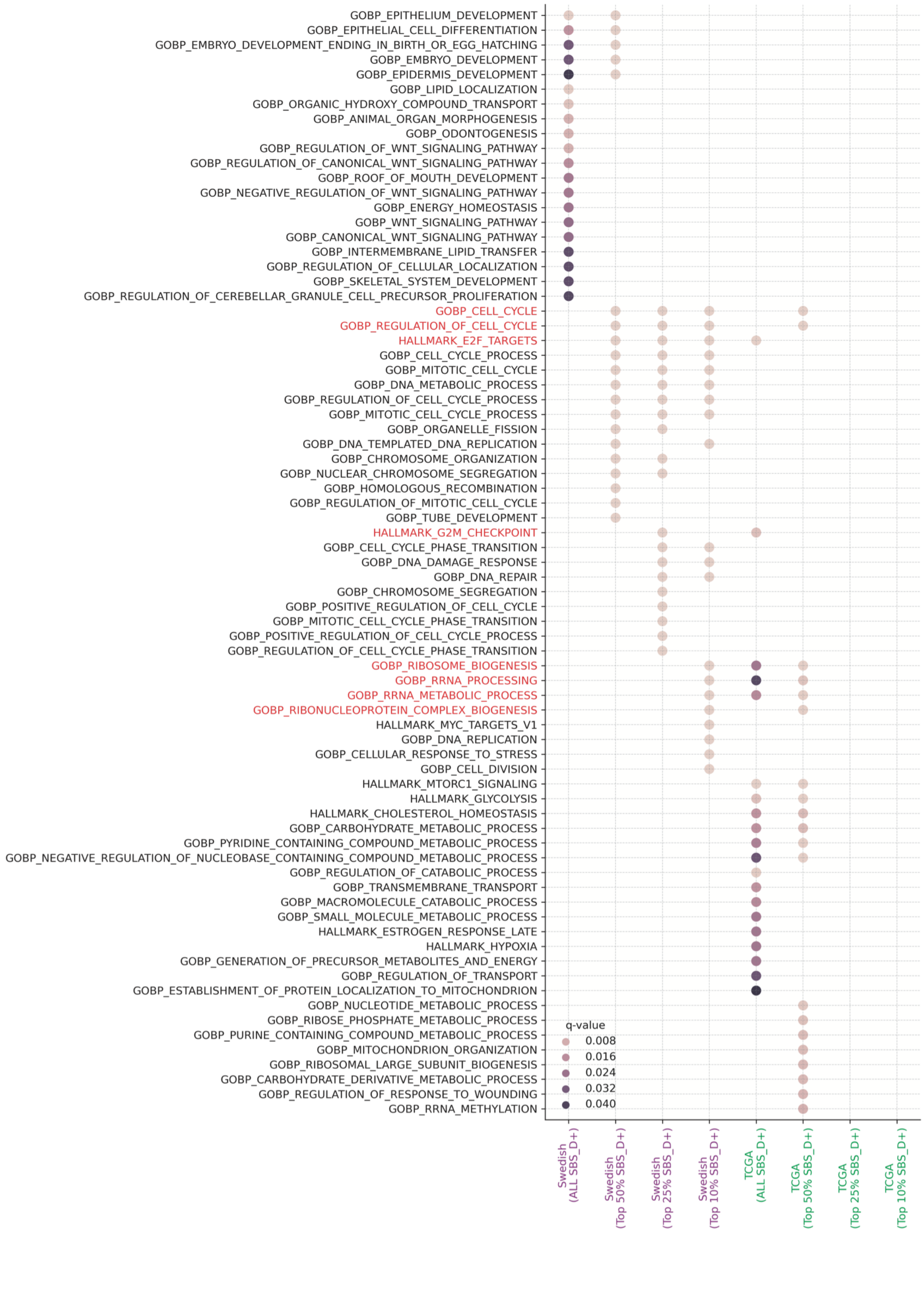

Supplementary Figure 9: Pathways enriched in SBS_D+ samples based on overexpressed genes

in the Swedish and TCGA cohorts. List of pathways significantly enriched among overexpressed genes, identified by Gene Set Enrichment Analysis (GSEA) in the Swedish (purple; x-axis) and TCGA (green; x-axis) cohorts. Analyses include comparisons of all SBS_D+ samples, as well as the top 50%, 25%, and 10% of SBS_D+ samples, against all SBS_D- samples. Hallmark and GO Biological Process gene sets were used. Each point represents a comparison in which a gene set was significantly enriched among overexpressed genes, with color indicating the corresponding q-value. Gene sets shown in red were consistently enriched in both the Swedish and TCGA cohorts in at least one analysis.

Supplementary Figure 10

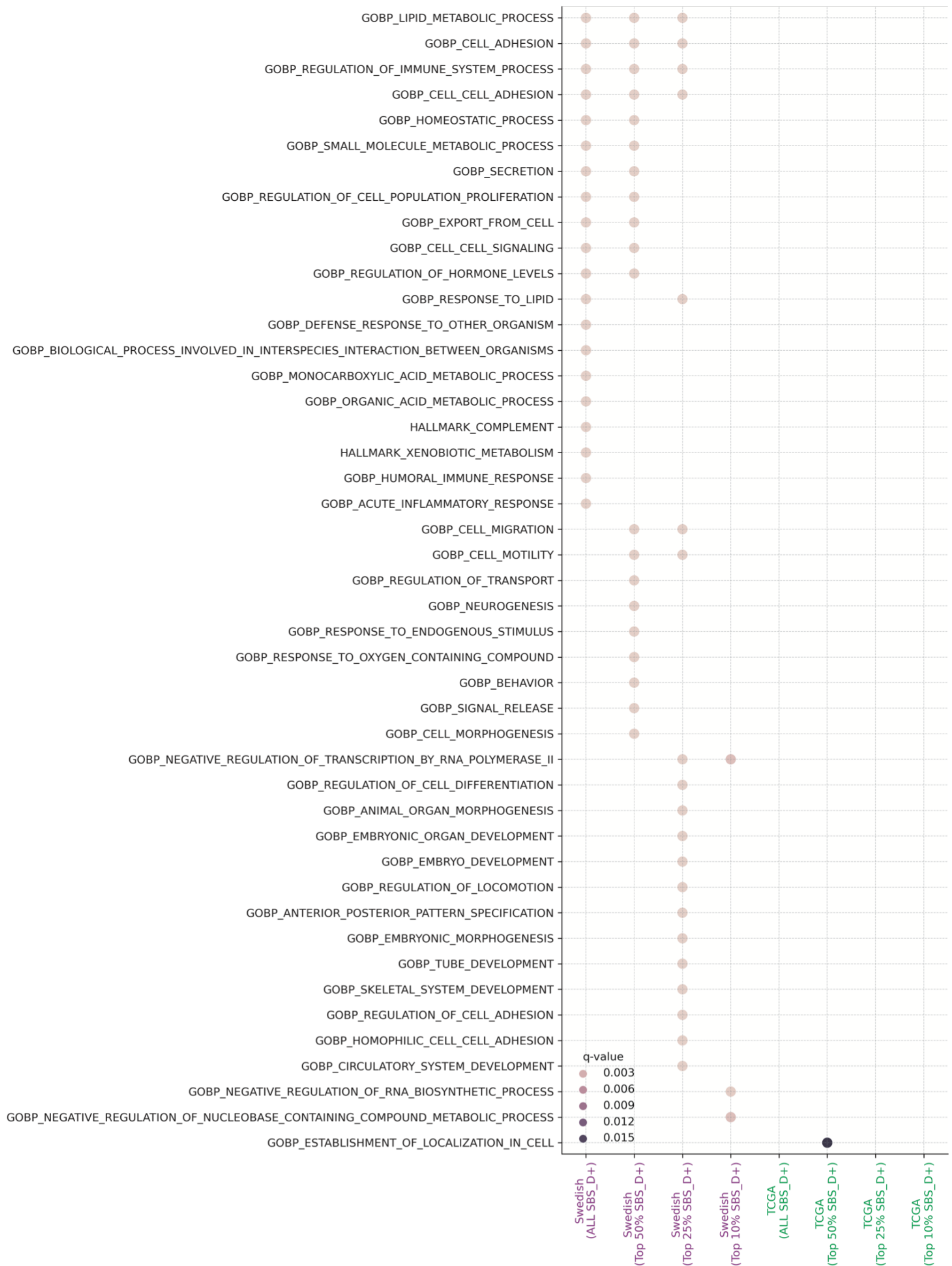

Supplementary Figure 10: Pathways enriched among underexpressed genes in SBS_D+ samples in the TCGA and Swedish cohorts. List of pathways significantly enriched among underexpressed genes, identified by Gene Set Enrichment Analysis (GSEA) in the Swedish (purple) and TCGA (green) cohorts. Analyses include comparisons of all SBS_D+ samples, as well as the top 50%, 25%, and 10% of SBS_D+ samples, against all SBS_D- samples. Hallmark and GO Biological Process gene sets were used. Each point represents a comparison in which a gene set was significantly enriched among underexpressed genes, with color indicating the corresponding q-value. No gene sets were consistently enriched among underexpressed genes across both the Swedish and TCGA cohorts.

Supplementary Figure 11

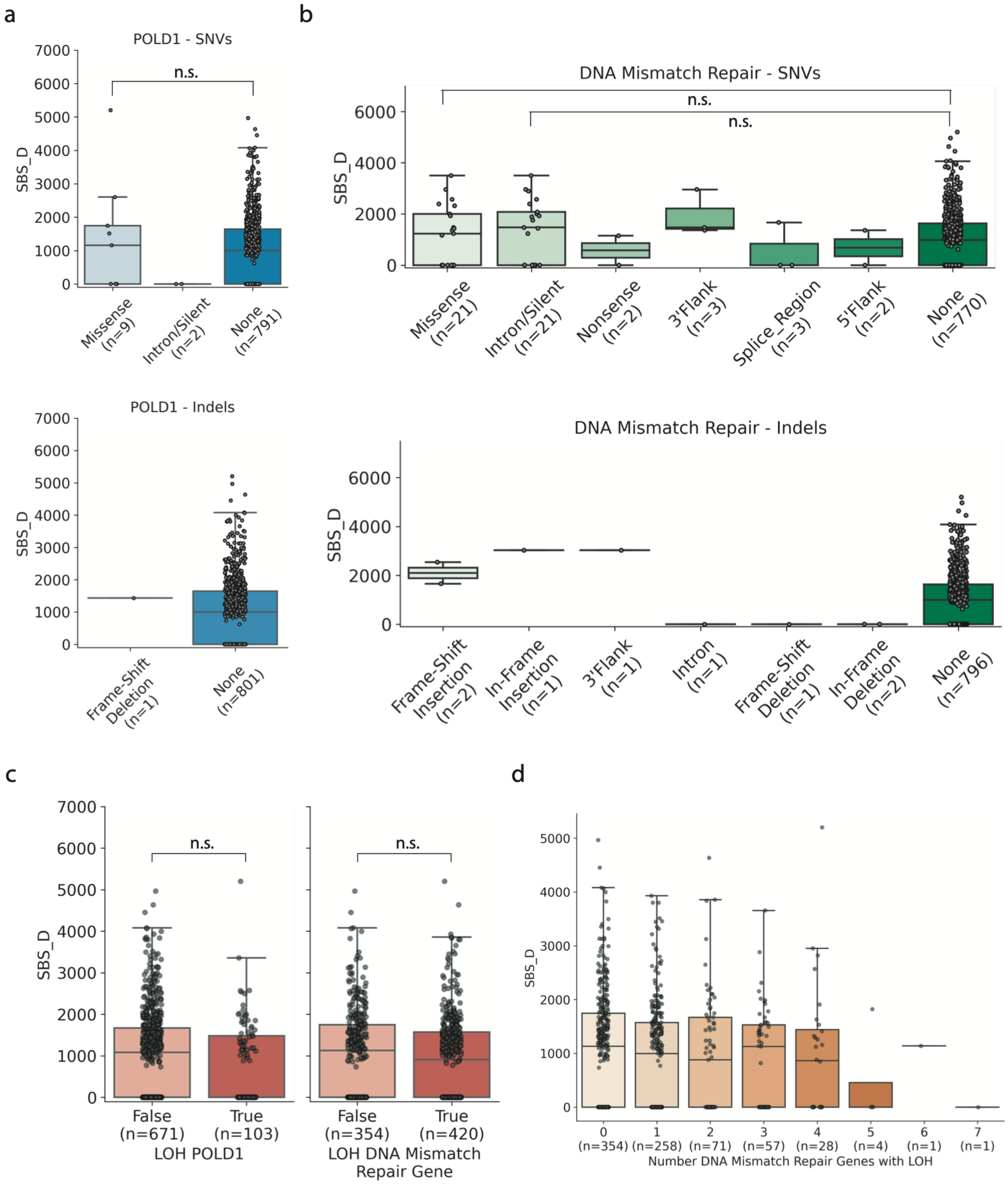

Supplementary Figure 11: SBS_D activity is not associated with mutations in *POLD1* or mismatch repair genes. ***(a)*** SBS_D activity in samples with single nucleotide variants (SNVs, *top*)

and indel mutations (*bottom*) in *POLD1* in the Mutographs colorectal cancer cohort. Mutation type was identified using the Ensembl Variant Effect Predictor (VEP), and mutations with the lowest impact (MODIFIER) were excluded from the analysis. Statistical significance was assessed using the Mann-Whitney U test for mutation categories with ≥5 samples. Number below each box indicates the number of samples with the specific mutation type. Box plots show the median (center line) and the 25th - 75th percentiles (box bounds); whiskers extend to the most extreme data points within 1.5 times the interquartile range. All data points are shown as dots, including outliers. ***(b)*** As in *(a)*, but for mutations in known DNA mismatch repair genes (*MLH1*, *MLH3*, *MSH2*, *MSH3*, *MSH6*, *PMS1* and *PMS2*). ***(c)*** Comparison of SBS_D activity in samples with and without loss of heterozygosity (LOH) in *POLD1* (*left*) and mismatch repair genes (*right*). Significance was assessed using a Mann-Whitney U test. Number below each box indicates the number of samples with LOH in the specific gene(s). Box plots show the median (center line) and the 25th - 75th percentiles (box bounds); whiskers extend to the most extreme data points within 1.5 times the interquartile range. All data points are shown as dots, including outliers. ***(d)*** SBS_D activity stratified by the number of mismatch repair genes with LOH per sample. Number below each box indicates the number of samples with LOH in the respective number of DNA mismatch repair genes with LOH. Box plots show the median (center line) and the 25th - 75th percentiles (box bounds); whiskers extend to the most extreme data points within 1.5 times the interquartile range. All data points are shown as dots, including outliers. Source data are provided as a Source Data file.

Supplementary Figure 12

a

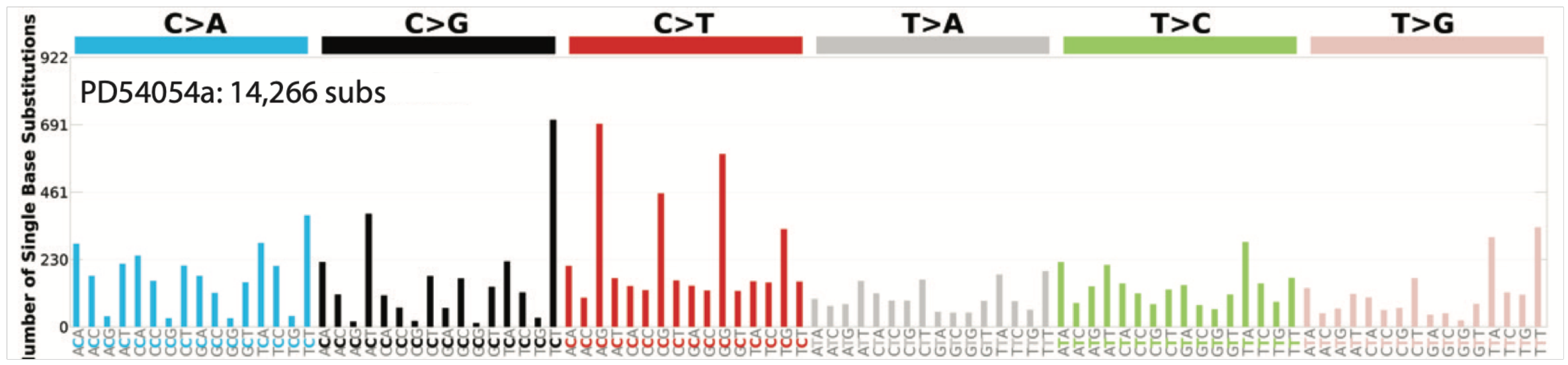

b

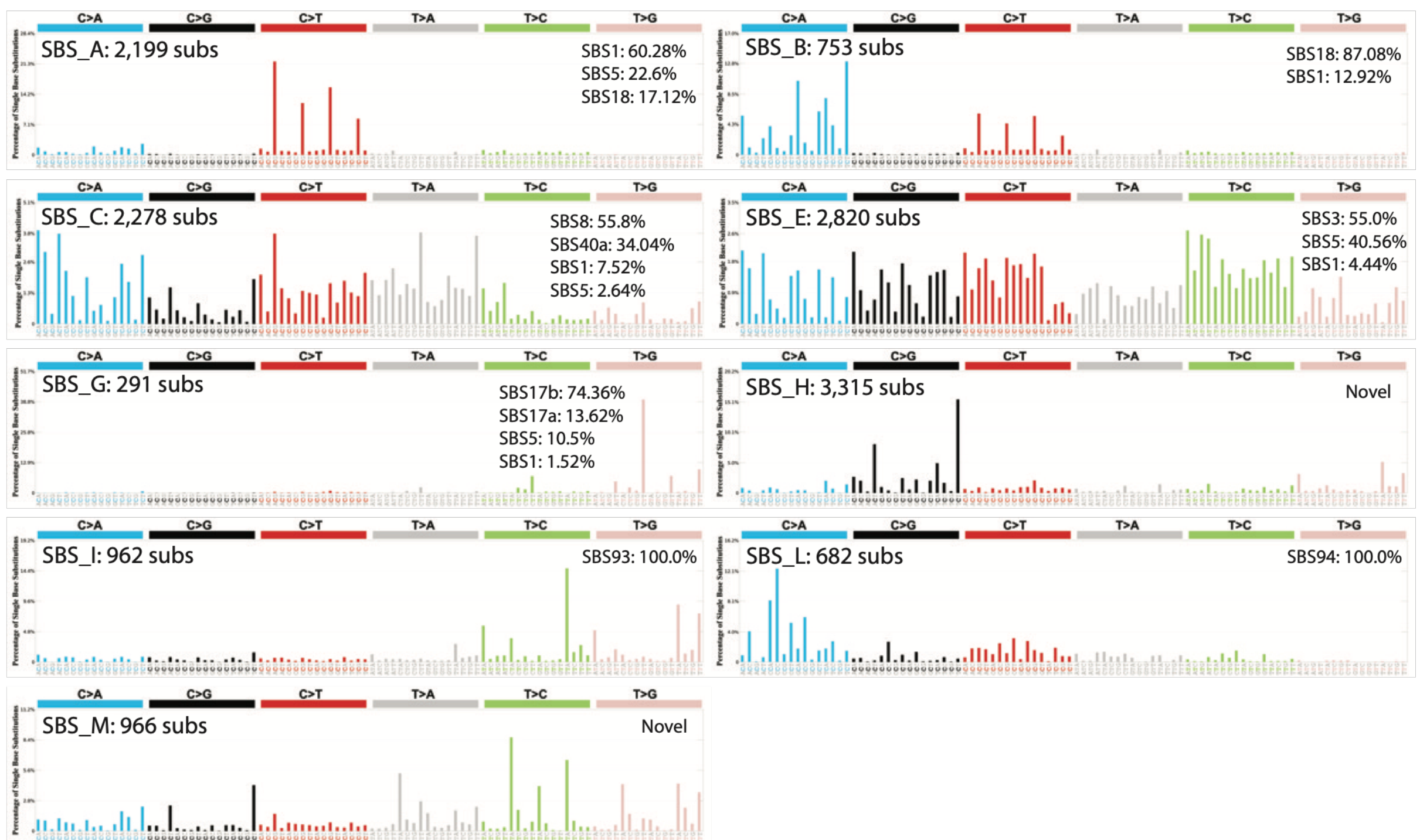

Supplementary Figure 12: Mutational signature activities in a sample with a likely pathogenic *MSH6* mutation. ***(a)*** Mutational profile for sample PD54054a from the Mutographs colorectal cancer cohort, which carries a likely pathogenic mutation in the DNA mismatch repair gene *MSH6*, as identified by CLINVAR. ***(b)*** *De novo* mutational signatures detected in PD54054a via Mutational

Signature Attribution (MSA). Numbers on the left of each signature plot represent the number of single base mutations (subs) attributed to that signature. Percentages on the right indicate the COSMIC decomposition of the corresponding *de novo* signature. Signatures deemed novel by the original Mutographs colorectal cancer study are indicated as such in place of the COSMIC decomposition.

Supplementary Figure 13

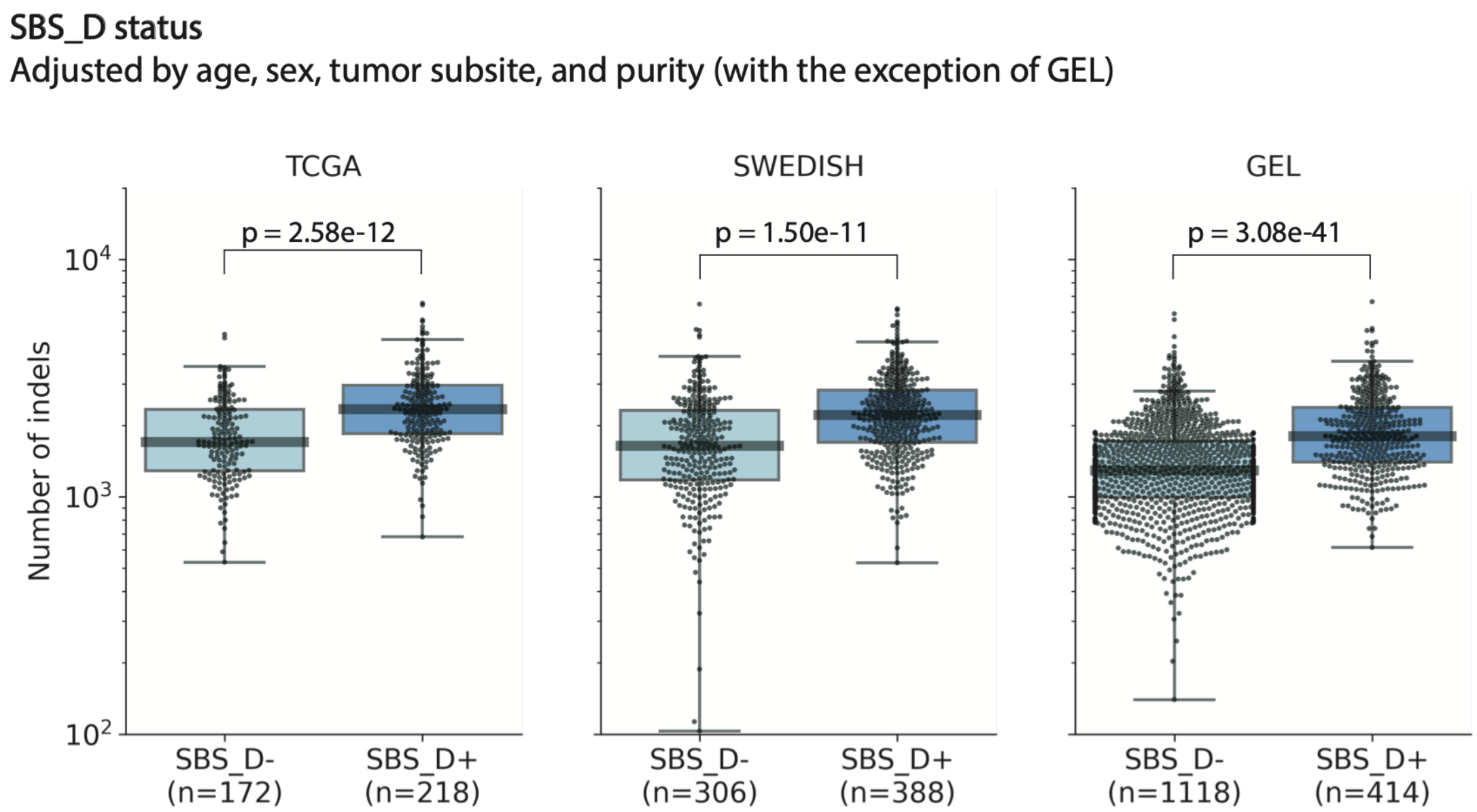

Supplementary Figure 13: SBS_D positive samples have elevated numbers of indels in independent colorectal cancer cohorts. Comparison of the number of indels between SBS_D-positive (SBS_D+) and SBS_D-negative (SBS_D-) samples in The Cancer Genome Atlas (TCGA; left), Swedish (middle), and Genomics England (GEL; right) cohorts. SBS_D+ samples were defined as those with non-zero SBS_D activity in the Mutational Signature Attribution (MSA) results for each cohort. For the Swedish and TCGA cohorts, p-values were calculated using linear models adjusted for age, sex, tumor subsite, and purity. For the GEL cohort, the p-value was calculated using a Mann-Whitney U test due to lack of available metadata. Box plots show the median (center line) and the 25th - 75th percentiles (box bounds); whiskers extend to the most extreme data points within 1.5 times the interquartile range. All data points are shown as dots, including outliers. Source data are provided as a Source Data file.

Supplementary Figure 14

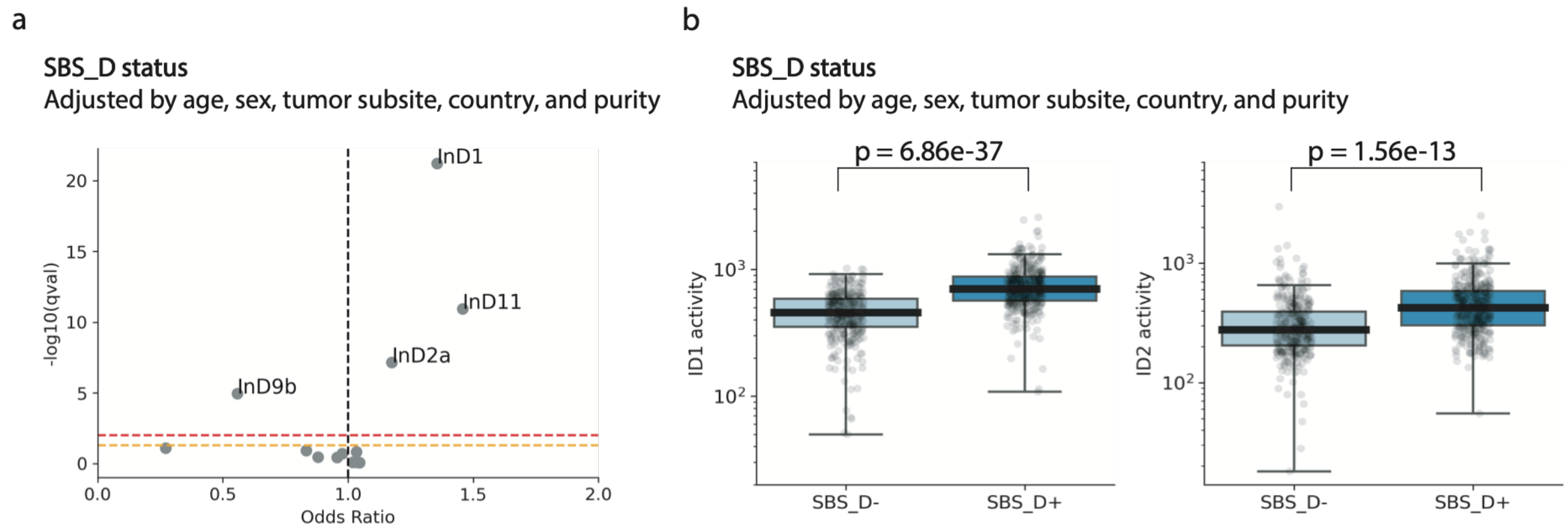

Supplementary Figure 14: Association between SBS_D and the activity of indel mutational signatures using the alternative indel framework from Koh *et al*. *(a)* Volcano plot generated from logistic regression models, one per indel signature (InD), with SBS_D status as the outcome variable and tumor subsite, sex, age, country, purity, and the activity of each indel signature as covariates using the Mutographs CRC cohort. P-values were corrected using the Benjamini-Hochberg method across the indel signatures. Dashed orange line represents a q-value of 0.05, and dashed red line represents a q-value of 0.01; signatures with q-values below 0.05 are labeled. ***(b)*** Comparison of ID1 and ID2 activity (COSMIC) between samples without (SBS_D-; *n*=374) and with (SBS_D+; *n*=428) SBS_D activity in the Mutographs CRC cohort. Significance was derived from logistic regression models, one for ID1 and one for ID2, with SBS_D status as the outcome variable and tumor subsite, sex, age, country, purity, and the activity of either ID1 (left) or ID2 (right) included as covariates. Box plots show the median (center line) and the 25th - 75th percentiles (box bounds); whiskers extend to the most extreme data points within 1.5 times the interquartile range. All data points are shown as dots, including outliers. Source data are provided as a Source Data file.
