# Supplementary material for: Identification and validation of a previously missed mutational signature in colorectal cancer
Source: Nat Commun. 2026 Aug 12;17:9671. doi: 10.1038/s41467-026-76472-w (PMC13558705; doi:10.1038/s41467-026-76472-w)
Supplement: Supplementary file 2 [file 41467_2026_76472_MOESM2_ESM.pdf]

Description of Additional Supplementary Files

Supplementary Data 1: *De novo* signature activities in the Mutographs cohort. The table denotes the activity of *de novo* signatures in the Mutographs CRC cohort. SBS_D-like signature is denoted with a *. Activities were determined using MSA and the *de novo* signatures from SigProfilerExtractor.

Supplementary Data 2: *De novo* signatures in the Mutographs cohort. *De novo* signatures extracted from the Mutographs CRC cohort. SBS_D-like signature is denoted with a *. Signatures were extracted using the SBS-288 context and SigProfilerExtractor.

Supplementary Data 3: *De novo* signature activities in the TCGA cohort. The table denotes the activity of *de novo* signatures in the TCGA CRC cohort. SBS_D-like signature is denoted with a *. Activities were determined using MSA and the *de novo* signatures from SigProfilerExtractor.

Supplementary Data 4: *De novo* signatures in the TCGA cohort. *De novo* signatures extracted from the TCGA CRC cohort. SBS_D-like signature is denoted with a *. Signatures were extracted using the SBS-288 context and SigProfilerExtractor.

Supplementary Data 5: *De novo* signature activities in the Swedish cohort. The table denotes the activity of *de novo* signatures in the Swedish CRC cohort. SBS_D-like signature is denoted with a *. Activities were determined using MSA and the *de novo* signatures from SigProfilerExtractor.

Supplementary Data 6: *De novo* signatures in the Swedish cohort. *De novo* signatures extracted from the Swedish CRC cohort. SBS_D-like signature is denoted with a *. Signatures were extracted using the SBS-288 context and SigProfilerExtractor.

Supplementary Data 7: *De novo* signature activities in the GEL cohort. The table denotes the activity of *de novo* signatures in the GEL CRC cohort. SBS_D-like signature is denoted with a *. Activities were determined using MSA and the *de novo* signatures from SigProfilerExtractor.

Supplementary Data 8: *De novo* signatures in the GEL cohort. *De novo* signatures extracted from the GEL CRC cohort. SBS_D-like signature is denoted with a *. Signatures were extracted using the SBS-96 context and SigProfilerExtractor.

Supplementary Data 9: GSEA results for the overexpressed genes in the TCGA cohort using all SBS_D+ samples. Gene-set enrichment analysis results for the genes found to be overexpressed in the SBS_D+ samples in the TCGA CRC cohort. Analysis was performed between all SBS_D+ and all SBS_D- samples. Significance was assessed using a two-sided Hypergeometric test. Multiple hypothesis testing correction was performed using the Benjamini-Hochberg approach.

Supplementary Data 10: GSEA results for the overexpressed genes in the TCGA cohort using the top 50% SBS_D+ samples. Gene-set enrichment analysis results for the genes found to be overexpressed in the top 50% of SBS_D+ samples ranked by SBS_D activity in the TCGA CRC cohort. Significance was assessed using a two-sided Hypergeometric test. Multiple hypothesis testing correction was performed using the Benjamini-Hochberg approach.

Supplementary Data 11: GSEA results for the overexpressed genes in the TCGA cohort using the top 25% SBS_D+ samples. Gene-set enrichment analysis results for the genes found to be overexpressed in the top 25% of SBS_D+ samples ranked by SBS_D activity in the TCGA CRC cohort. No significantly enriched gene sets were found, thus only the gene names are listed. Significance was assessed using a two-sided Hypergeometric test. Multiple hypothesis testing correction was performed using the Benjamini-Hochberg approach.

Supplementary Data 12: GSEA results for the overexpressed genes in the Swedish cohort using all SBS_D+ samples. Gene-set enrichment analysis results for the genes found to be overexpressed in the SBS_D+ samples in the Swedish CRC cohort. Analysis was performed between all SBS_D+ and all SBS_D- samples. Significance was assessed using a two-sided Hypergeometric test. Multiple hypothesis testing correction was performed using the Benjamini-Hochberg approach.

Supplementary Data 13: GSEA results for the overexpressed genes in the Swedish cohort using the top 50% SBS_D+ samples. Gene-set enrichment analysis results for the genes found to be overexpressed in the top 50% of SBS_D+ samples ranked by SBS_D activity in the Swedish CRC cohort. Significance was assessed using a two-sided Hypergeometric test. Multiple hypothesis testing correction was performed using the Benjamini-Hochberg approach.

Supplementary Data 14: GSEA results for the overexpressed genes in the Swedish cohort using the top 25% SBS_D+ samples. Gene-set enrichment analysis results for the genes found to be overexpressed in the top 25% of SBS_D+ samples ranked by SBS_D activity in the Swedish CRC cohort. Significance was assessed using a two-sided Hypergeometric test. Multiple hypothesis testing correction was performed using the Benjamini-Hochberg approach.

Supplementary Data 15: GSEA results for the overexpressed genes in the Swedish cohort using the top 10% SBS_D+ samples. Gene-set enrichment analysis results for the genes found to be overexpressed in the top 10% of SBS_D+ samples ranked by SBS_D activity in the Swedish CRC

cohort. Significance was assessed using a two-sided Hypergeometric test. Multiple hypothesis testing correction was performed using the Benjamini-Hochberg approach.

Supplementary Data 16: GSEA results for the underexpressed genes in the TCGA cohort using all SBS_D+ samples. Gene-set enrichment analysis results for the genes found to be underexpressed in the SBS_D+ samples in the TCGA CRC cohort. Analysis was performed between all SBS_D+ and all SBS_D- samples. No significantly enriched gene sets were found, thus only the gene names are listed. Significance was assessed using a two-sided Hypergeometric test. Multiple hypothesis testing correction was performed using the Benjamini-Hochberg approach.

Supplementary Data 17: GSEA results for the underexpressed genes in the TCGA cohort using the top 50% SBS_D+ samples. Gene-set enrichment analysis results for the genes found to be underexpressed in the top 50% of SBS_D+ samples ranked by SBS_D activity in the TCGA CRC cohort. Significance was assessed using a two-sided Hypergeometric test. Multiple hypothesis testing correction was performed using the Benjamini-Hochberg approach.

Supplementary Data 18: GSEA results for the underexpressed genes in the TCGA cohort using the top 25% SBS_D+ samples. Gene-set enrichment analysis results for the genes found to be underexpressed in the top 25% of SBS_D+ samples ranked by SBS_D activity in the TCGA CRC cohort. No significantly enriched gene sets were found, thus only the gene names are listed. Significance was assessed using a two-sided Hypergeometric test. Multiple hypothesis testing correction was performed using the Benjamini-Hochberg approach.

Supplementary Data 19: GSEA results for the underexpressed genes in the Swedish cohort using all SBS_D+ samples. Gene-set enrichment analysis results for the genes found to be underexpressed in the SBS_D+ samples in the Swedish CRC cohort. Analysis was performed between all SBS_D+ and all SBS_D- samples. Significance was assessed using a two-sided Hypergeometric test. Multiple hypothesis testing correction was performed using the Benjamini-Hochberg approach.

Supplementary Data 20: GSEA results for the underexpressed genes in the Swedish cohort using the top 50% of SBS_D+ samples. Gene-set enrichment analysis results for the genes found to be underexpressed in the top 50% of SBS_D+ samples ranked by SBS_D activity in the Swedish CRC cohort. Significance was assessed using a two-sided Hypergeometric test. Multiple hypothesis testing correction was performed using the Benjamini-Hochberg approach.

Supplementary Data 21: GSEA results for the underexpressed genes in the Swedish cohort using the top 25% of SBS_D+ samples. Gene-set enrichment analysis results for the genes found to be underexpressed in the top 25% of SBS_D+ samples ranked by SBS_D activity in the Swedish CRC

cohort. Significance was assessed using a two-sided Hypergeometric test. Multiple hypothesis testing correction was performed using the Benjamini-Hochberg approach.

Supplementary Data 22: GSEA results for the underexpressed genes in the Swedish cohort using the top 10% of SBS_D+ samples. Gene-set enrichment analysis results for the genes found to be underexpressed in the top 10% of SBS_D+ samples ranked by SBS_D activity in the Swedish CRC cohort. Significance was assessed using a two-sided Hypergeometric test. Multiple hypothesis testing correction was performed using the Benjamini-Hochberg approach.

Supplementary Data 23: Single base substitution mutations In *POLD1* and DNA mismatch repair genes. All single base substitution mutations found in *POLD1* and the DNA mismatch repair genes (*MLH1*, *MLH3*, *MSH2*, *MSH3*, *MSH6*, *PMS1*, and *PMS2*). Annotations include the sample the mutation was found in, the genomic location of the mutation, and the CLINVAR annotation for the mutation.

Supplementary Data 24: Indel mutations In *POLD1* and DNA mismatch repair genes. All indel mutations found in *POLD1* and the DNA mismatch repair genes (*MLH1*, *MLH3*, *MSH2*, *MSH3*, *MSH6*, *PMS1*, and *PMS2*). Annotations include the sample the mutation was found in, the genomic location of the mutation, and the CLINVAR annotation for the mutation.

Supplementary Data 25: COSMIC decomposed single base substitution mutation activities with SBS_D included as a novel signature. Signature activities for the Mutographs CRC cohort determined by MSA for the COSMIC decomposed signatures with SBS_D not decomposed and instead transformed to its SBS-96 version and included as a novel mutational signature.
