# Supplementary material for: Identification and validation of a previously missed mutational signature in colorectal cancer
Source: Nat Commun. 2026 Aug 12;17:9671. doi: 10.1038/s41467-026-76472-w (PMC13558705; doi:10.1038/s41467-026-76472-w)
Supplement: Supplementary file 3 [file 41467_2026_76472_MOESM4_ESM.pdf]

nature portfolio

Corresponding author(s): Ludmil B. Alexandrov

Last updated by author(s): Jul 26, 2026

Reporting Summary

Nature Portfolio wishes to improve the reproducibility of the work that we publish. This form provides structure for consistency and transparency in reporting. For further information on Nature Portfolio policies, see our Editorial Policies and the Editorial Policy Checklist.

Statistics

For all statistical analyses, confirm that the following items are present in the figure legend, table legend, main text, or Methods section.

n/a	Confirmed	
☐	☒	The exact sample size (*n*) for each experimental group/condition, given as a discrete number and unit of measurement
☐	☒	A statement on whether measurements were taken from distinct samples or whether the same sample was measured repeatedly
☐	☒	The statistical test(s) used AND whether they are one- or two-sided<br>*Only common tests should be described solely by name; describe more complex techniques in the Methods section.*
☐	☒	A description of all covariates tested
☐	☒	A description of any assumptions or corrections, such as tests of normality and adjustment for multiple comparisons
☐	☒	A full description of the statistical parameters including central tendency (e.g. means) or other basic estimates (e.g. regression coefficient) AND variation (e.g. standard deviation) or associated estimates of uncertainty (e.g. confidence intervals)
☐	☒	For null hypothesis testing, the test statistic (e.g. *F*, *t*, *r*) with confidence intervals, effect sizes, degrees of freedom and *P* value noted<br>*Give P values as exact values whenever suitable.*
☒	☐	For Bayesian analysis, information on the choice of priors and Markov chain Monte Carlo settings
☒	☐	For hierarchical and complex designs, identification of the appropriate level for tests and full reporting of outcomes
☐	☒	Estimates of effect sizes (e.g. Cohen's *d*, Pearson's *r*), indicating how they were calculated

Our web collection on statistics for biologists contains articles on many of the points above.

Software and code

Policy information about availability of computer code

Data collection

Mutographs [PMID: 40267983], no software used
Swedish Cohort [PMID: 39112715], no software used
TCGA Cohort accessed via GDC data portal, downloaded using gdc-client v1.6.1 (https://github.com/NCI-GDC/gdc-client)
Genomics England Cohort [PMID: 35949260], no software used

Data analysis

SigProfilerMatrixGenerator v1.2.0 (https://github.com/AlexandrovLab/SigProfilerMatrixGenerator)
SigProfilerExtractor v1.1.21 (https://github.com/AlexandrovLab/SigProfilerExtractor)
SigProfilerAssignment v0.0.29 (https://github.com/AlexandrovLab/SigProfilerAssignment)
SigProfilerTopography v1.0 (https://github.com/AlexandrovLab/SigProfilerTopography)
MSA v2.0 (https://gitlab.com/s.senkin/MSA)
Mutect2 GATK v4.1.4.1 (https://github.com/broadinstitute/gatk)
MuSiCal v1.0.0 (https://github.com/parklab/MuSiCal)
SignatureToolsLib v2.4.6 (https://github.com/Nik-Zainal-Group/signature.tools.lib)
Limma v3.46.0 (https://bioconductor.org/packages/release/bioc/html/limma.html)
edgeR v3.32.1 (https://bioconductor.org/packages/release/bioc/html/edgeR.html)
R v4.0.4

statsmodels Python package v0.13.2
Statistical analysis was performed with Python v3.9.7

Code used for figure generation: https://github.com/AlexandrovLabPublications/SBS_D_manuscript_code

For manuscripts utilizing custom algorithms or software that are central to the research but not yet described in published literature, software must be made available to editors and reviewers. We strongly encourage code deposition in a community repository (e.g. GitHub). See the Nature Portfolio guidelines for submitting code & software for further information.

Data

Policy information about availability of data

All manuscripts must include a data availability statement. This statement should provide the following information, where applicable:

- Accession codes, unique identifiers, or web links for publicly available datasets
- A description of any restrictions on data availability
- For clinical datasets or third party data, please ensure that the statement adheres to our policy

Somatic mutation data and metadata for the Mutographs CRC cohort were previously published and are available at the European Genome-phenome Archive (EGA) under accession code EGAS00001003774 [https://ega-archive.org/studies/EGAS00001003774] under restricted access, which can be acquired via the EGA website (https://ega-archive.org/access/request-data/how-to-request-data/). The mutational signature SBS_D is available as part of Supplementary Table 2. Somatic mutation data for the Swedish CRC cohort were previously published and are publicly available at the European Variation Archive under accession number PRJEB61514 [https://www.ebi.ac.uk/ena/browser/view/PRJEB61514]; metadata for the Swedish cohort are available as Supplementary Table 1 from the original publication. Gene expression data for the Swedish CRC cohort were previously published and are publicly available at ArrayExpress under accession number E-MTAB-12862 [https://www.ebi.ac.uk/biostudies/arrayexpress/studies/E-MTAB-12862]. Somatic mutation data for the GEL CRC cohort were previously published and are available as Supplementary Table 7 from the original publication. Raw sequencing data for the TCGA CRC cohort are available at the GDC Data Portal (https://portal.gdc.cancer.gov/) under restricted access, which can be obtained via dbGaP (https://dbgap.ncbi.nlm.nih.gov/aa/wga.cgi?page=login). Metadata for the TCGA cohort are publicly available at https://portal.gdc.cancer.gov/. Gene expression data for the TCGA CRC cohort are publicly available on the GDC data portal (https://portal.gdc.cancer.gov/). The COSMIC reference mutational signatures (v.3.4) were downloaded from COSMIC (https://cancer.sanger.ac.uk/signatures/downloads/), and the Signal reference mutational signatures were obtained from Supplementary Table 21 from the original publication. The SBS_D signature (provisionally designated SBS111) has been submitted to the COSMIC database. Source data are provided with this paper.

Research involving human participants, their data, or biological material

Policy information about studies with human participants or human data. See also policy information about sex, gender (identity/presentation), and sexual orientation and race, ethnicity and racism.

Reporting on sex and gender	N/A
Reporting on race, ethnicity, or other socially relevant groupings	N/A
Population characteristics	N/A
Recruitment	N/A
Ethics oversight	N/A

Note that full information on the approval of the study protocol must also be provided in the manuscript.

Field-specific reporting

Please select the one below that is the best fit for your research. If you are not sure, read the appropriate sections before making your selection.

[X] Life sciences [ ] Behavioural & social sciences [ ] Ecological, evolutionary & environmental sciences

For a reference copy of the document with all sections, see nature.com/documents/nr-reporting-summary-flat.pdf

Life sciences study design

All studies must disclose on these points even when the disclosure is negative.

Sample size	The analysis was performed on all samples available across the 4 cohorts used in the the study. No statistical test was used to predetermine sample size.
Data exclusions	Samples were excluded from the TCGA, Swedish, and Genomics England cohorts. Hypermutator samples, defined as those with over 100,000 single base substitution mutations or over 7,000 insertion/deletion mutations, were removed. Additionally, any sample with less than 1,000 single base substitution mutations was removed. Further, samples with cosine similarity greater than 0.80 to SBS36 or SBS30 from COSMIC or SBS108 from SIGNAL were removed, to account for mutations in MUTYH, NTLH1, and OGG1. Lastly, 2 additional samples from the Swedish cohort were removed for having high levels of SBS15 despite not being hypermutators.
Replication	The reproducibility of the extraction of the mutational signature discussed in this study was verified in three independent cohorts, detailed in the data section. All attempts at replication were successful.
Randomization	Randomization is not applicable to this study as no interventions were tested, thus cases were never separated into experimental groups.
Blinding	Blinding is not applicable to this study as no interventions were tested.

Reporting for specific materials, systems and methods

We require information from authors about some types of materials, experimental systems and methods used in many studies. Here, indicate whether each material, system or method listed is relevant to your study. If you are not sure if a list item applies to your research, read the appropriate section before selecting a response.

Materials & experimental systems

n/a	Involved in the study	
☒	☐	Antibodies
☒	☐	Eukaryotic cell lines
☒	☐	Palaeontology and archaeology
☒	☐	Animals and other organisms
☒	☐	Clinical data
☒	☐	Dual use research of concern
☒	☐	Plants

Methods

n/a	Involved in the study	
☒	☐	ChIP-seq
☒	☐	Flow cytometry
☒	☐	MRI-based neuroimaging

Plants

Seed stocks	N/A
Novel plant genotypes	N/A
Authentication	N/A
