# Supplementary material for: Identification and validation of a previously missed mutational signature in colorectal cancer
Source: Nat Commun. 2026 Aug 12;17:9671. doi: 10.1038/s41467-026-76472-w (PMC13558705; doi:10.1038/s41467-026-76472-w)
Supplement: Supplementary file 4 [file 41467_2026_76472_MOESM5_ESM.pdf]

nature portfolio

Peer Review File

Identification and Validation of a Previously Missed Mutational Signature in Colorectal Cancer

Corresponding Author: Professor Ludmil Alexandrov

This file contains all reviewer reports in order by version, followed by all author rebuttals in order by version.

Version 0:

Reviewer comments:

Reviewer #1

(Remarks to the Author)
Kazachkova and colleagues perform a thorough reanalysis of the mutational landscape of colorectal cancers, revisiting the SBS_D mutational signature that was previously deemed to be a composite signature containing a SBS18 component. Based on signature extractions and decompositions in various cohorts, topographical analysis and molecular timing, they convincingly show that SBS_D mutations do not match the characteristics of SBS18. While the analysis they perform is thorough and of high quality, I have some questions regarding the author's interpretation of the presented results. I recommend the paper for publication in Nature Communications after the following points have been addressed:

1. Based on the fact that the SBS_D signature cannot be adequately reconstructed by the COSMIC signatures used in the original analysis, they conclude that SBS_D is a novel distinct signature, rather than a composite signature. However, when they perform naive decomposition including the full set of COSMIC signatures, they show that the SBS_D signature is well reconstructed by known signatures, including the repair-deficient SBS10c and SBS15 signatures. Can the authors exclude that the SBS_D signature is in fact a composite signature, where a subset of its mutations were misassigned to SBS18 in the initial analysis due to leaving out repair-deficiency-associated signatures?

2. Related to the point above, similar to what they show for the C>A component of SBS_D and SBS18, could the authors demonstrate that the remaining substitutions classes are also distinct from the COSMIC signatures identified in the decomposition (e.g.: are the T>A mutation characteristics distinct from SBS34)?

3. Recently a new Indel classification was proposed to better separate indels associated with MMR or replicative polymerase dysfuncton(PMID: 40210680). Given that the authors find increased indels associated with SBS_D (Figure 5a), they should reanalyze indel signatures with this new classification to look for evidence of MMR or replicative polymerase dysfunction linked to SBS_D mutations.

4. In the discussion section the authors hypothesize why the distinct SBS_D signature was not previously identified. They propose that due to combined analysis of repair-proficient and deficient samples, the presence of SBS_D might be masked or overwhelmed by repair-deficient signatures in hypermutated cancers that share features with the SBS_D signature. Given that the authors show that SBS_D is well reconstructed by a set of signatures including these repair-deficiency signatures, would a simpler explanation not be that the SBS_D signature is a composite signature containing SBS10c and SBS15 components caused by these mild or non-canonical repair deficiencies?

5. SBS_D in Figure 1 seems different than SBS_D in supplementary figure 1, more specifically the C>T component, why is this? Different cohorts?

Reviewer #2

(Remarks to the Author)
Thank you for the opportunity to review the manuscript by Kazachkova et al. The study analysed 802 treatment naïve MSS CRCs from the Mutographs project and identified a de novo signature referred to as SBS_D that was investigated as a novel signature. SBS_D was identified in three additional CRC cohorts using the same approach. Key findings were: 1) decomposition of SBS_D into COSMICv3.4 clocklike, SBS18,and SBS34 signatures, 2) using a decomposition with a

restricted set of signatures, the patterns of SBS_D align with signatures previously associated with deficiencies in POLD1 and mismatch repair, 3) evidence that SBS_D represented a late mutational event/process, 4) high indel mutation rate, and 5) topography analysis showing SBS_D mutations were depleted near nucleosomes. The findings are potentially interesting but need further clarity, presentation and interpretation to improve the validity and relevance of the results.

Major concerns
1) A more comprehensive argument for the signature's novelty by comparing to the full set of COSMIC 3.4 signatures - does SBS_D match/have strong cosine similarity with any of the Signal signature definitions? Additionally, many comparisons are between SBS_D and SBS18 – a similar comparison to other signatures (particularly SBS10c) may be informative.
2) Does SBS_D co-occur with other signatures, especially SBS18 and SBS10c? A correlation matrix across samples may help establish SBS_D as a distinct process or composite/proxy for other existing signatures.
3) It isn't clear why the de novo analysis did not identify SBS_D when dMMR samples were included if SBS_D is not a dMMR related signature? This part of the analysis and results needs further explanation.
4) It is not clear how the authors define the presence of SBS_D, this should be included and clearly defined. Providing a graph of the proportion of SBS_D out of total signature proportion of each CRC across the MSS CRCs from each of the cohorts would be informative and demonstrate dominance/contribution of SBS_D to entire mutational processes.
5) The finding that SBS_D was identified in 428/802 or 53.3% of MSS samples but contributing only 8.5% of all mutations needs further clarity as it suggests that a low threshold was used for considering SBS_D to be present. This would suggest SBS_D is a composite of low-level background signatures and not a dominant mutational process. Discussion of SBS_D in the context of existing highly prevalent signatures (SBS1, SBS5) would help better understand the biological role of SBS_D.
6) The manuscript does not explore associations with clinical or pathological features. The relevance of the SBS_D signature could be further explored given the data available from the CRC cohorts included. Is there an association with age at diagnosis, tumor anatomical site, stage and other mutations, pathways etc needs to be included to provide some further resolution of this signature.
7) Further investigation of other DNA repair genes may be useful to determine if there is an association with SBS_D outside of POLD1 and DNA mismatch repair genes. This may be particularly relevant given its cosine similarity to SBS18, and the observation of high indel mutation numbers.
8) Are the indel mutations early/late events which may help establish how the elevated indel counts link to SBS_D.
9) The Figure legends lack important detail throughout and should be improved.
10) The discussion needs to include text on the interpretation of the results for nucleosome and topography and what this means, particularly in the context of other signatures to better understand how significant the SBS_D result is – comparing the results to other known signatures may aid interpretation regarding nucleosome occupancy.
11) There is some redundancy in text related to describing how signatures are measured. The first paragraph of the results is largely duplicated with the text in the methods section. The description in the results should be moved and merged with the text in the methods. This will allow inclusion of text in the discussion on nucleosome and topography results as suggested above.
12) The similarity between SBS_D and SBS10c is present only after removal of signatures in the naïve analysis. Is removing signatures introducing bias by forcing mutations to be assigned to less relevant signatures? This should be addressed by the authors.
13) Is the application of the same signature methodology in the additional 3 cohorts really a validation of the SBS_D signature or just forcing the same outcome? [is there any overlap in these datasets]
14) Why do the authors think SBS_D was lower in GEL compared with the other cohorts? Is this a significant difference? Can this be quantified?
15) The graph shows lower single base and indel burden for MSS compared with MSI and POLD1 CRCs but the fraction of genome aberrant is higher. Can the authors please provide some clarity on how this derived, and intereptation of this result. the text also says higher indels??
16) Given the high indel count, could SBS_D be related to EMAST?

Reviewer #3

(Remarks to the Author)
I co-reviewed this manuscript with one of the reviewers who provided the listed reports. This is part of the Nature Communications initiative to facilitate training in peer review and to provide appropriate recognition for Early Career Researchers who co-review manuscripts.

Version 1:

Reviewer comments:

Reviewer #1

(Remarks to the Author)
The authors have comprehensively dealt with my points and I recommend the manuscript for publication in Nature communications

Reviewer #4

(Remarks to the Author)

Most previous comments from Reviewer 2 have been appropriately addressed in the revised manuscript. There are some remaining issues that could further improve the study.

Previous comments 2, 6, and 7: explore associations of SBS_D with other features, including mutational signatures (comment 2), clinicopathological features and driver mutations (comments 6 and 7). The authors have mostly addressed these comments, but the additional data have augmented the limitation that signature SBS_D seems to be an independent mutational process with no relevant associations and an unclear biological role and relevance. Pathway analyses were proposed and might be helpful in detecting phenotypic consequences but were not included in the revision. Corresponding RNA-seq data are available in at least some of the cohorts and should be included for such analyses. Furthermore, analysis of associations with clinicopathological features (in response to comment 6) was based on categorizing SBS_D activity as a binary 'yes/no' feature. Could the authors explore potential associations using SBS_D activity as a continuous score, and evaluate potential characteristics of the high-scoring samples? (This is also related to previous comment 4 and the new graphs in Supplementary Figure 5 showing varying contributions from SBS_D among the SBS_D positive samples.) Finally, reanalysis of late clonal mutations only (enriched in SBS_D) could help to unmask any potential associations not identified when also including early clonal events in the analysis. For example, is the sample-wise proportion of SBS_D among late clonal mutations associated with any clinicopathological features or driver mutations?

Previous comment 11: redundancy in text. The revised manuscript is occasionally repetitive and there is potential to improve this, particularly for the text recapitulating data and interpretations from the preceding and previously published study (reference 9, Diaz-Gay et al.).

For the purpose of data reproducibility, the authors should consider sharing the mutation signature activities calculated per sample in the external cohorts.

Reviewer #5

(Remarks to the Author)
I co-reviewed this manuscript with one of the reviewers who provided the listed reports. This is part of the Nature Communications initiative to facilitate training in peer review and to provide appropriate recognition for Early Career Researchers who co-review manuscripts.

Version 2:

Reviewer comments:

Reviewer #4

(Remarks to the Author)
We thank the authors for the additional data and analyses. All previous comments have been addressed, and we consider the new data to have strengthened the study. We appreciate that the authors have submitted the new SBS_D signature to the COSMIC database under the classification of unknown etiology.

Reviewer #5

(Remarks to the Author)
I co-reviewed this manuscript with one of the reviewers who provided the listed reports. This is part of the Nature Communications initiative to facilitate training in peer review and to provide appropriate recognition for Early Career Researchers who co-review manuscripts.

Open Access This Peer Review File is licensed under a Creative Commons Attribution 4.0 International License, which permits use, sharing, adaptation, distribution and reproduction in any medium or format, as long as you give appropriate credit to the original author(s) and the source, provide a link to the Creative Commons license, and indicate if changes were made.
In cases where reviewers are anonymous, credit should be given to 'Anonymous Referee' and the source.
The images or other third party material in this Peer Review File are included in the article's Creative Commons license, unless indicated otherwise in a credit line to the material. If material is not included in the article's Creative Commons license and your intended use is not permitted by statutory regulation or exceeds the permitted use, you will need to obtain permission directly from the copyright holder.
To view a copy of this license, visit https://creativecommons.org/licenses/by/4.0/

Color/Font Legend: Reviewers' Comments, Authors' Responses, *Revised Manuscript Excerpts*

Reviewer #1 (Remarks to the Author):

Kazachkova and colleagues perform a thorough reanalysis of the mutational landscape of colorectal cancers, revisiting the SBS_D mutational signature that was previously deemed to be a composite signature containing a SBS18 component. Based on signature extractions and decompositions in various cohorts, topographical analysis and molecular timing, they convincingly show that SBS_D mutations do not match the characteristics of SBS18. While the analysis they perform is thorough and of high quality, I have some questions regarding the author's interpretation of the presented results. I recommend the paper for publication in Nature Communications after the following points have been addressed:

Comment: 1. Based on the fact that the SBS_D signature cannot be adequately reconstructed by the COSMIC signatures used in the original analysis, they conclude that SBS_D is a novel distinct signature, rather than a composite signature. However, when they perform naive decomposition including the full set of COSMIC signatures, they show that the SBS_D signature is well reconstructed by known signatures, including the repair-deficient SBS10c and SBS15 signatures. Can the authors exclude that the SBS_D signature is in fact a composite signature, where a subset of its mutations were misassigned to SBS18 in the initial analysis due to leaving out repair-deficiency-associated signatures?

Response: The reviewer raises an excellent point. While SBS_D can mathematically be decomposed into signatures associated with POLD1-deficiency (SBS10c) and DNA mismatch repair deficiency (SBS15), along with smaller contributions from clock-like signatures and SBS34, we find no evidence of these underlying defects in the samples where SBS_D occurs. Two main observations support treating SBS_D as a distinct process rather than a composite signature:

1. **Etiology mismatch:** The causes of SBS10c (POLD1-deficiency) and SBS15 (mismatch repair deficiency) are well established, but we find no evidence of these deficiencies in the samples where SBS_D is present. For example, in the Mutographs cohort, we detect neither the expected causal genomic aberrations, such as pathogenic mutations in *POLD1* or DNA mismatch repair genes, nor the characteristic phenotypes, such as hypermutation of single nucleotide variants and indels, in the 428 CRC samples that harbor SBS_D.

2. **Consistency across cohorts:** SBS_D-like signatures show highly consistent peaks across all examined cohorts. If SBS_D were simply a mixture, we would expect to see more evidence of its components emerging separately in the four different datasets examined in our study. Instead, SBS_D consistently appears as a stable, coherent signature.

Because SBS_D is observed in DNA repair-proficient samples and shows reproducibility across four independent CRC cohorts, we find insufficient evidence to regard it as a composite of known DNA repair-deficiency signatures. Moreover, imposing such a decomposition could misleadingly

suggest DNA repair defects in samples that are DNA repair-proficient. We agree with the reviewer that this is an important detail, and we have expanded the *Discussion* in the revised manuscript to clarify these points.

DISCUSSION

"While SBS_D can be mathematically decomposed into signatures associated with DNA repair deficiency, such as SBS10c and SBS15, our SBS_D-positive samples show no genomic or phenotypic evidence of DNA repair deficiency. Although these tumors display features suggestive of mild repair infidelity, such as elevated indel burdens compared to SBS_D-negative samples, they lack the characteristic phenotypes of repair-deficient tumors and pathogenic mutations in known DNA repair genes. The consistency of SBS_D across multiple CRC cohorts and the lack of evidence of DNA repair-deficiency in SBS_D-positive samples support its classification as a distinct signature, potentially associated with DNA repair infidelity, rather than a composite of known repair-deficiency signatures."

Comment: 2. Related to the point above, similar to what they show for the C>A component of SBS_D and SBS18, could the authors demonstrate that the remaining substitutions classes are also distinct from the COSMIC signatures identified in the decomposition (e.g.: are the T>A mutation characteristics distinct from SBS34)?

Response: We thank the reviewer for raising this point. We have now extended our analysis to all six substitution classes (C>A, C>T, C>G, T>A, T>C, and T>G) and compared SBS_D to the COSMIC signatures identified in its naïve decomposition—SBS10c, SBS15, SBS34, SBS1, and SBS5 (**Response Figure R1.1**). Across these classes, the C>A pattern of SBS_D closely matches SBS10c (cosine similarity [cs] = 0.97), the C>G pattern is similar to SBS5 (cs = 0.94) and SBS10c (cs = 0.92), the C>T pattern resembles SBS1 (cs = 0.86) and SBS10c (cs = 0.85), the T>A pattern shows a near-perfect match to SBS34 (cs = 0.99), the T>C pattern aligns with SBS5 (cs = 0.94) and SBS10c (cs = 0.94), and the T>G pattern is consistent with SBS5 (cs = 0.86). These results indicate that the individual mutation types within SBS_D are not distinct from those seen in several established COSMIC signatures. However, no single COSMIC signature recapitulates the overall SBS_D pattern: the highest cosine similarity across all 96 mutational channels is 0.79 with SBS10c (**Response Figure R1.1**). For reference, the average cosine similarity between two random nonnegative vectors is ~0.75, and signatures are generally considered to represent the same mutational process only when the cosine similarity exceeds 0.90.

These similarities are not unexpected, as the naïve decomposition almost perfectly reconstructs SBS_D (cs = 0.98). However, as noted in the manuscript, we do not regard this decomposition as

biologically valid. Specifically, SBS10c and SBS15—both present in the naïve decomposition—are associated with DNA repair deficiency, whereas SBS_D was extracted exclusively from DNA-repair-proficient samples. This clear etiologic mismatch provides the primary rationale for rejecting the naïve decomposition. Moreover, the SBS_D pattern is highly consistent across four independent cohorts, further supporting its interpretation as a distinct and reproducible signature. While SBS_D resembles some signatures in its naïve decomposition for specific mutation types, this does not provide sufficient biological basis for its decomposition.

We appreciate the reviewer's comment about examining the similarity between SBS_D substitution subtypes and its naïve decomposition components and acknowledge that future refinements may become appropriate as additional data emerge. Indeed, COSMIC mutational signatures have evolved over time—such as SBS40 being subdivided into SBS40a–c and SBS5 being refined as larger datasets became available. Importantly, even if SBS_D were to be split into separate components in the future, such as SBS10c and SBS15, the biological insight would remain: a process likely related to DNA repair infidelity appears to be active in DNA-repair-proficient samples. We have updated the *Discussion* to explicitly note this possibility.

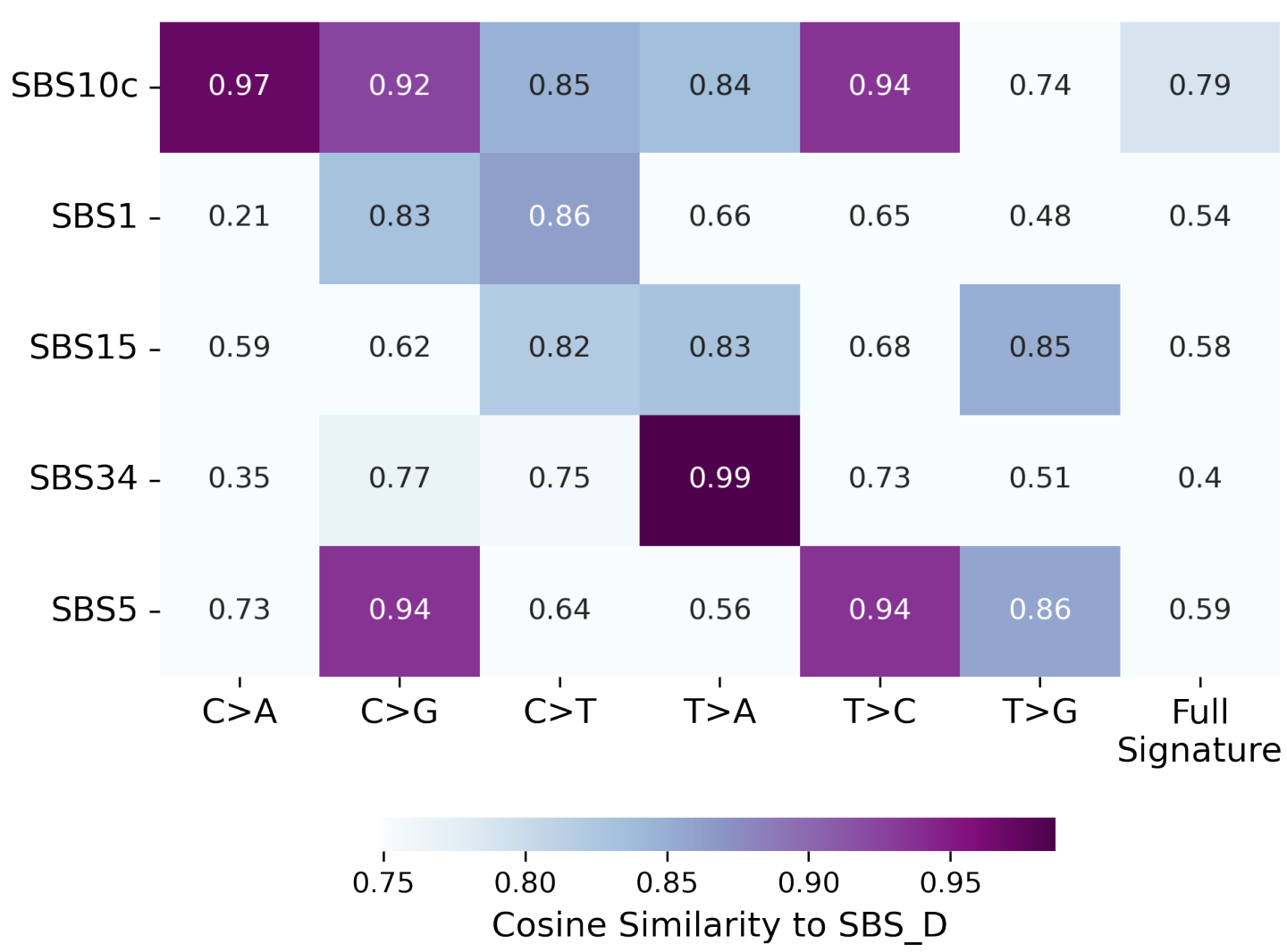

Response Figure R1.1: Comparison of the substitution classes between SBS_D and the COSMIC signatures in its naïve **decomposition.** Comparison between SBS_D and the COSMIC mutational signatures present in its naïve decomposition. The contribution of each of the 16 channels within a substitution class is normalized across the 16 channels. Cosine similarity is calculated using the 16 channels between SBS_D and the respective COSMIC signature. The final column represents the cosine similarity between SBS_D and each COSMIC signatures using all 96 contexts. SBS_D was collapsed to its SBS-96 context for this analysis.

DISCUSSION

"The consistency of SBS_D across multiple CRC cohorts and the lack of evidence of DNA repair-deficiency in SBS_D-positive samples support its classification as a distinct signature, potentially associated with DNA repair infidelity, rather than a composite of known repair-deficiency signatures. However, as the field of mutational signatures is ever-evolving with the generation of larger whole-genome sequenced cohorts, we acknowledge that we cannot exclude the possibility that SBS_D will be separated out into smaller components or otherwise modified as more data

become available. Despite this, our evidence suggests that the signal observed through SBS_D likely represents a mutational process previously thought to be absent from DNA repair-proficient CRC samples.

Comment: 3. Recently a new Indel classification was proposed to better separate indels associated with MMR or replicative polymerase dysfuncton(PMID: 40210680). Given that the authors find increased indels associated with SBS_D (Figure 5a), they should reanalyze indel signatures with this new classification to look for evidence of MMR or replicative polymerase dysfunction linked to SBS_D mutations.

Response: We thank the reviewer for this insightful suggestion and agree that the new indel classification provides an important opportunity to test for potential links between SBS_D and DNA repair or polymerase dysfunction. Briefly, we used SigProfilerExtractor to extract Indel mutational signatures using the new indel classification framework developed by Koh *et al.* from the Mutographs CRC data. We then decomposed the *de novo* signatures to the set of reference signatures developed as part of the Koh *et al.* study and used SigProfilerAssignment to attribute the decomposed signatures to each sample. Finally, we used logistic regression models, one per signature, to identify any associations between the activity of any indel signature and SBS_D (**Response Figure R1.2*a***).

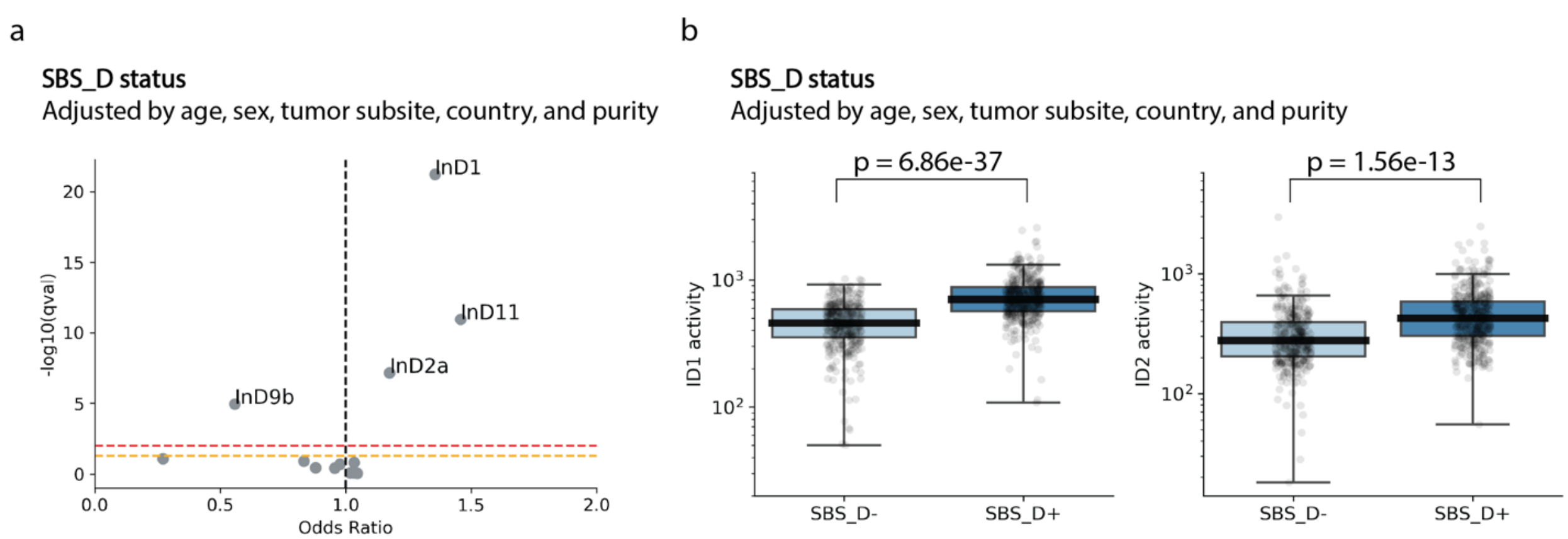

Response Figure R1.2: Association between SBS_D and the activity of indel mutational signatures using the new indel framework from Koh *et al.* *(a)* Volcano plot generated from logistic regression models, one per indel signature (InD), with SBS_D status as the outcome variable and tumor subsite, sex, age, country, purity, and the activity of each indel signature as covariates using the Mutographs CRC cohort. P-values were corrected using the Benjamini-Hochberg method across the indel signatures. Dashed orange line represents a q-value of 0.05, and dashed red line represents a q-value of 0.01; signatures with q-values below 0.05 are labeled. ***(b)*** Comparison of ID1 and ID2 activity (COSMIC) between samples without (SBS_D-) and with (SBS_D+) SBS_D activity in the Mutographs CRC cohort. Significance was derived from logistic regression models, one for ID1 and one for ID2, with SBS_D status as the outcome variable and tumor subsite, sex, age, country, purity, and the activity of either ID1 (left) or ID2 (right) included as covariates.

We observed that InD9b (no similar COSMIC signature and no proposed etiology in the Koh *et al.* study) was negatively associated with SBS_D activity. Additionally, InD1 and InD2a (similar to COSMIC ID1 and ID2, respectively), and InD11 (no matching COSMIC indel signature) were positively associated with SBS_D. As the reviewer noted, SBS_D was associated with increased indels in our study. Because InD1 and InD2a are the most common indel signatures (found in 87% and 69% of all Mutographs CRC samples, respectively), this result was expected and aligns with what we observe in the Mutographs CRC data for ID1 and ID2 (**Response Figure R1.2*b***). The positive association between SBS_D and InD11 was of potential interest, as Koh *et al.* stated "InD11 appeared related to InD1 and might be an oversplit signature frequently enriched in high InDel burden samples, such as those with MMRd and Pol-dys.". This could suggest that Ind11, like we hypothesize for SBS_D, may be capturing a process related to polymerase proofreading and mismatch repair. We have now also included this result in the revised manuscript as **Supplementary Figure 10** and modified the text accordingly.

RESULTS

*"Fourth, using the recently published new framework for indel signatures[28], which identified indel signatures associated with DNA repair deficiency, we observed that InD9b (no similar COSMIC signature and no proposed etiology) was negatively associated with SBS_D activity (**Supplementary Figure 10a**), whereas InD1 and InD2a (similar to COSMIC ID1 and ID2, respectively, which are elevated in SBS_D-positive samples in the Mutographs CRC cohort; **Supplementary Figure 10b**), and InD11 (no matching COSMIC signature) were positively associated with SBS_D (**Supplementary Figure 10a**). InD11 was previously found to be enriched in samples with high indel burdens, such as those with DNA repair deficiencies[28], further suggesting at a relationship between SBS_D and DNA repair."*

<u>Comment:</u> 4. In the discussion section the authors hypothesize why the distinct SBS_D signature was not previously identified. They propose that due to combined analysis of repair-proficient and deficient samples, the presence of SBS_D might be masked or overwhelmed by repair-deficient signatures in hypermutated cancers that share features with the SBS_D signature. Given that the authors show that SBS_D is well reconstructed by a set of signatures including these repair-deficiency signatures, would a simpler explanation not be that the SBS_D signature is a composite signature containing SBS10c and SBS15 components caused by these mild or non-canonical repair deficiencies?

<u>Response:</u> We agree with the reviewer that one cannot completely exclude the possibility that SBS_D represents a composite of SBS10c and SBS15 arising from mild or non-canonical forms

of DNA repair deficiency. However, despite extensive analyses, we did not find evidence of such deficiencies in any SBS_D-positive samples—either at the genomic level (no pathogenic alterations in *POLD1* or mismatch repair genes) or at the phenotypic level (no hypermutation or the characteristic genomic stability profiles typically associated with these deficiencies). We acknowledge that there may be other, yet uncharacterized or obscured, mechanisms leading to subtle defects in DNA repair that were not captured by our analyses.

Thus, while the naïve decomposition highlights similarities between SBS_D and DNA repair-deficiency signatures such as SBS10c and SBS15, we interpret this resemblance as a clue to SBS_D's etiology rather than evidence that these pathways are deficient in our CRC samples. A plausible alternative is that an internal or external process generates enough replication-associated mutations to temporarily overwhelm repair systems, producing patterns that resemble DNA repair deficiency without being caused by it. Although further experimental work will be required to establish its origin, the absence of genomic or phenotypic evidence of repair defects in SBS_D-positive tumors strongly argues against SBS10c or SBS15 as the underlying cause. Instead, the combination of repair proficiency, evidence of repair infidelity, and reproducibility across four independent CRC cohorts supports SBS_D as a novel mutational signature rather than a composite of existing repair-deficiency signatures. We now further clarify this point in the revised manuscript.

RESULTS

"Similarly, we observed no difference in SBS_D activity between samples with and without loss of heterozygosity in POLD1 or DNA mismatch repair genes…These results indicate that the samples used for the extraction of SBS_D do not harbor known or detectable deficiencies in DNA repair pathways."

DISCUSSION

"Overall, despite extensive studies on CRC mutational landscapes, our findings reveal a previously unrecognized mutational signature in DNA repair-proficient CRC that may reflect DNA repair infidelity during the late stages of CRC evolution. While SBS_D can be mathematically decomposed into signatures associated with DNA repair deficiency, such as SBS10c and SBS15, our SBS_D-positive samples show no genomic or phenotypic evidence of DNA repair deficiency. Although these tumors display features suggestive of mild repair infidelity, such as elevated indel burdens compared to SBS_D-negative samples, they lack the characteristic phenotypes of repair-deficient tumors and pathogenic mutations in known DNA repair genes. The consistency of SBS_D across multiple CRC cohorts and the lack of evidence of DNA repair-deficiency in SBS_D-positive

samples support its classification as a distinct signature, potentially associated with DNA repair infidelity, rather than a composite of known repair-deficiency signatures…"

Comment: 5. SBS_D in Figure 1 seems different than SBS_D in supplementary figure 1, more specifically the C>T component, why is this? Different cohorts?

Response: We appreciate the reviewer's observation. SBS_D does appear different between **Figure 1** and **Supplementary Figure 1**; this reflects differences in the mutational context used for extraction. We have provided all three plots as part of the response in **Response Figure R1.3** on the next page. Importantly, all extractions were performed on the same Mutographs CRC dataset.

- **Figure 1 (SBS-288 context; panel *a* in Response Figure R1.3; see next page):** This schema extends the standard SBS-96 classification by incorporating transcriptional strand information (non-transcribed, transcribed, and un-transcribed categories). It provides additional resolution that helps separate overlapping signals.

- **Supplementary Figure 1*a* (SBS-96 context; panel *b* in Response Figure R1.3):** The standard classification with 96 categories based only on the substitution type and trinucleotide context. It is the coarsest schema and may merge features from distinct processes.

- **Supplementary Figure 1*b* (SBS-1536 context; panel *c* in Response Figure R1.3**): This schema incorporates the full pentanucleotide sequence context, resulting in 1,536 categories. It provides the highest resolution and most detailed separation of signatures.

Because these schemas differ in resolution, the exact profile of a given *de nov*o signature (such as SBS_D) can look slightly different when collapsed back to SBS-96. Higher-resolution schemas (SBS-288, SBS-1536) are better able to disentangle overlapping processes, whereas SBS-96 may blur some features, leading to modest differences, such as those observed in the C>T component.

a

c

b

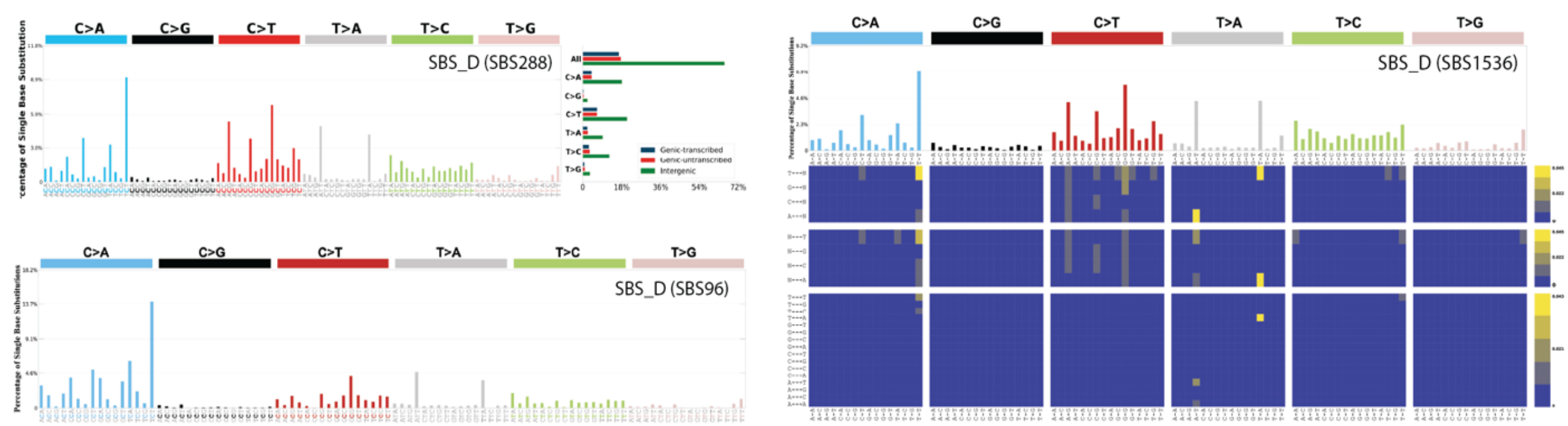

Response Figure R1.3: SBS_D-like signatures extracted using different mutational contexts in the Mutographs CRC cohort. ***(a)*** SBS_D from the original Mutographs CRC study, extracted using the SBS-288 context. ***(b)*** SBS_D-like signature extracted from the Mutographs CRC cohort using the SBS-96 context. ***(c)*** SBS_D-like signature extracted from the Mutographs CRC cohort using the SBS-1536 context.

Color/Font Legend: Reviewers' Comments, Authors' Responses, *Revised Manuscript Excerpts*

Reviewer #2 (Remarks to the Author):

Thank you for the opportunity to review the manuscript by Kazachkova et al. The study analysed 802 treatment naïve MSS CRCs from the Mutographs project and identified a de novo signature referred to as SBS_D that was investigated as a novel signature. SBS_D was identified in three additional CRC cohorts using the same approach. Key findings were: 1) decomposition of SBS_D into COSMICv3.4 clocklike, SBS18,and SBS34 signatures, 2) using a decomposition with a restricted set of signatures, the patterns of SBS_D align with signatures previously associated with deficiencies in POLD1 and mismatch repair, 3) evidence that SBS_D represented a late mutational event/process, 4) high indel mutation rate, and 5) topography analysis showing SBS_D mutations were depleted near nucleosomes. The findings are potentially interesting but need further clarity, presentation and interpretation to improve the validity and relevance of the results.

Major concerns

Comment: 1) A more comprehensive argument for the signature's novelty by comparing to the full set of COSMIC 3.4 signatures - does SBS_D match/have strong cosine similarity with any of the Signal signature definitions? Additionally, many comparisons are between SBS_D and SBS18 – a similar comparison to other signatures (particularly SBS10c) may be informative.

Response: We thank the reviewer for this suggestion. We have now compared SBS_D against the complete set of COSMIC v3.4 reference signatures as well as the complete set of Signal signatures (**Response Figure R2.1**). With COSMIC v3.4, SBS_D does not show a cosine similarity above 0.80 with any reference COSMIC signature. With Signal, only one signature, Signal SBS14, reaches a cosine similarity of 0.808. For context, the average expected cosine similarity between two random nonnegative vectors is approximately 0.75, and two signatures are typically considered to represent the same process when the cosine similarity exceeds 0.90. Thus, SBS_D shows no close match to any existing reference signature in either COSMIC or Signal. Prompted by the reviewer's question, we have now explicitly stated this in the updated manuscript.

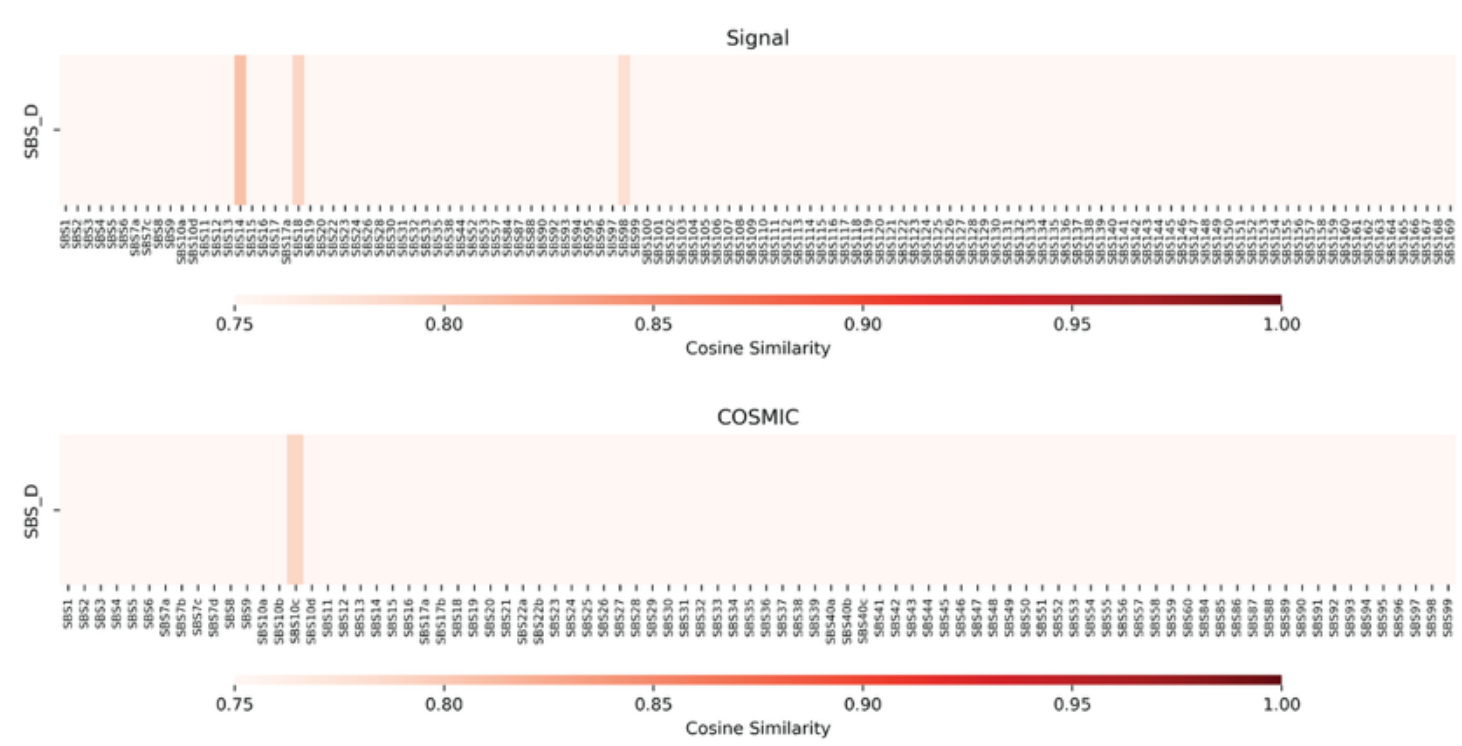

Response Figure R2.1: Comparison Between SBS_D and the COSMIC and Signal Reference Signatures. Cosine similarity between SBS_D and all the Signal mutational signatures (top) and COSMICv3.4 mutational signatures (bottom). Cosine similarity was calculated using the SBS-96 context.

RESULTS

"This consistency across different mutational schemas highlights the robustness of the patterns for these signatures, irrespective of the underlying classification framework. Furthermore, we

observed that SBS_D was dissimilar to any known COSMIC[6] or Signal[19] mutational signatures (cosine similarities < 0.81 across all signatures). Overall, these findings raised doubts about the assignment of SBS18 as a contributor to SBS_D and suggested that SBS_D could represent a distinct mutational process."

Additionally, as the reviewer pointed out, we predominantly focused on comparisons between SBS_D and SBS18. We have now also directly compared the C>A contexts between SBS_D and SBS10c (**Response Figure R2.2**). Indeed, we see a higher cosine similarity between the C>A contexts of SBS_D and SBS10c (cosine similarity of 0.972) than the C>A contexts of SBS_D and SBS18 (cosine similarity of 0.800), supporting our results that SBS10c better explains the C>A contexts of SBS_D.

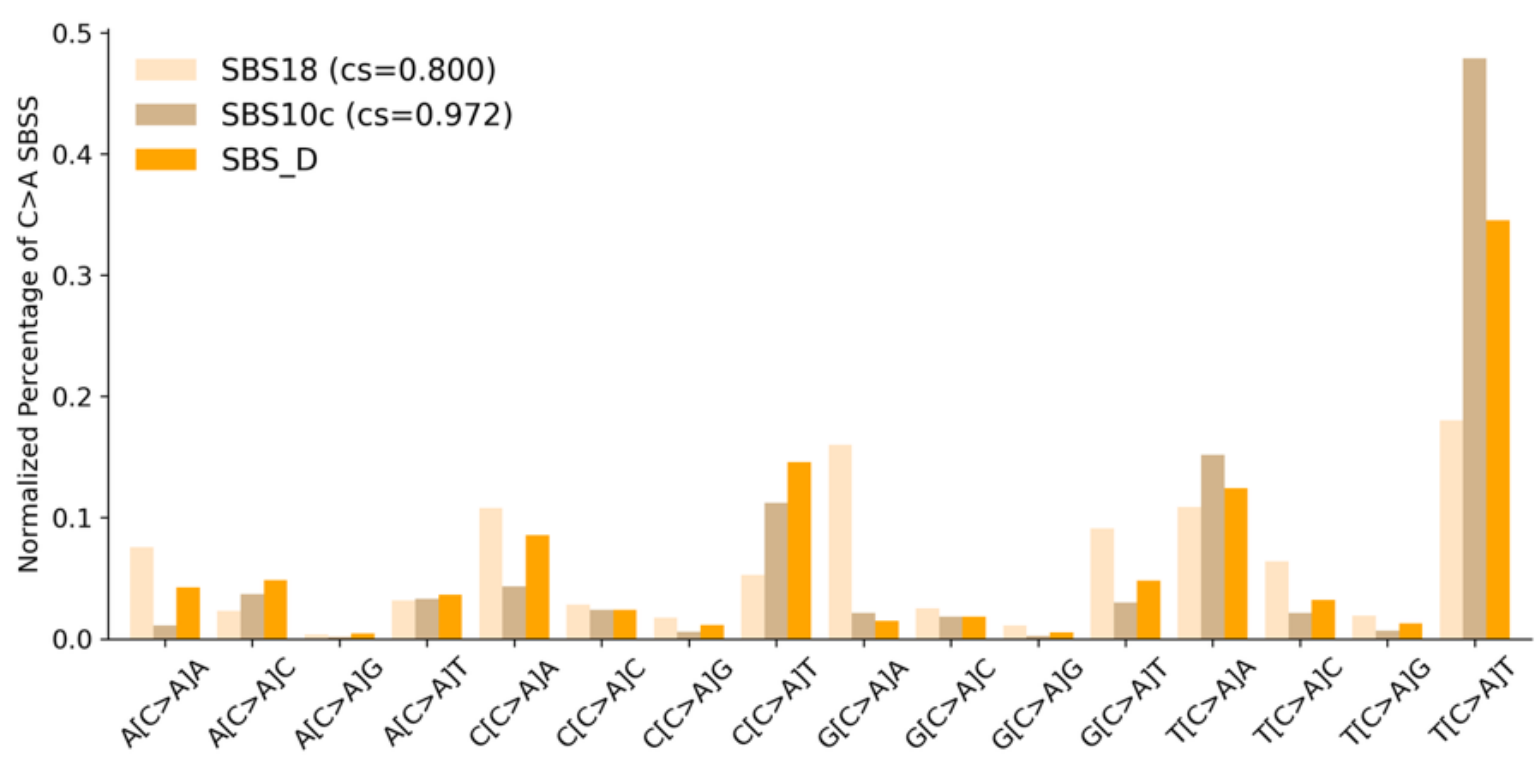

Response Figure R2.2: Comparison of the C>A contexts between SBS_D, SBS10c, and SBS18. Normalized percentage of the C>A contexts for SBS18, SBS10c, and SBS_D. Cosine similarity (cs) relative to SBS_D is denoted for SBS18 and SBS10c and was calculated using the SBS-96 context.

Comment: 2) Does SBS_D co-occur with other signatures, especially SBS18 and SBS10c? A correlation matrix across samples may help establish SBS_D as a distinct process or composite/proxy for other existing signatures.

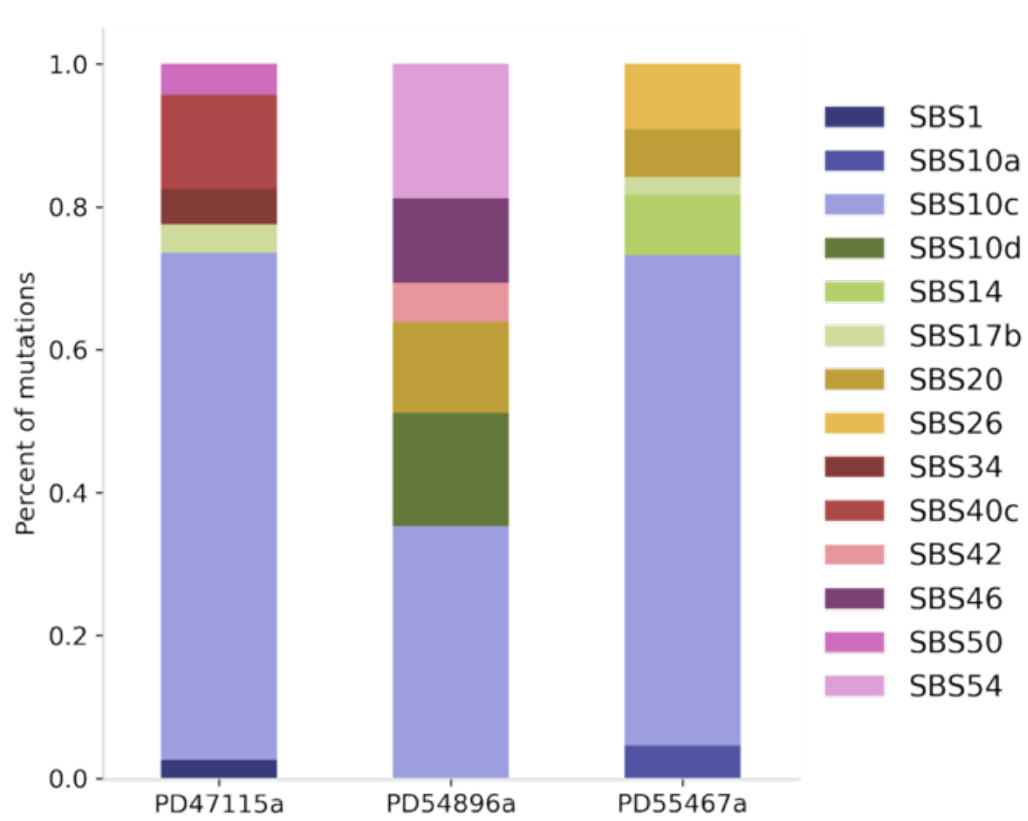

Response Figure R2.3: Signature attribution results in POLD1-deficient samples. Percent of mutations attributed to each mutational signature using Mutational Signature Attribution (MSA) in the 3 CRC samples in the Mutographs cohort with pathogenic *POLD1* mutations. MSA was run using all the COSMICv3.4 signatures as well as SBS_D collapsed to the SBS-96 context.

Response: To address the reviewer's suggestion, we performed additional analyses to assess the relationship of SBS_D with other mutational signatures. It should be noted that SBS_D was not observed in any samples with SBS10c. The only cases with SBS10c were three CRC samples, each harboring a pathogenic POLD1 exonuclease domain mutation, and none showed SBS_D activity (**Response Figure R2.3**).

The reviewer raises an important point about the value of a correlation matrix. If SBS_D were merely a composite or proxy of another signature, we would expect a negative correlation in activity with that signature, since they would directly compete during the assignment process (PMID:

35168570; PMID: 36388765; PMID: 32025018). To evaluate this, we constructed a correlation matrix across all COSMIC-decomposed signatures from DNA repair–proficient samples in the Mutographs CRC cohort (**Response Figure R2.4**). Contrary to this expectation, SBS_D showed positive correlations with SBS18 (Spearman ρ = 0.45) and SBS1 (Spearman ρ = 0.34).

These positive associations may have a biological basis—for example, POLD1-deficient cell lines have been shown to accumulate excess DNA damage under oxidative stress, consistent with unrepaired oxidative lesions in the setting of polymerase malfunction (PMID: 26087769). Because SBS18 is attributed to reactive oxygen species and SBS1 reflects clock-like mutagenesis, it is plausible that tumors with higher SBS18 or SBS1 activity also tend to exhibit SBS_D. However, we note that other pairs of signatures (e.g., SBS93 and SBS_H; SBS8 and SBS17b) display correlations of similar magnitude, suggesting that these relationships could also be spurious rather than reflecting true biological links.

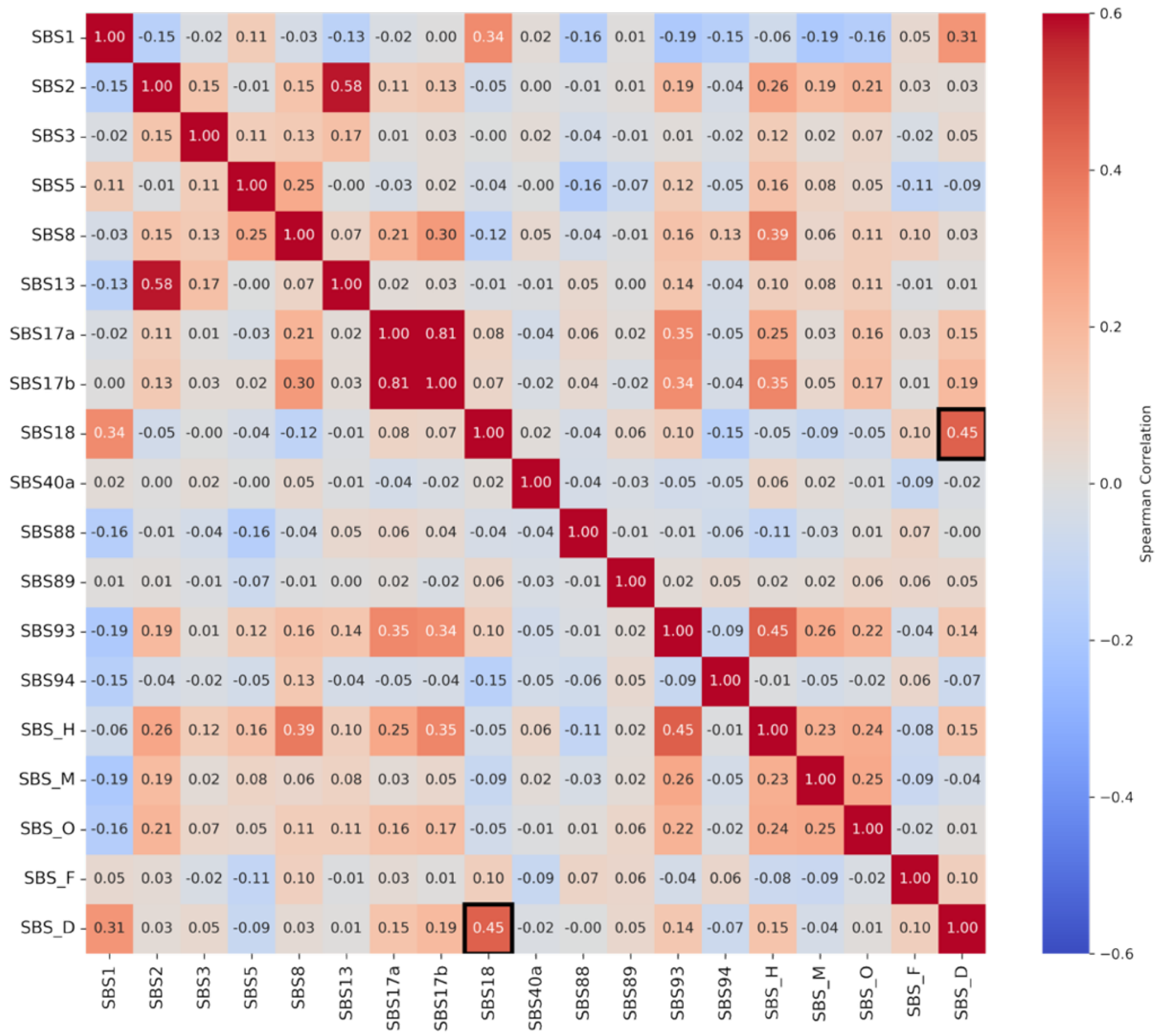

Response Figure R2.4: Correlation Matrix of the Mutational Signatures in the Mutographs CRC Cohort. Spearman correlation across samples for all the COSMIC decomposed mutational signatures in the Mutographs CRC cohort. Black boxes indicate the Spearman correlation between SBS18 and SBS_D.

Comment: 3)It isn't clear why the de novo analysis did not identify SBS_D when dMMR samples were included if SBS_D is not a dMMR related signature? This part of the analysis and results needs further explanation.

Response: We are grateful to the reviewer for the opportunity to clarify this important point. SBS_D bears similarity to SBS10c and SBS15, which are generated by POLD1-deficiency and DNA mismatch repair deficiency, respectively. This similarity is evident in the naïve

decomposition of SBS_D, which yields SBS10c and SBS15. Importantly, however, we do not observe pathogenic *POLD1* mutations, microsatellite instability, or other canonical features of DNA repair-deficiency in SBS_D-positive samples.

The reason SBS_D is not extracted when dMMR or *POLD1*-mutant samples are included is that mutational signature extraction is a signal-separation problem. DNA repair-deficient tumors are hypermutated, carrying an order of magnitude more SBS mutations than repair-proficient tumors, and their profiles are dominated by canonical deficiency signatures such as SBS10c and SBS15. Because SBS_D shares certain features with SBS10c and SBS15, when these high-burden samples are included, the NMF algorithm attempts to resolve the stronger signals from DNA repair deficiency, while the weaker SBS_D signal in repair-proficient tumors is effectively masked and disappears.

We have revised the manuscript text to make this point clearer: the presence of MSI and *POLD1*-mutated samples introduces overwhelming SBS10c and SBS15 signals, and because SBS_D can be partially decomposed into these signatures, NMF does not recover SBS_D as a separate process when such samples are included.

DISCUSSION

"As multiple large-scale colorectal cancer cohorts[14,15] have previously undergone mutational signature analysis, it is surprising that SBS_D has not been reported. A likely explanation is that these studies did not separate DNA repair-deficient and DNA repair-proficient cancers prior to performing de novo signature extraction. DNA repair-deficient tumors typically harbor an order of magnitude more single-base substitutions—approximately 10^5 compared to about 10^4 in microsatellite-stable cases—and are dominated by canonical deficiency signatures such as SBS10c and SBS15. Because SBS_D shares certain features with these repair-deficiency signatures, its signal is masked when hypermutated tumors are analyzed together with repair-proficient cases. This challenge is compounded by the fact that SBS_D usually contributes between 5 to 20% of mutations in SBS_D-positive microsatellite-stable tumors, making it a relatively weak process in mixed cohorts. Thus, when extraction algorithms attempt to resolve the much stronger signals of SBS10c and SBS15, SBS_D is not recovered as a distinct signature."

Comment: 4) It is not clear how the authors define the presence of SBS_D, this should be included and clearly defined. Providing a graph of the proportion of SBS_D out of total signature proportion

of each CRC across the MSS CRCs from each of the cohorts would be informative and demonstrate dominance/contribution of SBS_D to entire mutational processes.

Response: We appreciate the reviewer for pointing this out. In our analysis, the presence of SBS_D was defined as having non-zero activity. We agree that this definition should be stated more clearly in the text, and we have revised the manuscript accordingly.

RESULTS

*"Importantly, SBS_D was found to have non-zero activity in 428/802 microsatellite-stable CRC samples, contributing 8.5% of all mutations (**Figure 1a**).*

…

First, although SBS_D-positive samples, defined as those with non-zero SBS_D activity, lacked the characteristic features of POLD1…"

We further concur with the reviewer that a graph is a helpful way to illustrate the contribution of SBS_D, and we have also applied the reviewer's idea to SBS18^. We have now created this figure, shown on the next page, for both SBS_D and SBS18^ (**Response Figure R2.5**); the figure has also been included in the revised manuscript as **Supplementary Figure 5**. Please note that in answering this comment we also revised a minor typo in **Figure 3*a*** to update the *Total Mutations* value for SBS18^ in the TCGA cohort.

RESULTS

*"Moreover, aside from the GEL cohort—which showed lower signature prevalence—the total number of mutations attributed to each signature, the proportion of samples with non-zero activity of each signature, and the proportion of mutations attributed to each signature within samples were very similar across the cohorts, further supporting their robustness and consistency (**Figure 3a; Supplementary Figure 5**)."*

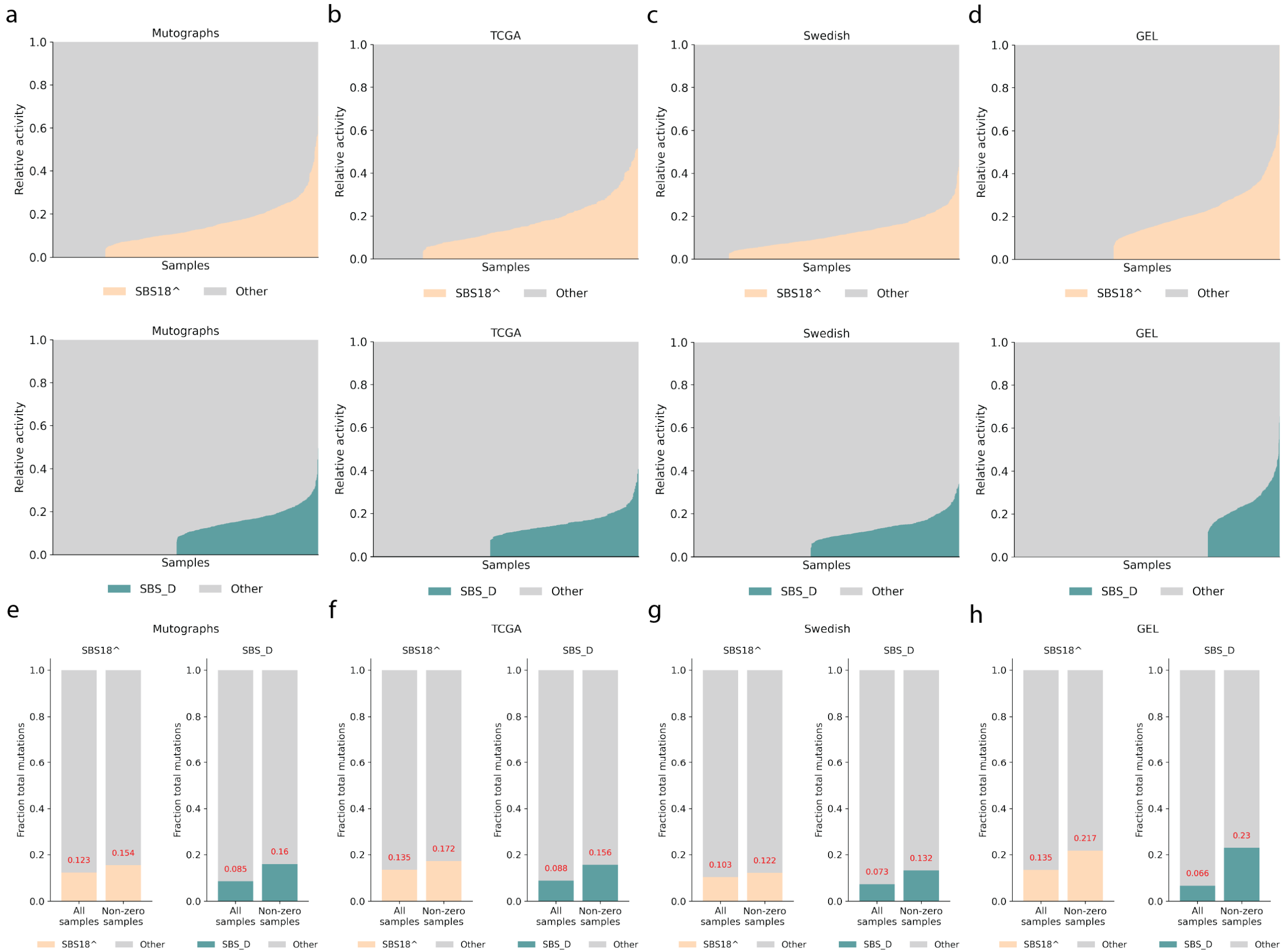

Response Figure R2.5: Proportion of SBS18^ and SBS_D Mutations in Samples Across Four CRC Cohorts. **(a)** Relative activity of SBS18^ (top) and SBS_D (bottom) across samples in the Mutographs cohort as identified by Mutational Signature Attribution (MSA). Samples are sorted in increasing order by the relative activity of the respective signature. **(b)** As in (a), but for the TCGA cohort. **(c)** As in (a), but for the Swedish cohort. **(d)** As in (a), but for the GEL cohort. **(e)** (left) Fraction of SBS18^ mutations out of the total mutations across all samples in the Mutographs cohort as well across only samples with non-zero SBS18^ activity in the Mutographs cohort. Red number denotes the fraction of SBS18^ mutations out of total mutations in the respective group of samples. (right) Fraction of SBS_D mutations out of the total mutations across all samples in the Mutographs cohort as well across only samples with non-zero SBS_D activity in the Mutographs cohort. Red number denotes the fraction of SBS_D mutations out of total mutations in the respective group of samples. **(f)** As in (e), but for the TCGA cohort. **(g)** As in (e), but for the Swedish cohort. **(h)** As in (e), but for the GEL cohort.

Comment: 5) The finding that SBS_D was identified in 428/802 or 53.3% of MSS samples but contributing only 8.5% of all mutations needs further clarity as it suggests that a low threshold was used for considering SBS_D to be present. This would suggest SBS_D is a composite of low-level background signatures and not a dominant mutational process. Discussion of SBS_D in the context of existing highly prevalent signatures (SBS1, SBS5) would help better understand the biological role of SBS_D.

<u>Response:</u> The reviewer is correct that we used a low threshold of more than 0 mutations to define the presence of SBS_D. Importantly, Mutational Signature Attribution (MSA; PMID: 34736398)—the tool used here and in all prior Mutographs studies—generates pruned attributions and reports a signature only if it is present with ≥95% confidence in a sample. Activities that fall below this threshold are set to zero. Thus, samples with non-zero SBS_D activity can be interpreted with high confidence as truly harboring SBS_D.

Across all MSS samples, SBS_D contributed 8.5% of mutations. However, in SBS_D-positive samples (non-zero activity), SBS_D accounted for 16% of mutations (764,896/4,784,837). We agree with the reviewer that presenting only the overall 8.5% could suggest SBS_D is a low-level process. To address this, we have revised the text to also highlight its contribution within SBS_D-positive tumors, where it represents a substantial mutational burden.

In the Mutographs CRC cohort, SBS_D is the fourth most prevalent of the 16 *de novo* signatures identified (**Response Figure R2.6**). Only SBS_A (SBS1+SBS5), SBS_B (SBS18-like), and SBS_C (SBS8+SBS40a) contribute more mutations. SBS_D exceeds the contributions of other CRC-relevant but less common signatures such as SBS17b (SBS_G) and SBS93 (SBS_I). This ranking aligns with its molecular timing: ubiquitous lifelong processes (SBS1, SBS5, and SBS18) accumulate the largest burdens, while late clonal processes like SBS_D contribute fewer overall mutations but remain robustly detectable. We have updated the manuscript text accordingly.

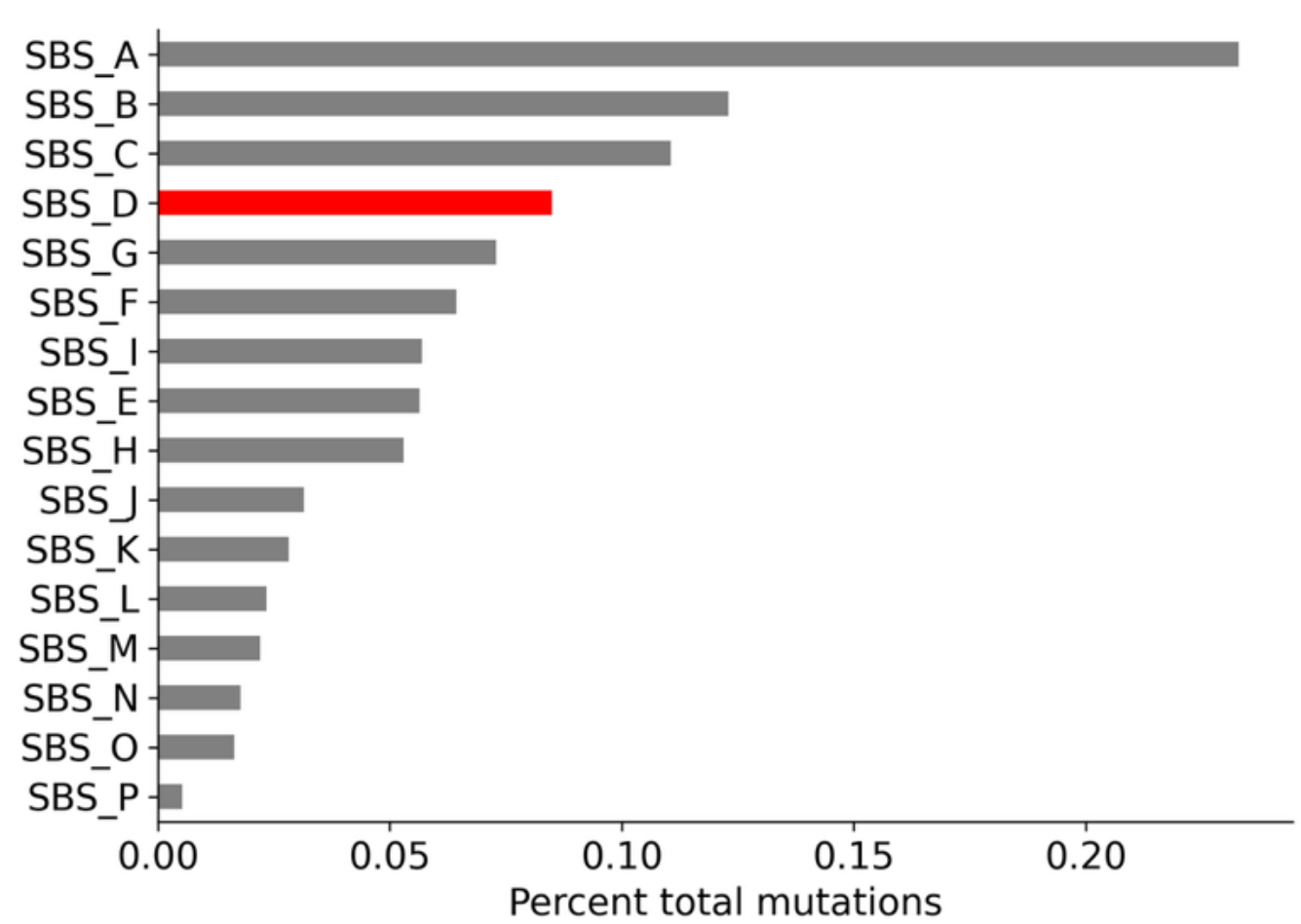

Response Figure R2.6: Contribution of each *de novo* mutational signature in the Mutographs CRC cohort. Percent of the total mutations attributed to each of the *de novo* mutational signatures using Mutational Signature Attribution (MSA). Signatures are sorted by the percent of total mutations that they contribute. SBS_D is indicated in red.

RESULTS

*"Importantly, SBS_D was found to have non-zero activity in 428/802 microsatellite-stable CRC samples, contributing 8.5% of all mutations across the 802 samples (**Figure 1a**). Among the 16 de novo mutational signatures identified in the Mutographs CRC cohort, SBS_D ranked fourth in total mutational contribution. Additionally, in the 428 samples with non-zero SBS_D activity,*

SBS_D contributed 16% of all mutations. Thus, although it contributes fewer mutations than the most ubiquitous CRC processes, SBS_D still represents a major contributor to mutagenesis in DNA repair-proficient CRC samples."

Comment: 6) The manuscript does not explore associations with clinical or pathological features. The relevance of the SBS_D signature could be further explored given the data available from the CRC cohorts included. Is there an association with age at diagnosis, tumor anatomical site, stage and other mutations, pathways etc needs to be included to provide some further resolution of this signature.

Response: We thank the reviewer for this excellent suggestion. We performed association analyses using metadata, where available, from the Mutographs, TCGA, and Swedish cohorts. No consistent relationships were observed between SBS_D activity and age at diagnosis, tumor anatomical site, stage, grade, sex, purity, or driver gene mutations. Additionally, in the Mutographs cohort (where country of residence was available across patients), no association was observed between SBS_D and country. These results are presented on the next page in **Response Figure R2.7**, which has also been included in the manuscript as **Supplementary Figure 6**. Analyses could not be performed for the GEL cohort due to the lack of available metadata. Our results suggest that SBS_D is not associated with any clinical or pathological features. We have now modified the *Results* section and included a Supplementary Figure to summarize these negative results in the revised manuscript.

RESULTS

*"Of note, no consistent associations were observed between SBS_D activity and any standard clinical or pathological features, such as age of diagnosis, tumor subsite, or tumor stage (**Supplementary Figure 6a-c**), nor were mutations in any previously identified driver genes[9] associated with SBS_D (**Supplementary Figure 6d**)."*

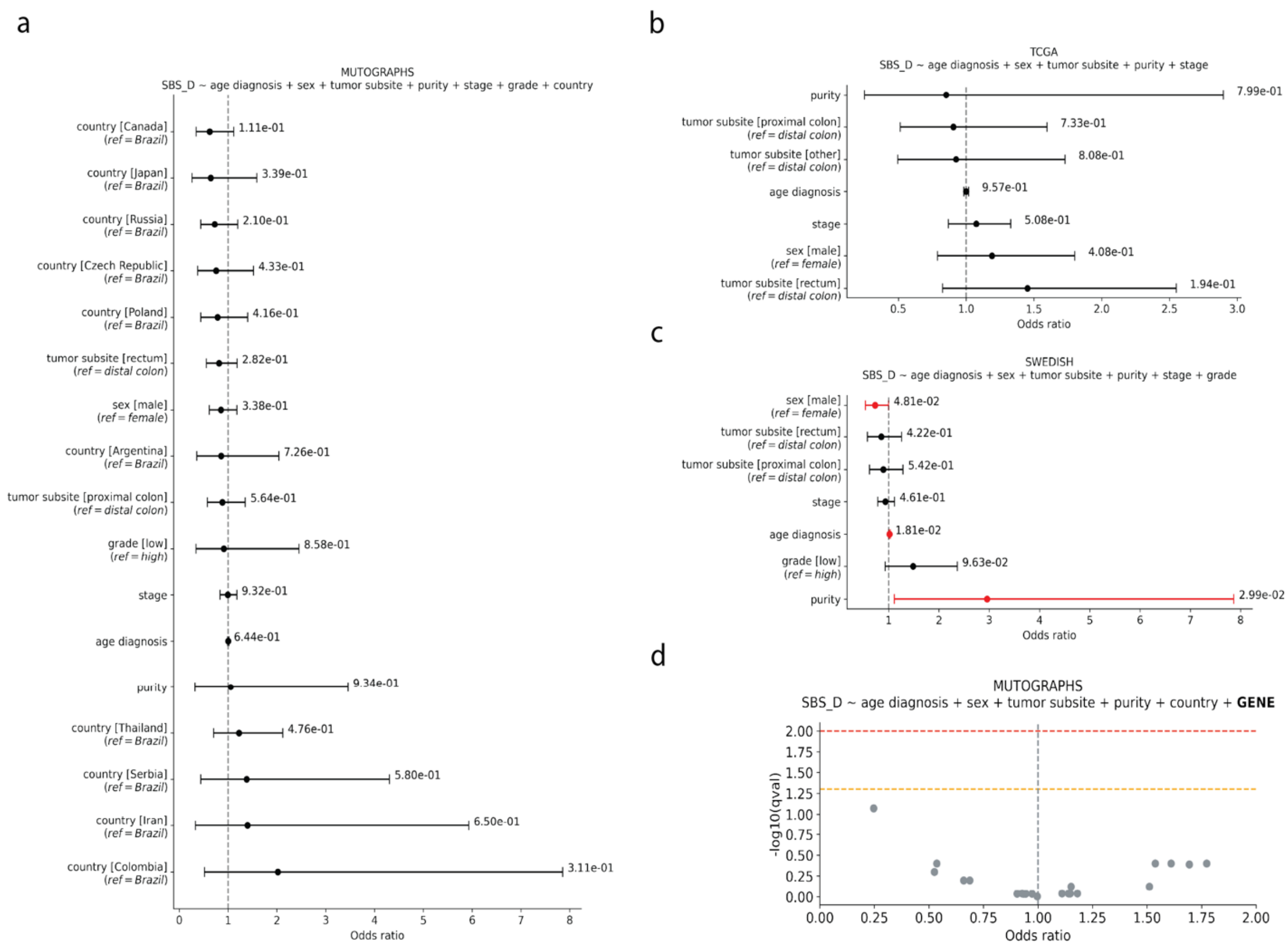

Response Figure R2.7: Associations between SBS_D activity and clinical and pathological features. ***(a)*** Results from a logistic regression model for the Mutographs colorectal cancer (CRC) cohort with SBS_D status as the outcome variable and age of diagnosis, sex, tumor subsite, purity, stage, grade, and country included as covariates. Numbers correspond to p-values for each covariate. Whiskers for each covariate represent the 95% confidence interval. ***(b)*** As in *(a)*, but for the TCGA CRC cohort with age of diagnosis, sex, tumor subsite, purity, and stage as covariates. ***(c)*** as in *(a)*, but for the Swedish CRC cohort with age of diagnosis, sex, tumor subsite, purity, stage, and grade as covariates. Covariates with p-values < 0.05 are indicated in red. ***(d)*** Volcano plot for the Mutographs CRC cohort showing the association between each driver gene mutated in at least 20/802 samples and SBS_D. One logistic regression model was run per gene, with SBS_D as the outcome variable and age of diagnosis, sex, tumor subsite, purity, and the status of the respective gene (mutated versus not mutated) as covariates. P-values for each gene were then corrected using the Benjamini-Hochberg Procedure. The orange line represents a q-value of 0.05, and the red line represents a q-value of 0.01.

Comment: 7) Further investigation of other DNA repair genes may be useful to determine if there is an association with SBS_D outside of *POLD1* and DNA mismatch repair genes. This may be particularly relevant given its cosine similarity to SBS18, and the observation of high indel mutation numbers.

Response: We thank the reviewer for this suggestion and have now performed a comprehensive analysis of over 220 human DNA repair genes based on Knijnenburget *et al.* (PMID: 29617664).

Using annotations from Variant Effect Predictor (VEP) in the Mutographs CRC cohort, we first examined single nucleotide variants (SNVs), indels, or both in these genes. Logistic regression models—with age, sex, tumor subsite, purity, and country included as covariates—showed no association between SBS_D activity and the presence of SNVs (717/802 samples), indels (145/802 samples), or either SNVs or indels (746/802 samples) in DNA repair genes. Likewise, no relationship was found between SBS_D activity and the number of DNA repair genes mutated per sample (**Response Figure R2.8*a***).

We then tested each DNA repair gene mutated in at least 10 samples (25 genes total) individually using logistic regression with the same covariates. Again, no associations were identified (**Response Figure R2.8*b***).

Together, these analyses indicate that SBS_D activity is not associated with mutations in known DNA repair genes, supporting our conclusion that SBS_D does not reflect canonical defects in DNA repair pathways.

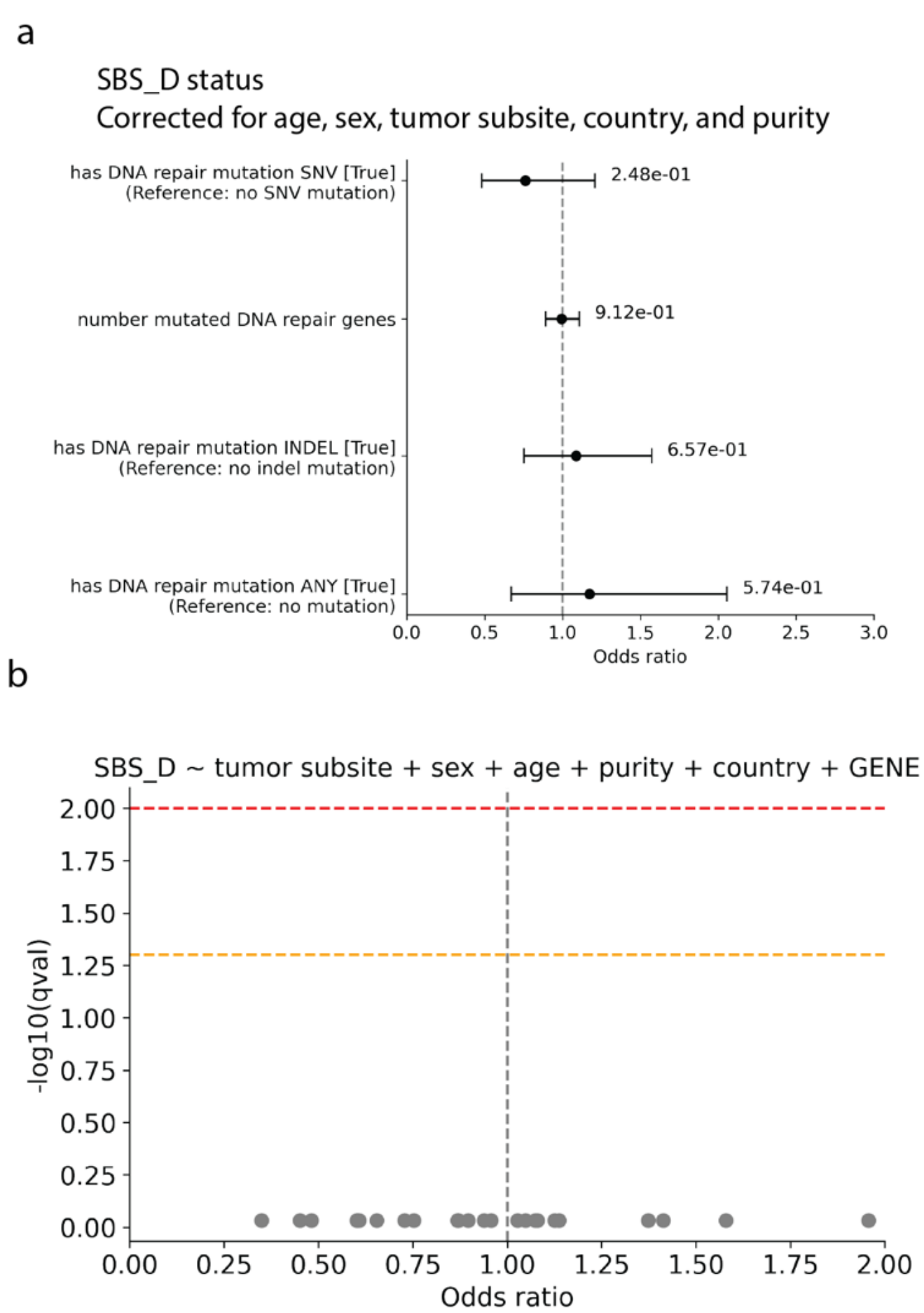

Response Figure R2.8: Associations between SBS_D and mutations in known DNA repair genes in the Mutographs CRC cohort. *(a)* Results from four logistic regression models with SBS_D as the outcome variable and age, sex, tumor subsite, country, and purity as covariates, as well as each of the listed DNA repair mutation conditions: any mutation (single nucleotide variant [SNV] or indel) in a DNA repair gene, indel mutation in a DNA repair gene, SNV in a DNA repair gene, and the total number of mutated DNA repair genes via either single nucleotide variant or indel. Numbers represent the p-value for the respective covariate. ***(b)*** Volcano plot from logistic regression models with SBS_D as the outcome variable and tumor subsite, sex, age, country, purity, and the mutation status of the 25 DNA repair genes mutated, either via single nucleotide variant or indel, in at least 10/802 CRC samples. P-values were corrected across the 25 genes using the Benjamini-Hochberg procedure. The orange line represents a q-value of 0.05, and the red line represents a q-value of 0.01.

Comment: 8) Are the indel mutations early/late events which may help establish how the elevated indel counts link to SBS_D.
Response: We appreciate the reviewer's suggestion. However, molecular timing cannot be used to directly compare the absolute number of early versus late clonal indels across samples, since timing depends on copy number changes: samples with few or no copy number alterations will naturally yield fewer annotated events than those with many. Thus, comparing raw counts of early and late clonal indels between SBS_D-positive and SBS_D-negative samples is not feasible.

Instead, we examined the relative activity of indel signatures in the early clonal and late clonal mutations, which allows comparisons independent of total indel counts. Focusing on ID1—which was found to be highly correlated with SBS_D activity in our manuscript (Spearman correlation = 0.66; q-value < 0.001) and is known to be elevated in POLD1-deficient tumors—we found only minor differences between SBS_D-positive and SBS_D-negative samples in early clonal indels, but a more pronounced difference in late clonal indels. This pattern further supports a potential link between SBS_D and elevated ID1 activity in SBS_D-positive tumors (**Response Figure R2.9**).

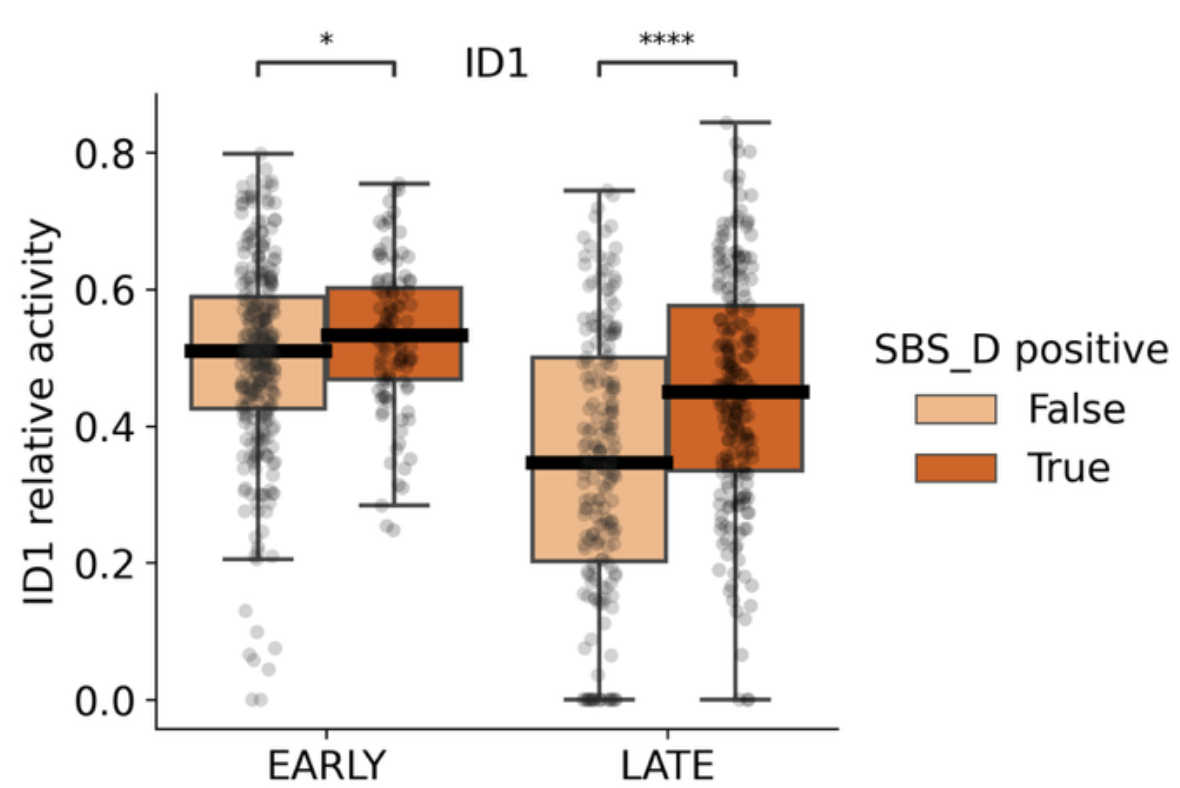

Response Figure R2.9: Relative activity of ID1 in SBS_D-positive and SBS_D-negative samples at different molecular timings. Comparison of the relative activity of ID1 in early clonal mutations (EARLY) and late clonal mutations (LATE) between samples that have no SBS_D activity and samples that have non-zero SBS_D activity in the early clonal and late clonal mutations, respectively. Significance was assessed using a Mann Whitney U Test.

Comment: 9) The Figure legends lack important detail throughout and should be improved.
Response: Thank you for bringing this to our attention. The figure legends have been revised to include additional details.

Comment: 10) The discussion needs to include text on the interpretation of the results for nucleosome and topography and what this means, particularly in the context of other signatures to better understand how significant the SBS_D result is – comparing the results to other known signatures may aid interpretation regarding nucleosome occupancy.
Response: We concur with the reviewer's comment, and we have now added text addressing nucleosome occupancy and topography in both the *Results* and the *Discussion* sections. Relevant excerpts are provided on the following page.

RESULTS

*"Specifically, SBS_D mutations were depleted near nucleosomes and did not exhibit any periodicity. In contrast, SBS18^ mutations were enriched near nucleosomes and demonstrated clear periodicity as expected and previously reported[22] for COSMIC signature SBS18 in CRC and across all cancer types (**Figure 2d**). Of note, strong depletion at nucleosome-bound regions is a relatively rare behavior for single base substitution mutational signatures, with only 11/48 previously examined single base substitution signatures displaying this pattern[22]. These observations further highlight the clear similarity between SBS18^ and SBS18, and they suggest that SBS_D represents a mutational process dissimilar to the one captured by SBS18."*

DISCUSSION

"In this study, we revisited the Mutographs CRC mutational signature analysis[9], where mutational signature SBS_D was previously decomposed into the reactive oxygen species-associated signature SBS18. Using molecular timing and topography analysis, which has previously been used to distinguish between two mutational processes that presented as similar mutational signatures[22], we demonstrated that SBS_D was a late clonal signature enriched in linker-DNA, which is in direct contrast to the molecularly early/consistent and enriched at nucleosomes behavior observed for SBS18."

Comment: 11) There is some redundancy in text related to describing how signatures are measured. The first paragraph of the results is largely duplicated with the text in the methods section. The description in the results should be moved and merged with the text in the methods. This will allow inclusion of text in the discussion on nucleosome and topography results as suggested above.

Response: We apologize for this oversight. We agree with the reviewer and have revised the manuscript accordingly by merging the redundant description from the *Results* into the *Methods* section.

Comment: 12) The similarity between SBS_D and SBS10c is present only after removal of signatures in the naïve analysis. Is removing signatures introducing bias by forcing mutations to be assigned to less relevant signatures? This should be addressed by the authors.

Response: We are grateful to the reviewer for bringing up this point, as it allows us to clarify the role of naïve analysis versus optimized decomposition with signature exclusion. In the optimized decomposition used in the original Mutographs CRC study, signatures linked to DNA repair-

deficiency were excluded, since all DNA repair-deficient CRC samples had already been filtered out. Under this approach, SBS_D was decomposed into SBS18, SBS34, and the clock-like signatures with a borderline cosine similarity of 0.90. By contrast, in the naïve decomposition, no signatures from the COSMIC reference set were excluded. In this unbiased setting, SBS_D decomposes into SBS10c, SBS15, SBS34, and the clock-like signatures with cosine similarity of 0.98. Thus, the similarity between SBS_D and SBS10c arises specifically from the naïve, unbiased analysis. We have now extended the *Introduction* and *Results* sections of the manuscript to clarify this point.

INTRODUCTION

"Given the extensive catalog of known reference signatures, the decomposition process can be optimized to prioritize biologically relevant signatures, minimizing the risk of overfitting[9-11]. For example, a mutational signature associated with ultraviolet (UV) light exposure would typically be excluded from the decomposition of de novo signatures detected in colorectal or brain tumors as UV-induced mutations are not expected in these contexts[5]. This process of removing specific mutational signatures during decomposition based on prior biological knowledge is referred to as an optimized decomposition approach. This is in contrast with a naive decomposition approach, where no COSMIC signatures are excluded while performing decomposition."

RESULTS

"Specifically, since the analyzed samples were treatment-naïve, microsatellite-stable, and DNA repair-proficient, the optimized decomposition process was designed to exclude mutational signatures associated with chemotherapy exposure or DNA repair deficiencies. In the naïve decomposition approach, which does not exclude any signatures and allows all COSMIC signatures to be utilized in the decomposition, SBS_D decomposed to a mixture of SBS10c (44%), SBS15 (12%), SBS34 (7%), and the clock-like signatures (37%)…"

<u>Comment:</u> 13) Is the application of the same signature methodology in the additional 3 cohorts really a validation of the SBS_D signature or just forcing the same outcome? [is there any overlap in these datasets]

<u>Response:</u> We thank the reviewer for raising this interesting point. The extractions performed in the three additional cohorts represent a true validation of SBS_D, as these datasets are entirely independent with no overlapping samples. We applied the same methodology (SigProfilerExtractor) used in the Mutographs CRC study, and in all three cohorts we consistently

identified an SBS_D-like *de novo* signature. We have now clarified in the manuscript that there is no overlap between the samples.

RESULTS

*"…and (iii) 1,532 samples from the Genomics England (GEL) project, representing patients from the United Kingdom (**Methods**). We note that no overlapping samples were present among any of these cohorts."*

To confirm that this result is not specific to SigProfilerExtractor, we have now also applied two alternative *de novo* extraction methods—MuSiCal (PMID: 38361034) and SignatureToolsLib (PMID: 35949260)—which recovered the same SBS_D-like signature included in the manuscript. The results are shown as **Response Figure R2.10** and included in the manuscript as **Supplementary Figure 2**. These findings strengthen our conclusion that SBS_D is a reproducible and distinct signature rather than an artifact of a particular tool or dataset. Please note that in answering this comment we corrected a small typo in **Supplementary Figure 1*c*** to change SBS D to SBS_D.

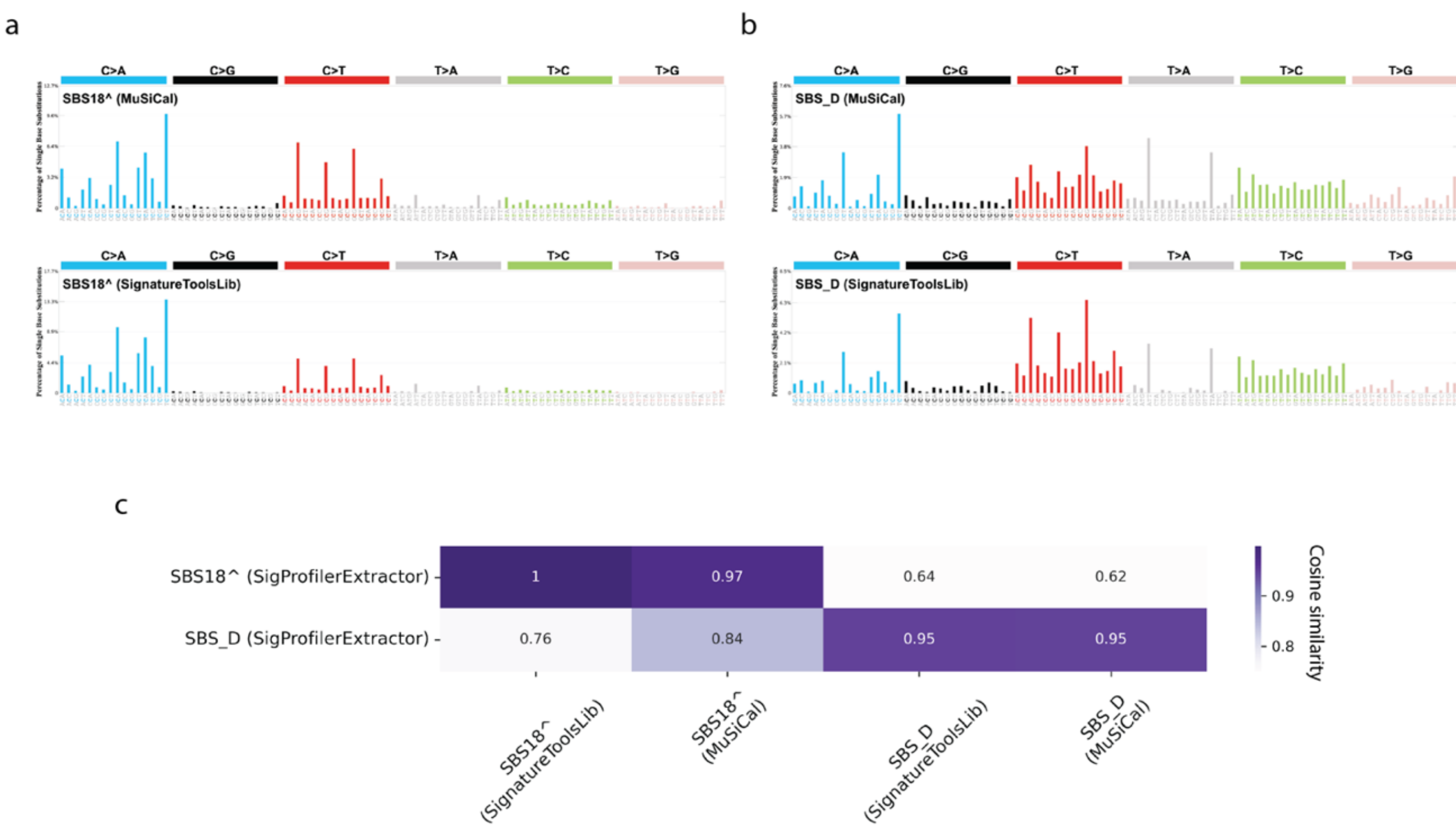

Response Figure R2.10: SBS18^ and SBS_D are extracted from the Mutographs CRC cohort using independent extraction tools. ***(a)*** SBS18^-like signature extracted from the Mutographs CRC cohort using MuSiCal (top) and SignatureToolsLib (bottom). ***(b)*** SBS_D-like signature extracted from the Mutographs CRC cohort using MuSiCal (top) and SignatureToolsLib (bottom). ***(c)*** Cosine similarities between SBS18^ and SBS_D extracted from the Mutographs CRC cohort using SigProfilerExtractor and the SBS18^-like and SBS_D-like signatures extracted from the Mutographs CRC cohort using MuSiCal and SignatureToolsLib. Cosine similarities were calculated using the SBS-96 context for all signatures.

RESULTS

*"Both approaches yielded two signatures closely resembling SBS18^ and SBS_D (**Supplementary Figure 1**). Furthermore, analyses with two independent signature extraction tools—MuSiCal[17] and SignatureToolsLib[18]—also identified signatures highly similar to SBS18^ and SBS_D (**Supplementary Figure 2**). This consistency across different mutational schemas and different extraction tools further highlights the robustness of the patterns for these signatures…"*

Comment: 14) Why do the authors think SBS_D was lower in GEL compared with the other cohorts? Is this a significant difference? Can this be quantified?

Response: The reviewer brings up a good question regarding GEL. SBS_D prevalence was about two times lower in GEL compared to all the other examined CRC cohorts, and this difference is statistically significant (p-values = 3.67e-9, 6.43e-11, and 6.01e-11 for Mutographs, TCGA, and Swedish cohorts, respectively; Binomial Tests). Because only the SBS-96 mutational matrix is publicly available for the GEL cohort (no VCF files or metadata), it is difficult to pinpoint what might be driving the differences observed for GEL. We hypothesize that this difference could be related to several factors about the GEL cohort, including potentially the use of the SBS-96 context, which makes the signal between signatures more difficult to separate, and using only a single mutation caller (i.e., Strelka) rather than a consensus approach, which was used for the Mutographs and Swedish cohorts. Despite the stated differences of the GEL cohort, we are still able to extract an SBS_D-like signature closely resembling the SBS_D-like signatures extracted from the other CRC cohorts (cosine similarity > 0.90 across all cohorts).

Comment: 15) The graph shows lower single base and indel burden for MSS compared with MSI and POLD1 CRCs but the fraction of genome aberrant is higher. Can the authors please provide some clarity on how this derived, and intereptation of this result. the text also says higher indels??

Response: We appreciate the reviewer's question. The percentage of genome aberrated is a commonly used measure that quantifies the fraction of the genome affected by copy number alterations, adjusted for whole-genome duplication. Our results show that microsatellite unstable and POLD1-deficient tumors have fewer copy number alterations than microsatellite-stable CRCs, despite having markedly higher single base substitution and indel burdens. This well-documented phenomenon (e.g., PMID: 8261392 and PMID: 40680851) likely reflects the fact that DNA repair-deficient tumors accumulate such high mutation rates that additional copy number changes are not essential for cancer development and progression. We have revised the text to highlight that microsatellite unstable and POLD1-deficient samples characteristically exhibit elevated

substitution and indel loads but lower levels of genome aberration compared to DNA repair-proficient CRCs.

RESULTS

*"Third, tumors with pathogenic POLD1 mutations or microsatellite instability typically have stable, diploid genomes with aberrations affecting less than 20% of the genome, which is in stark contrast to the substantial genomic instability observed in most tumors with SBS_D activity (**Figure 4b**). In addition to our observations in the Mutographs CRC cohort, previous studies[24,25] have also noted that microsatellite unstable samples and samples with proofreading deficiencies have higher single base substitution and indel burdens but lower genome aberration compared to DNA repair-proficient tumors."*

Comment: 16) Given the high indel count, could SBS_D be related to EMAST?

Response: We thank the reviewer for raising this interesting possibility. Based on current evidence, SBS_D activity does not appear to follow the patterns typically reported for EMAST. Prior studies (PMID: 25836926) suggest that EMAST-positive CRCs are less prevalent in the rectum, occur more often in advanced-stage disease, and are associated with poor differentiation, whereas we observed no consistent association between SBS_D and tumor subsite, stage, or grade (as detailed in our response to point 6).

Importantly, EMAST has been mechanistically linked to deficiency or mislocalization of the mismatch repair protein MSH3. In a previously published study investigating the mutational landscape of MSH3-deficient colorectal adenomas (PMID: 34843512), the authors observed that the mutational profiles of these adenomas were primarily composed of C>T mutations, attributed to SBS1 (clock-like) and SBS6 (dMMR), with no evidence of the prominent SBS10c-like C>A component that characterizes SBS_D. Consistent with this, we found no evidence of MSH3-deficiency in SBS_D-positive samples in the Mutographs CRC cohort, further supporting that SBS_D is likely not related to EMAST.

Color/Font Legend: Reviewers' Comments, Authors' Responses, *Revised Manuscript Excerpts*

Reviewer #1 (Remarks to the Author):

The authors have comprehensively dealt with my points and I recommend the manuscript for publication in Nature communications

Response: We thank the reviewer for the positive evaluation and for recommending our manuscript for publication in *Nature Communications*. We appreciate the time and constructive feedback provided during the review process.

Reviewer #4 (Remarks to the Author):

Most previous comments from Reviewer 2 have been appropriately addressed in the revised manuscript. There are some remaining issues that could further improve the study.

Response: We thank Reviewer #4 for carefully evaluating our prior responses to Reviewer #2 and for noting that the previous comments have been appropriately addressed, which we greatly appreciate. We also thank the reviewer for the additional insightful comments, all of which we have addressed through point-by-point responses and corresponding revisions that have further strengthened the manuscript.

Comment: 1) Previous comments 2, 6, and 7: explore associations of SBS_D with other features, including mutational signatures (comment 2), clinicopathological features and driver mutations (comments 6 and 7). The authors have mostly addressed these comments, but the additional data have augmented the limitation that signature SBS_D seems to be an independent mutational process with no relevant associations and an unclear biological role and relevance. Pathway analyses were proposed and might be helpful in detecting phenotypic consequences but were not included in the revision. Corresponding RNA-seq data are available in at least some of the cohorts and should be included for such analyses.

Response: We thank the reviewer for this thoughtful suggestion and for noting that our analyses indicate SBS_D represents an independent mutational process. Indeed, a major challenge in the field of mutational signature analysis is determining the underlying etiology of signatures whose origins remain unknown.

In response, we have now comprehensively analyzed RNA-seq data from the two cohorts for which such data were available: the Swedish and TCGA cohorts. We performed differential gene expression analyses comparing SBS_D- and SBS_D+ samples, while adjusting for relevant covariates including age, sex, tumor subsite, and tumor purity (**Response Figure R4.1*a-b***; see next page). To comprehensively assess gene expression differences between SBS_D- and SBS_D+ samples, and in line with the reviewer's suggestion to examine high-scoring SBS_D+ cases, we performed this analysis in four complementary comparisons for each cohort: all SBS_D- samples

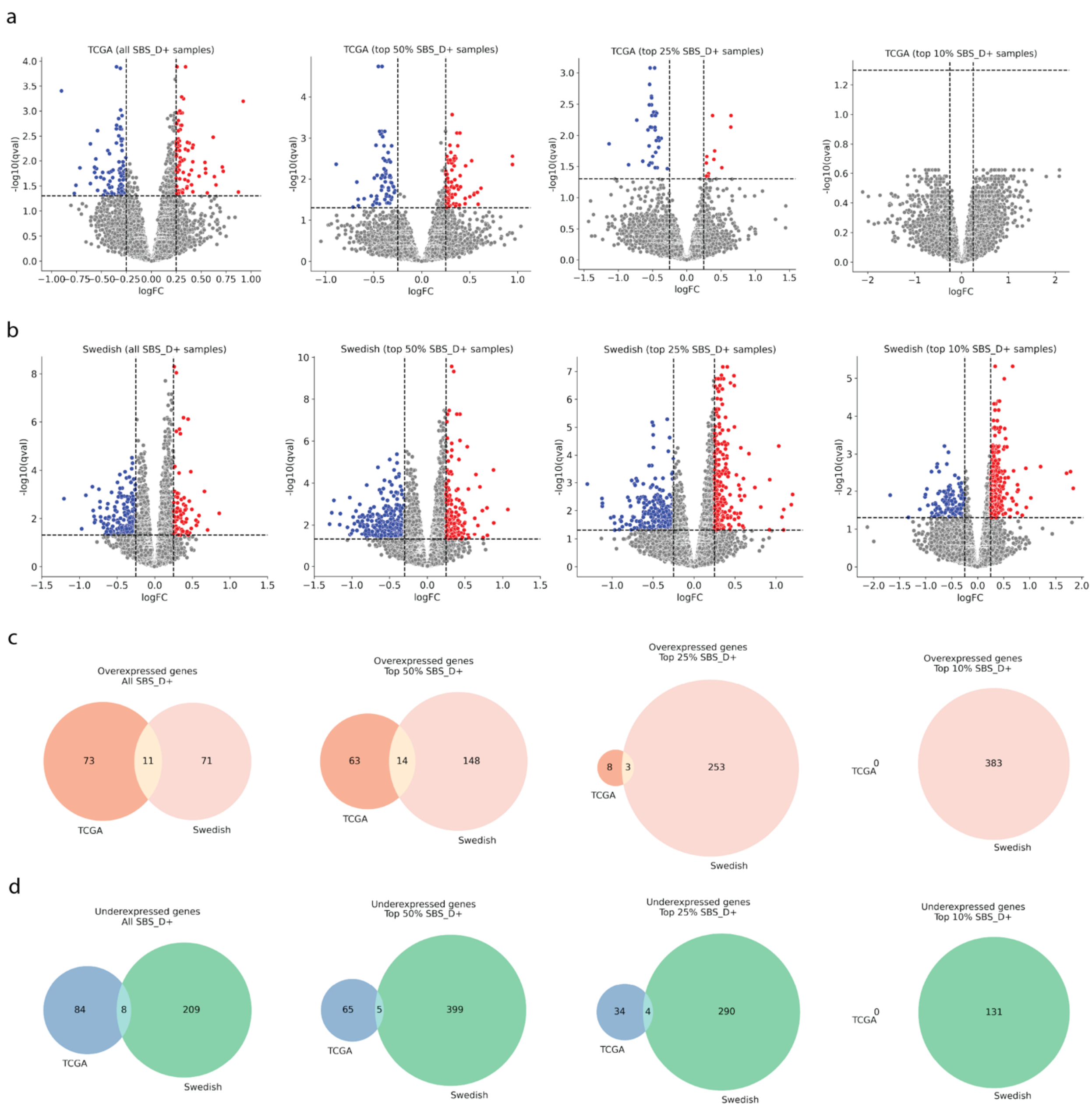

Response Figure 4.1: Differential gene expression analysis between SBS_D+ and SBS_D- samples. ***(a)*** Volcano plots illustrating results from differential gene expression in the TCGA cohort. Analyses were performed using SBS_D+ samples against all SBS_D- samples, the top 50% of SBS_D+ in terms of SBS_D activity against all SBS_D- samples, the top 25% of SBS_D+ samples against all SBS_D- samples, and the top 10% of SBS_D+ samples against all SBS_D- samples; results are shown respectively, starting from the far left. Genes in blue are significantly underexpressed in SBS_D+ samples, while genes in red are significantly overexpressed in SBS_D+ samples. Dashed lines represent the thresholds used for identifying significant differentially expressed genes: a q-value of 0.05 and log2 fold-change (logFC) values of less than -0.25 and greater than 0.25 for underexpressed and overexpressed genes, respectively. ***(b)*** As in *(a)*, but for the Swedish cohort. ***(c)*** Venn-diagram illustrating the overlap of all significantly overexpressed genes identified across all four analyses in (a-b) between the TCGA and Swedish cohorts. ***(d)*** As in *(c)*, but for significantly underexpressed genes.

versus all SBS_D+ samples, and all SBS_D- samples versus the top 50%, 25%, and 10% of

SBS_D+ samples ranked by SBS_D activity. Genes were considered differentially expressed using a false discovery rate of $q < 0.05$ and an absolute log2 fold-change greater than 0.25. More stringent log2 fold-change thresholds substantially reduced the number of significant genes, limiting downstream pathway and enrichment analyses.

The sets of differentially expressed genes for the TCGA and Swedish cohorts are provided in **Supplementary Tables 9-22**; notably, very few genes were consistently overexpressed or underexpressed in both cohorts across our analyses, suggesting a lack of consistent transcriptional alterations associated with SBS_D across datasets (**Response Figure 4.1*c-d*;** see previous page).

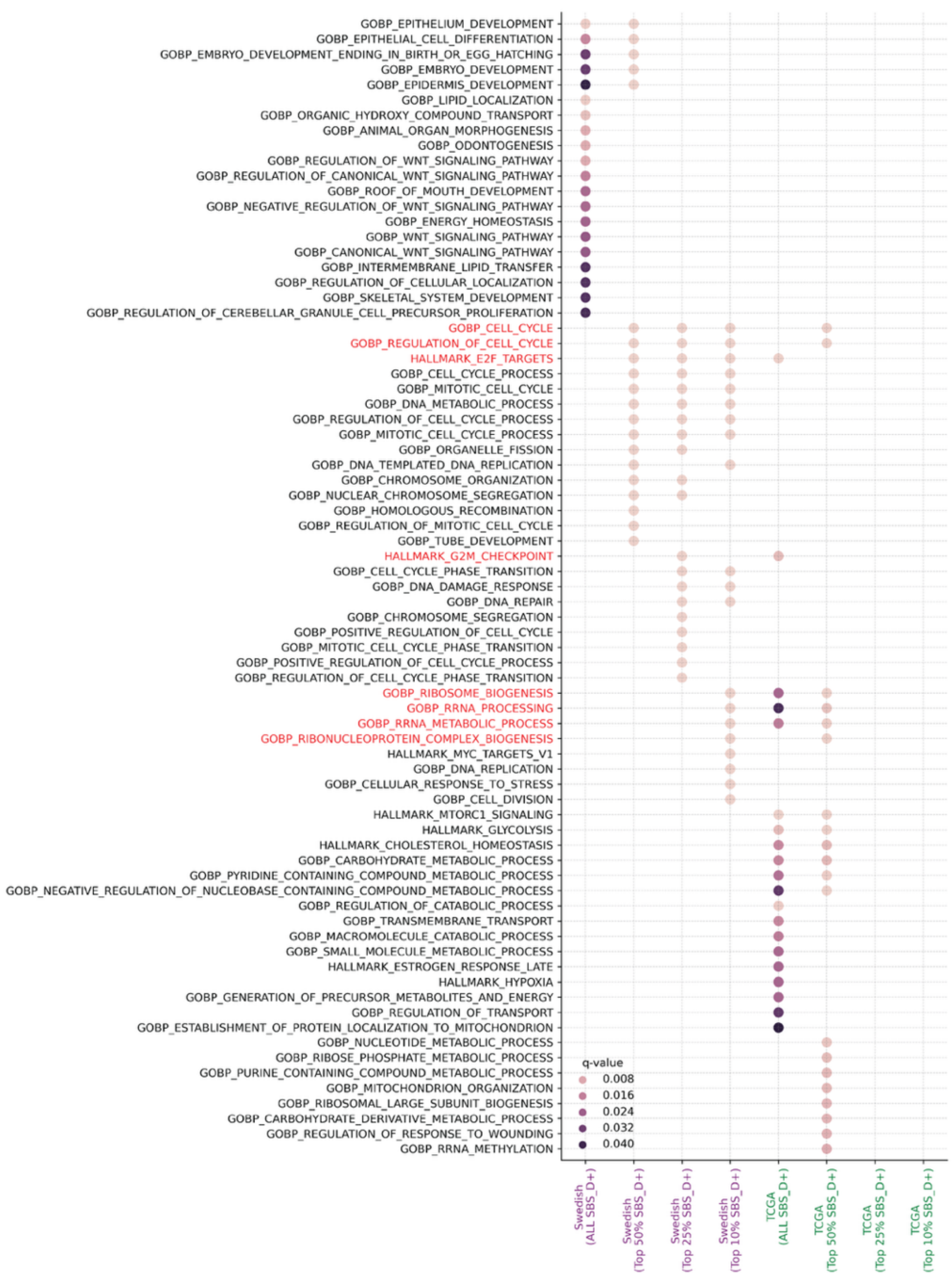

Response Figure 4.2: Pathways enriched in SBS_D+ samples based on overexpressed genes in the Swedish and TCGA cohorts. List of pathways significantly enriched among overexpressed genes, identified by Gene Set Enrichment Analysis (GSEA) in the Swedish (purple; x-axis) and TCGA (green; x-axis) cohorts. Analyses include comparisons of all SBS_D+ samples, as well as the top 50%, 25%, and 10% of SBS_D+ samples, against all SBS_D- samples. Hallmark and GO Biological Process gene sets were used. Each point represents a comparison in which a gene set was significantly enriched among overexpressed genes, with color indicating the corresponding q-value. Gene sets shown in red were consistently enriched in both the Swedish and TCGA cohorts in at least one analysis.

We next performed gene set enrichment analysis to identify pathways significantly enriched among the differentially overexpressed (**Response Figure R4.2**) and underexpressed (**Response Figure**

R4.3) genes. Gene set enrichment analysis was performed using the Hallmark and Gene Ontology (GO) Biological Processes gene sets.

For overexpressed genes, we identified eight pathways that were enriched in SBS_D+ samples across both the Swedish and TCGA cohorts (**Response Figure R4.2**; see previous page). These pathways were primarily related to cell-cycle regulation and biosynthetic processes, suggesting potential alterations in proliferative or biogenetic activity in SBS_D+ samples compared to SBS_D- samples.

In contrast, for underexpressed genes, no pathways were consistently enriched in SBS_D+ samples across both the Swedish and TCGA cohorts. While several pathways were significantly enriched among underexpressed genes within the Swedish cohort, only a single, non-overlapping pathway was identified within TCGA (**Response Figure R4.3**; see next page). Together, these results indicate that SBS_D+ samples do not exhibit consistent or reproducible pathway-level underexpression compared to SBS_D- samples.

Overall, differential gene expression analysis identified eight pathways that were enriched among overexpressed genes in SBS_D+ samples across both the Swedish and TCGA cohorts, whereas no pathways were consistently enriched among underexpressed genes. The enriched pathways were primarily related to cell-cycle regulation and biosynthetic processes. While these findings suggest an association between SBS_D and modest alterations in cell-cycle- and biosynthesis-related transcriptional programs, they should be interpreted with caution given the relatively low log2 fold-change threshold used to define differential expression.

These analyses and the corresponding gene set enrichment results have now been incorporated into the manuscript as **Supplementary Figures 8-10**. In addition, the lists of differentially expressed genes and gene set enrichment results are provided in **Supplementary Tables 9-22**, and the *Results* and *Discussion* sections have been updated accordingly.

RESULTS

*"To further explore the etiology of SBS_D, we performed differential gene expression analysis comparing SBS_D+ and SBS_D- samples in the Swedish and TCGA cohorts with available RNA-seq data (**Methods**; **Supplementary Figure 8**). Gene set enrichment analysis identified eight pathways consistently enriched among overexpressed genes in SBS_D+ samples across both cohorts, primarily related to cell-cycle regulation and biosynthetic processes (**Supplementary Figure 9**; **Supplementary Tables 9-15**). In contrast, no pathways were consistently enriched among underexpressed genes across cohorts (**Supplementary Figure 10**; **Supplementary Tables 16-22**).*

Together, these results indicate reproducible but modest transcriptional differences associated with SBS_D. The limited magnitude of pathway-level perturbations suggests that the observed enrichments should be interpreted cautiously, particularly in the context of the relatively small effect sizes detected."

DISCUSSION

"Overall, despite extensive studies on CRC mutational landscapes, our findings reveal a previously unrecognized mutational signature in DNA repair-proficient CRC. Although RNA expression analyses and associations with clinicopathological features did not yield conclusive insights into SBS_D etiology, our genomic-level analyses suggest that SBS_D may reflect DNA repair infidelity during the late stages of CRC evolution."

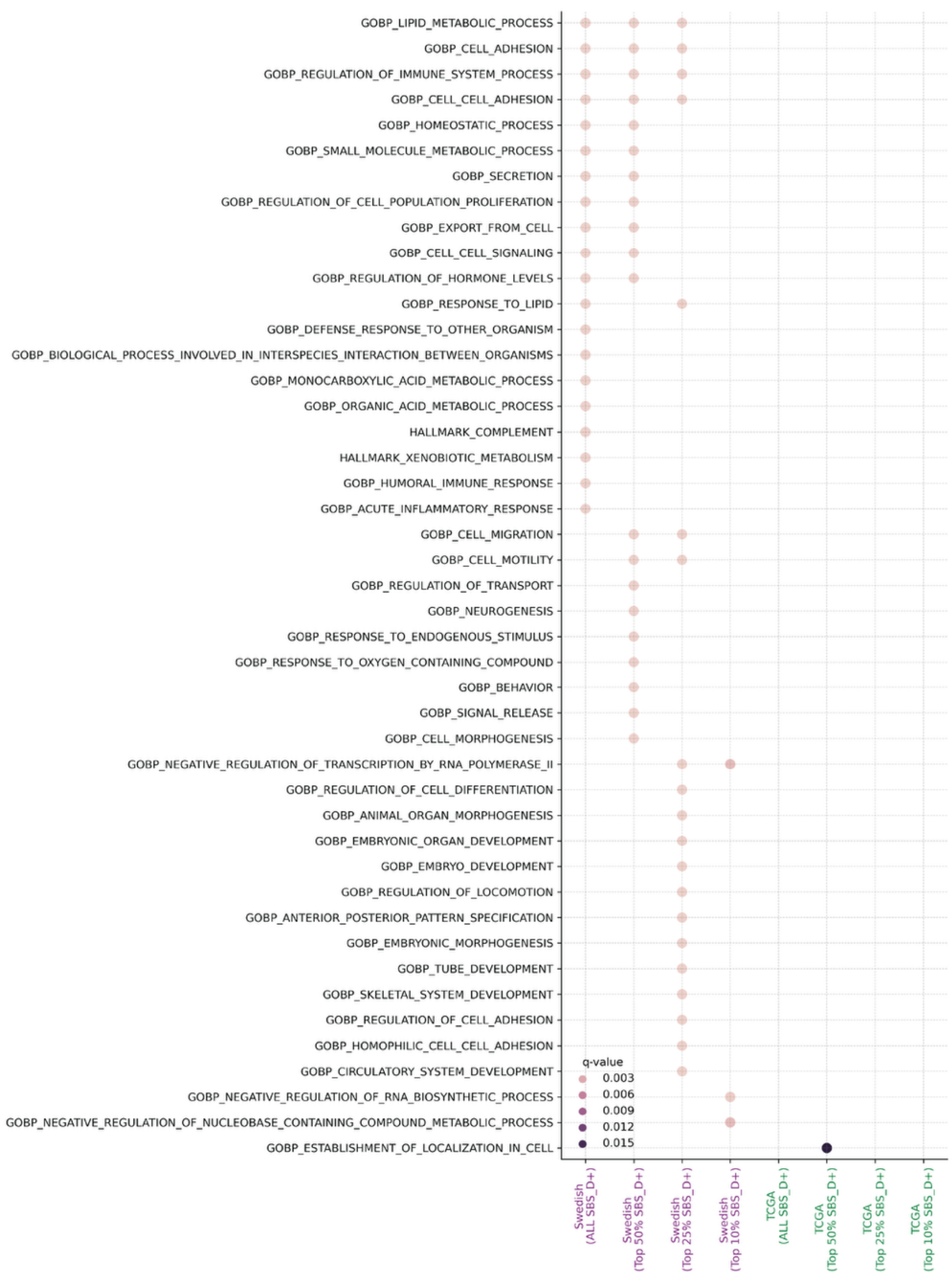

Response Figure 4.3: Pathways enriched among underexpressed genes in SBS_D+ samples in the TCGA and Swedish cohorts. List of pathways significantly enriched among underexpressed genes, identified by Gene Set Enrichment Analysis (GSEA) in the Swedish (purple) and TCGA (green) cohorts. Analyses include comparisons of all SBS_D+ samples, as well as the top 50%, 25%, and 10% of SBS_D+ samples, against all SBS_D- samples. Hallmark and GO Biological Process gene sets were used. Each point represents a comparison in which a gene set was significantly enriched among underexpressed genes, with color indicating the corresponding q-value. No gene sets were consistently enriched among underexpressed genes across both the Swedish and TCGA cohorts.

Lastly, we have added a section to the manuscript clarifying that SBS_D represents a novel COSMIC mutational signature (provisionally designated SBS111) of unknown etiology, with genomic features consistent with a hypothesis of POLD1-associated repair infidelity.

RESULTS

*"**COSMIC Classification and Etiology of SBS_D***

The consistent identification of SBS_D across multiple independent cohorts, together with its distinct mutational profile and orthogonal biological features, supports its classification as a novel mutational signature within the COSMIC reference framework. While naïve decomposition suggests similarity to signatures associated with POLD1 proofreading deficiency and mismatch repair deficiency, the absence of genomic or phenotypic evidence of these defects indicates that SBS_D does not arise from canonical DNA repair deficiency. Accordingly, SBS_D is classified as a mutational signature of unknown etiology under COSMIC conventions. SBS_D has been submitted to the COSMIC database and is provisionally designated SBS111. In the COSMIC annotation, it is noted that, although SBS_D does not reflect canonical DNA repair deficiency, its genomic features are consistent with a hypothesis of infidelity in these DNA repair processes."

Comment: 2) Furthermore, analysis of associations with clinicopathological features (in response to comment 6) was based on categorizing SBS_D activity as a binary 'yes/no' feature. Could the authors explore potential associations using SBS_D activity as a continuous score, and evaluate potential characteristics of the high-scoring samples? (This is also related to previous comment 4 and the new graphs in Supplementary Figure 5 showing varying contributions from SBS_D among the SBS_D positive samples.)

Response: We agree with the reviewer that treating SBS_D activity as a continuous variable provides an important complement to the binary analyses presented in the prior version of the manuscript. Because approximately 50% of samples have zero SBS_D activity, we applied a hurdle modeling framework, which is well suited for outcomes with excess zeros and jointly models the presence/absence of SBS_D activity and its magnitude when present. As part of this framework, we previously reported logistic regression analyses treating SBS_D as a binary variable (presence/absence; **Supplementary Figure 6**). In response to this comment, we have now additionally performed linear regression analyses using SBS_D activity as a continuous outcome to evaluate associations with clinicopathological features, including age, sex, tumor site, stage, and grade.

Using this approach, we identified a positive association between SBS_D activity and age in the Mutographs cohort (**Response Figure R4.4*a***; see next page). We also observed apparent country-level associations; however, these were dependent on the choice of reference country (e.g., relative

to Brazil), and no country-level associations were detected when alternative reference groups were used (e.g., relative to Iran), while all other covariate associations remained unchanged. This was further confirmed by fitting a model per country in a one-versus-all approach, which yielded no significant associations between SBS_D and any country. A positive association between SBS_D activity and age was also observed in TCGA (**Response Figure R4.4*b***). Finally, no clinicopathological features were associated with SBS_D activity in the Swedish cohort (**Response**

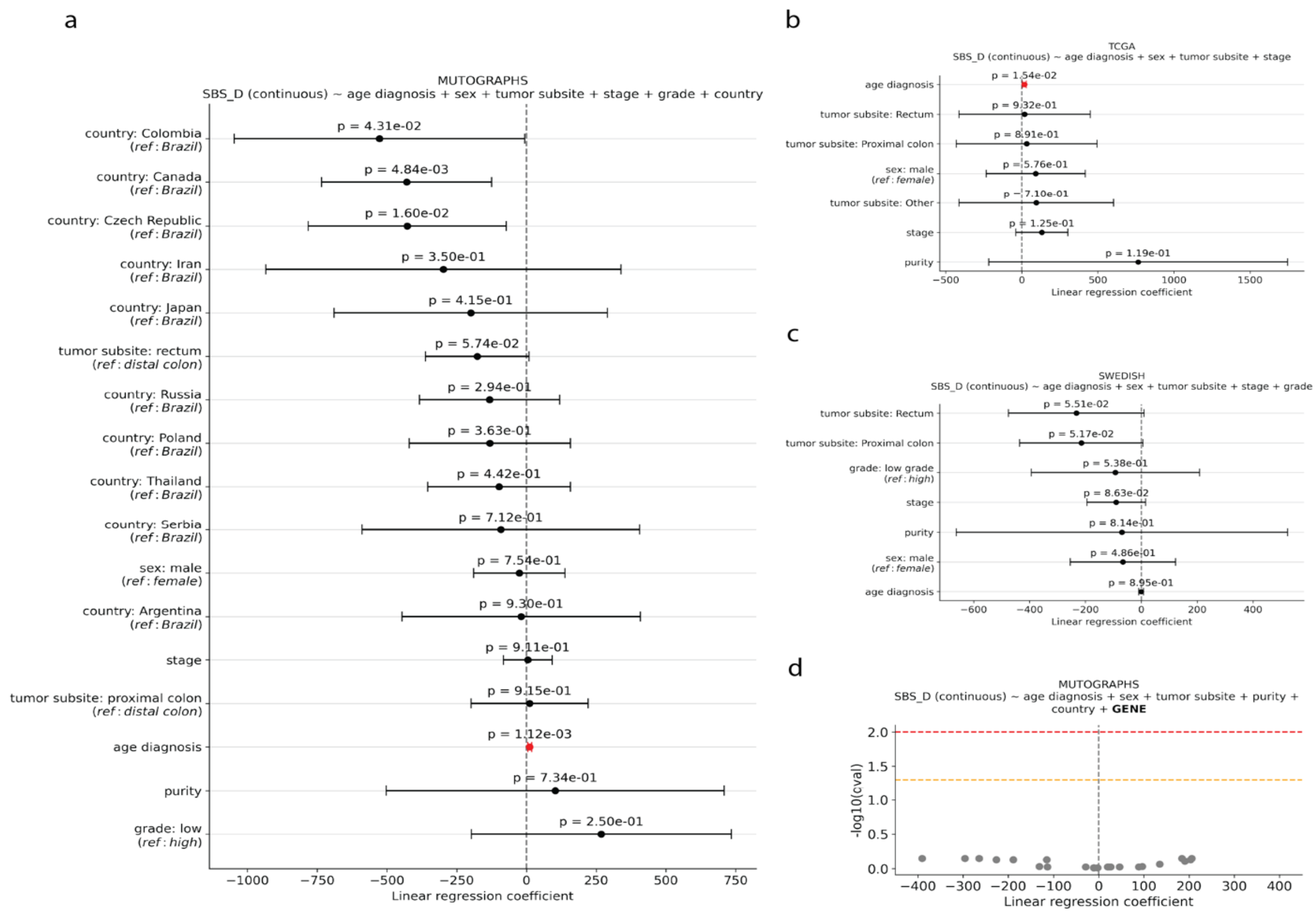

Response Figure 4.4: Associations between SBS_D activity and clinicopathological features using SBS_D as a continuous measure. ***(a)*** Results from a linear regression model applied to SBS_D+ samples in the Mutographs colorectal cancer (CRC) cohort, with SBS_D activity as the outcome variable and age of diagnosis, sex, tumor subsite, purity, stage, grade, and country included as covariates. Points indicate effect estimates for each covariate, with whiskers representing 95% confidence intervals; corresponding p-values are shown. Whiskers for clinicopathological features (age, sex, tumor site, stage, and grade) with p-values < 0.05 are highlighted in red. ***(b)*** As in *(a)*, but for TCGA CRC cohort with age of diagnosis, sex, tumor subsite, purity, and stage as covariates. ***(c)*** As in *(a)*, but for the Swedish CRC cohort with age of diagnosis, sex, tumor subsite, purity, stage, and grade as covariates. ***(d)*** Volcano plot for the Mutographs CRC cohort showing associations between SBS_D activity and driver gene mutations present in at least 20 of 802 samples. For each gene, a separate linear regression model was fit with SBS_D activity as the outcome variable and age at diagnosis, sex, tumor subsite, tumor purity, and mutation status of the respective gene (mutated versus not mutated) included as covariates. P-values were adjusted using the Benjamini–Hochberg procedure. The orange and red lines denote q-value thresholds of 0.05 and 0.01, respectively.

Figure R4.4*c*). Additionally, no driver gene mutations were associated with SBS_D activity (**Response Figure R4.4*d***).

Overall, across both logistic and linear regression models, age was the only clinicopathological feature that showed a consistent positive association with SBS_D activity in more than one cohort, indicating that higher SBS_D activity is more frequently observed in older individuals. Because DNA repair mechanism efficiency is thought to decrease as individuals age (PMID: 17913742) and we have hypothesized that SBS_D may be related to infidelity in DNA repair, it may be the case that older patients with SBS_D+ tumors have a modest increase in the number of SBS_D mutations compared to younger patients due to preexisting vulnerabilities in their DNA repair processes. No other clinicopathological features showed reproducible associations across cohorts. These results are now included as **Supplementary Figure 7**, and the *Results* and *Discussion* sections has been updated accordingly.

RESULTS

*" **Investigating Features Associated With SBS_D+ Samples***

*We investigated associations between SBS_D activity and standard clinical and pathological features, such as age of diagnosis, tumor subsite, or tumor stage (**Methods**). Across both binary and continuous modeling approaches, a modest positive association between SBS_D activity and age at diagnosis was the only relationship observed in more than one cohort (**Supplementary Figures 6-7**). No other clinicopathological features demonstrated reproducible associations with SBS_D activity across cohorts. Similarly, mutations in previously identified driver genes[9] were not associated with SBS_D (**Supplementary Figure 6d and Supplementary Figure 7d**)."*

…

*"Of note, DNA repair capacity is presumed to decline with age[29,30], and our prior observation of a modest positive association between SBS_D activity and age at diagnosis may further reflect a connection between SBS_D and DNA repair (**Supplementary Figures 6-7**)."*

DISCUSSION

"Although RNA expression analyses and associations with clinicopathological features did not yield conclusive insights into SBS_D etiology, our genomic-level analyses suggest that SBS_D may reflect DNA repair infidelity during the late stages of CRC evolution."

<u>Comment:</u> 3) Finally, reanalysis of late clonal mutations only (enriched in SBS_D) could help to unmask any potential associations not identified when also including early clonal events in the analysis. For example, is the sample-wise proportion of SBS_D among late clonal mutations associated with any clinicopathological features or driver mutations?

Response: We thank the reviewer for this helpful suggestion. To evaluate whether restricting the analysis to late clonal mutations reveals additional associations, we examined the sample-wise proportion of SBS_D among late clonal mutations in SBS_D+ samples. First, to assess potential associations with driver gene alterations, we fitted multiple linear regression models (one per gene), using the proportion of SBS_D as the outcome variable and age, sex, tumor subsite, country, tumor purity, and the mutation status of the respective gene (mutated versus not mutated) as covariates. Consistent with the analyses presented in the manuscript, no significant associations with driver gene mutations were identified (**Response Figure R4.5*a***).

We next evaluated associations between the proportion of SBS_D among late clonal mutations and clinicopathological variables. Interestingly, we observed a positive relationship between SBS_D proportion and stage, as well as a negative association between SBS_D proportion and high-grade status (**Response Figure R4.5*b***). While this observation may suggest a potential relationship, it should be interpreted with caution. One possible explanation is that tumor stage and grade capture distinct biological dimensions: stage reflects the extent of tumor progression over time, whereas

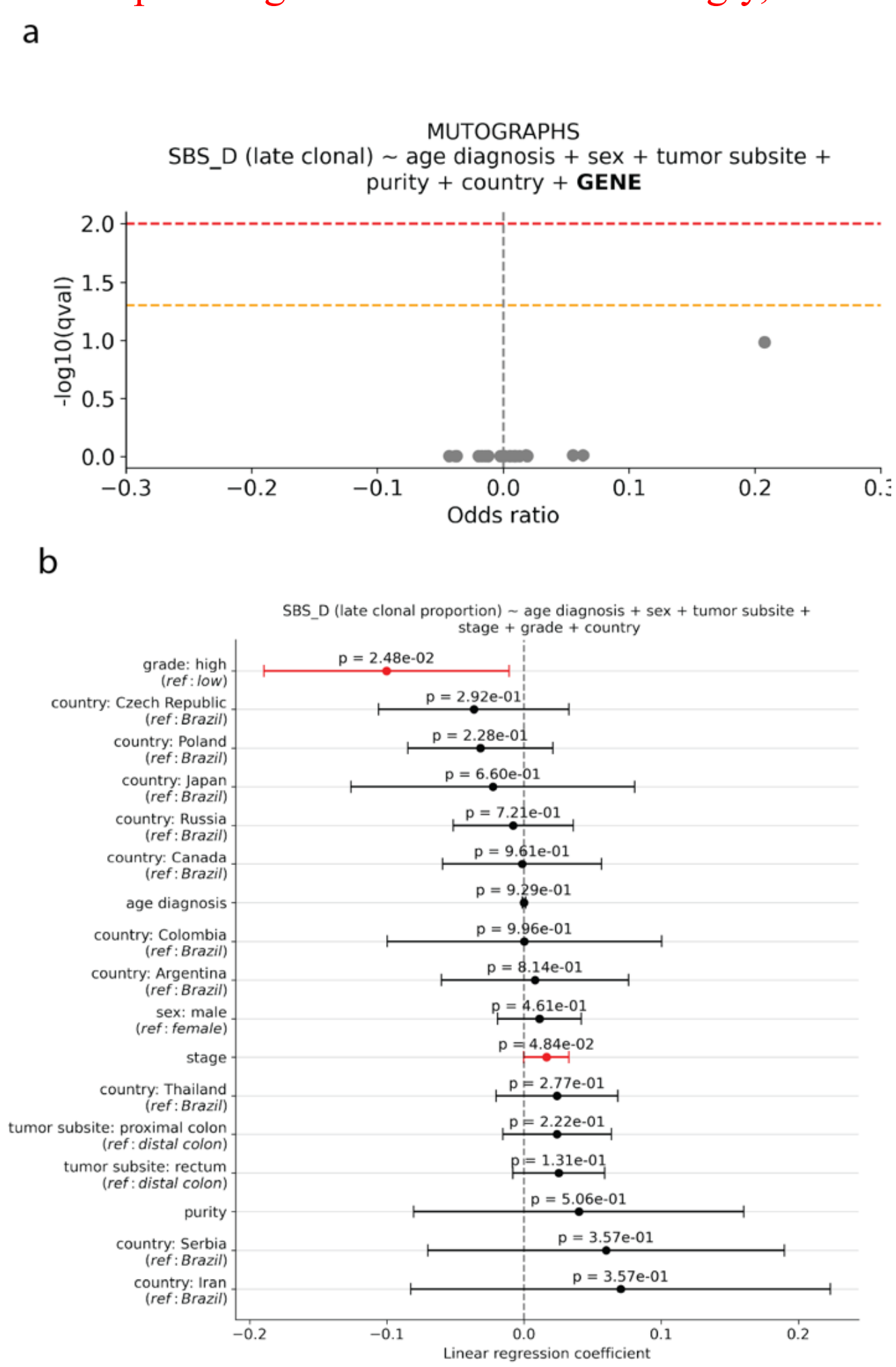

Response Figure 4.5: Associations in the Mutographs colorectal cancer cohort using the sample-wise proportion of SBS_D among late clonal mutations. *(a)* Volcano plot showing associations between SBS_D activity among late clonal mutations and driver gene mutations in the Mutographs colorectal cancer (CRC) cohort. Each point represents a driver gene mutated in at least 20 of the 802 samples. For each gene, a separate linear regression model was fitted with the proportion of SBS_D among late clonal mutations as the outcome variable and age at diagnosis, sex, tumor subsite, tumor purity, country, and mutation status of the respective gene (mutated vs. not mutated) included as covariates. P-values were adjusted using the Benjamini-Hochberg procedure. The orange and red lines denote q-value thresholds of 0.05 and 0.01, respectively. ***(b)*** Results from a linear regression model applied to SBS_D+ samples in the Mutographs CRC cohort, with the proportion of SBS_D among late clonal mutations as the outcome variable and age at diagnosis, sex, tumor subsite, tumor purity, stage, grade, and country included as covariates. Points indicate effect estimates for each covariate, with whiskers representing 95% confidence intervals; corresponding p-values are shown. Clinicopathological features (age, sex, tumor subsite, stage, and grade) with p-values < 0.05 are highlighted in red.

grade reflects cellular differentiation and proliferative aggressiveness. SBS_D may therefore preferentially accumulate as tumors progress without being enriched in poorly differentiated, high-grade tumors. At the same time, evolutionary timing analyses are inherently constrained by tumor-specific copy number histories. Late clonal mutations are defined relative to large-scale copy number gains, and tumors that experience such gains at different times or to different extents will exhibit highly variable late clonal mutational profiles and signature proportions. Consequently, while evolutionary analyses are useful for identifying which mutational processes tend to act early or late, direct comparisons of late clonal signature activity across samples are subject to important caveats and should be interpreted carefully.

Comment: 4) Previous comment 11: redundancy in text. The revised manuscript is occasionally repetitive and there is potential to improve this, particularly for the text recapitulating data and interpretations from the preceding and previously published study (reference 9, Diaz-Gay et al.).
Response: We thank the reviewer for this comment. We have carefully edited the revised manuscript to reduce redundancy and streamlined the text accordingly.

Comment: 5) For the purpose of data reproducibility, the authors should consider sharing the mutation signature activities calculated per sample in the external cohorts.
Response: We are grateful to the reviewer for the suggestion. To facilitate data reproducibility, we have now included **Supplementary Tables 1-8** reporting the mutational signature activities calculated per sample and the corresponding signatures across all used cohorts.

Reviewer #5 (Remarks to the Author):
I co-reviewed this manuscript with one of the reviewers who provided the listed reports. This is part of the Nature Communications initiative to facilitate training in peer review and to provide appropriate recognition for Early Career Researchers who co-review manuscripts.
Response: We thank Reviewer #5 for the time and effort invested in co-reviewing this manuscript.

Color/Font Legend: Reviewers' Comments, Authors' Responses, *Revised Manuscript Excerpts*

Reviewer #4 (Remarks to the Author):

We thank the authors for the additional data and analyses. All previous comments have been addressed, and we consider the new data to have strengthened the study. We appreciate that the authors have submitted the new SBS_D signature to the COSMIC database under the classification of unknown etiology.

Response: We thank the reviewer for the positive evaluation and for recommending our manuscript for publication in *Nature Communications*. We appreciate the time and constructive feedback provided during the review process.

Reviewer #5 (Remarks to the Author):

I co-reviewed this manuscript with one of the reviewers who provided the listed reports. This is part of the Nature Communications initiative to facilitate training in peer review and to provide appropriate recognition for Early Career Researchers who co-review manuscripts.

Response: We thank Reviewer #5 for the time and effort invested in co-reviewing this manuscript.
